# A large interactive visual database of copy number variants discovered in taurine cattle

**DOI:** 10.1093/gigascience/giz073

**Published:** 2019-06-26

**Authors:** Arun Kommadath, Jason R Grant, Kirill Krivushin, Adrien M Butty, Christine F Baes, Tara R Carthy, Donagh P Berry, Paul Stothard

**Affiliations:** Department of Agricultural, Food and Nutritional Science (AFNS), University of Alberta, Edmonton, AB, Canada; Lacombe Research and Development Centre, Agriculture and Agri-Food Canada, Lacombe, Alberta, Canada; Department of Agricultural, Food and Nutritional Science (AFNS), University of Alberta, Edmonton, AB, Canada; Department of Agricultural, Food and Nutritional Science (AFNS), University of Alberta, Edmonton, AB, Canada; Centre for Genetic Improvement of Livestock, Department of Animal Biosciences, University of Guelph, Guelph, ON, Canada; Centre for Genetic Improvement of Livestock, Department of Animal Biosciences, University of Guelph, Guelph, ON, Canada; Institute of Genetics, Vetsuisse Faculty, University of Bern, Bern, Switzerland; Teagasc, Animal & Grassland Research and Innovation Centre, Moorepark, Fermoy, Ireland; Teagasc, Animal & Grassland Research and Innovation Centre, Moorepark, Fermoy, Ireland; Department of Agricultural, Food and Nutritional Science (AFNS), University of Alberta, Edmonton, AB, Canada

**Keywords:** CNV, structural variants, cattle, dairy, beef, whole-genome sequencing, database, sequence visualization

## Abstract

**Background:**

Copy number variants (CNVs) contribute to genetic diversity and phenotypic variation. We aimed to discover CNVs in taurine cattle using a large collection of whole-genome sequences and to provide an interactive database of the identified CNV regions (CNVRs) that includes visualizations of sequence read alignments, CNV boundaries, and genome annotations.

**Results:**

CNVs were identified in each of 4 whole-genome sequencing datasets, which together represent >500 bulls from 17 breeds, using a popular multi-sample read-depth−based algorithm, cn.MOPS. Quality control and CNVR construction, performed dataset-wise to avoid batch effects, resulted in 26,223 CNVRs covering 107.75 unique Mb (4.05%) of the bovine genome. Hierarchical clustering of samples by CNVR genotypes indicated clear separation by breeds. An interactive HTML database was created that allows data filtering options, provides graphical and tabular data summaries including Hardy-Weinberg equilibrium tests on genotype proportions, and displays genes and quantitative trait loci at each CNVR. Notably, the database provides sequence read alignments at each CNVR genotype and the boundaries of constituent CNVs in individual samples. Besides numerous novel discoveries, we corroborated the genotypes reported for a CNVR at the *KIT* locus known to be associated with the piebald coat colour phenotype in Hereford and some Simmental cattle.

**Conclusions:**

We present a large comprehensive collection of taurine cattle CNVs in a novel interactive visual database that displays CNV boundaries, read depths, and genome features for individual CNVRs, thus providing users with a powerful means to explore and scrutinize CNVRs of interest more thoroughly.

## Introduction

Structural variants, originally defined to include insertions, deletions, and inversions >1 kb in size [1], now encompass events as small as 50 bp [2]; this change in definition is likely due, in part, to developments in sequencing technology that greatly improved the resolution of discovery achievable. Copy number variants (CNVs) are a class of unbalanced structural variants characterized by changes to the number of base pairs in the genome and manifested as gains or losses of regions of genomic sequence between individuals of a species; CNVs therefore contribute to genetic diversity. Several examples have been reported of CNVs associated with normal variation, disease, evolution, and adaptive traits in human, animal, and plant species [3–7]. With next-generation sequencing (NGS) technology becoming more cost-effective, traditional methods for CNV discovery that involved hybridization-based microarray approaches like array comparative genomic hybridization (CGH) and single-nucleotide polymorphism (SNP) microarrays are now being replaced by powerful sequencing-based computational approaches.

Studies on CNV discovery and characterization have been performed on several farm animal species [8–14] with the ultimate objective of using variants that are associated with traits of economic importance in genetic improvement programs. In cattle, several studies [15–29] have been conducted, in both taurine and indicine breeds, using a variety of algorithms to identify thousands of CNVs. While attempts have been made to provide overall assessments on the reliability of CNV regions (CNVRs) reported in some of those studies using such approaches as parent-offspring trios [9], PCR [8], or a combination of *in silico* and experimental techniques [21], the majority have been limited to providing the CNVR boundaries alone. Assessing the potential impact of CNVRs at individual and population levels becomes difficult in the absence of genotypes and boundaries of CNVs constituting CNVRs in individual samples. A recent study [30] has proposed the use of BAM confirmation (i.e., visually examining read depth and read pairing characteristics) as a strategy to assess the accuracy of predicted CNVRs. This approach was then applied to a limited number of CNVs selected on the basis of overlap with certain human disease-associated genes [30]. Couldrey et al. [31] illustrated the use of long-read sequence information combined with a CNV transmission-based approach to confirm a subset of CNVs that segregate in the New Zealand dairy cattle population. Briefly, the putative CNVs discovered from long-read sequence information in a prominent Holstein-Friesian bull used in New Zealand were first compared with those discovered from short-read sequences in the same bull. Next, a population of 556 cattle representing the wider New Zealand dairy cattle population were short-read sequenced and genotyped at those putative CNV regions, followed by a genome-wide assessment of transmission level of copy number based on pedigree. Visual assessment of highly transmissible CNV regions provided additional evidence to support the presence of CNV across the sequenced animals. Currently, the high cost of long-read sequencing limits adoption of this approach to large numbers of animals representing different breeds, and other studies that provide supportive evidence on a genome-wide scale to help assess the quality of CNVs predicted from short-read sequencing or SNP array data are extremely limited.

The objectives of the present study were to identify and characterize genome-wide CNVRs among popular taurine cattle (*Bos taurus*, NCBI:txid9913) breeds and to present the results in a comprehensive interactive database of CNVRs and copy number genotypes, integrated with visualizations of sequence read alignments and genome features. Briefly, CNVs were identified in each of 4 available whole-genome sequencing (WGS) datasets, which together represented 553 bulls from 17 different breeds (1 dairy and 16 beef breeds). We used cn.MOPS [32], a popular CNV detection software that employs a multi-sample read-depth−based algorithm to estimate copy number genotypes per sample. Custom software was then used to convert the results for each dataset into an interactive visual database, a first of its kind for genome-wide CNVR data in any species. The databases, which can be downloaded and then opened using a modern web browser, give users the ability to assess each CNVR with supportive evidence and multiple levels of genome annotation. Further advantages of this format include, for example, the ability to adjust filtering criteria, compare CNV boundaries and genotypes across samples, and search for affected genes or regions of interest.

## Results

### Adverse influence of batch effects on CNV discovery from combined datasets

We obtained WGS data on a total of 553 bulls from 4 different sources; all were paired-end sequenced but differed in the sequencing platform used as well as the coverage, read length, sample size, and breed representation (Table 1). Detailed information on samples and sources of sequence data are provided in Supplemental Table S1. Dataset A was generated using the SOLiD platform and had lower read length and mean coverage (Supplemental Fig. S1) than datasets generated using the Illumina platform.

**Table 1:** Sequencing and sample characteristics per dataset

Dataset (year sequenced)	Platform (read length)	Coverage mean (SD)	Total samples	Breed codes * (No. of samples)
A (2012–13)	SOLiD 5500xl (75 × 35 bp)	7× (4.6)	85	SIM (30), LIM (28), CHA (16), BBR (8), GVH (3)
B (2013–14)	Illumina HiSeq 2000 (100 bp)	11.6× (3.3)	298	HOL (48), AAN (47), SIM (35), HER (33), GVH (28), RAN (26), CHA (25), BBR (16), XXX (14), PIE (7), RDP (7), LIM (6), HYB (3), BAQ (1), DEV (1), SAL (1)
C (2016)	Illumina HiSeq X (150 bp)	10.3× (2.6)	138	CHA (42), LIM (30), SIM (27), AAN (15), HER (15), BBL (9)
D (2017)	Illumina HiSeq X Ten (150 bp)	37.9× (3.6)	32	HOL (32)

^*^The breed codes used for purebred cattle follow the guidelines provided by the International Committee for Animal Recording (ICAR) for identification of semen straws for international trade. In addition, XXX represents crossbred cattle and HYB represents composite breeds other than BBR. AAN: Angus; BAQ; Blonde D'Aquitaine; BBL: Belgian Blue; BBR: Beef Booster; CHA: Charolais; DEV: Devon; GVH: Gelbvieh; HER: Hereford; HOL: Holstein; LIM: Limousin; PIE: Piedmontese; RAN: Red Angus; RDP: Rouge des Prés; SAL: Salers; SIM: Simmental.

**Figure 1:**
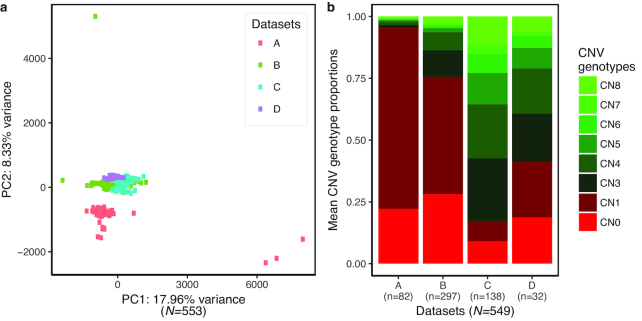
Batch effects amongst the 4 datasets contributing to inconsistent distribution of CNV genotypes in the analysis of the combined datasets. (a) PCA based on normalized read counts per segment showed separation by datasets and 4 outliers. (b) When datasets were combined and analysed together using cn.MOPS (*N* = 549 after removing PCA outliers), the distribution of CNV genotypes revealed considerable differences among datasets (only autosomal CNVs are depicted here).

Using aligned sequence data from all bulls simultaneously as input into cn.MOPS, we assessed counts of reads aligned to each non-overlapping window across the genome. The window length (WL) was chosen such that each segment comprised on average 100 reads, as is recommended in cn.MOPS documentation. A WL of 1,000 bp satisfied this criterion for datasets A−C. For uniformity, we chose to keep the same WL for dataset D, despite the fact that it had substantially greater sequencing coverage (Table 1) and would have allowed for a lower WL. The CNV discovery algorithm implemented in cn.MOPS derives its power from modelling read count variability across samples, and therefore read count normalization was performed as a prerequisite. A principal component analysis (PCA) on the normalized read counts per segment across samples revealed clear separation amongst datasets, which was indicative of uncorrected batch effects (Fig. 1a). Proceeding with CNV discovery and genotype characterization using those read counts from all datasets together (after excluding the 4 PCA outliers) revealed considerable differences in the distribution of CNV genotypes per dataset (Fig. 1b). The genotype distributions were skewed towards deletion type (DEL) CNVs in datasets A and B (datasets with comparatively lower read lengths) as opposed to datasets C and D where the distributions were skewed towards amplification (AMP) type CNVs. These aberrations may arise from the presence of more regions of limited or no coverage in datasets A and B, which triggered false DEL type CNV genotype calls when compared across corresponding regions in other datasets with adequate coverage due to longer read length or advances in sequencing technology. Together, these results indicated the necessity to analyse distinct datasets individually with additional dataset-specific filters applied to identify and remove outlier samples.

### Distributions of CNV genotypes were more consistent across datasets that were analysed individually

**Figure 2:**
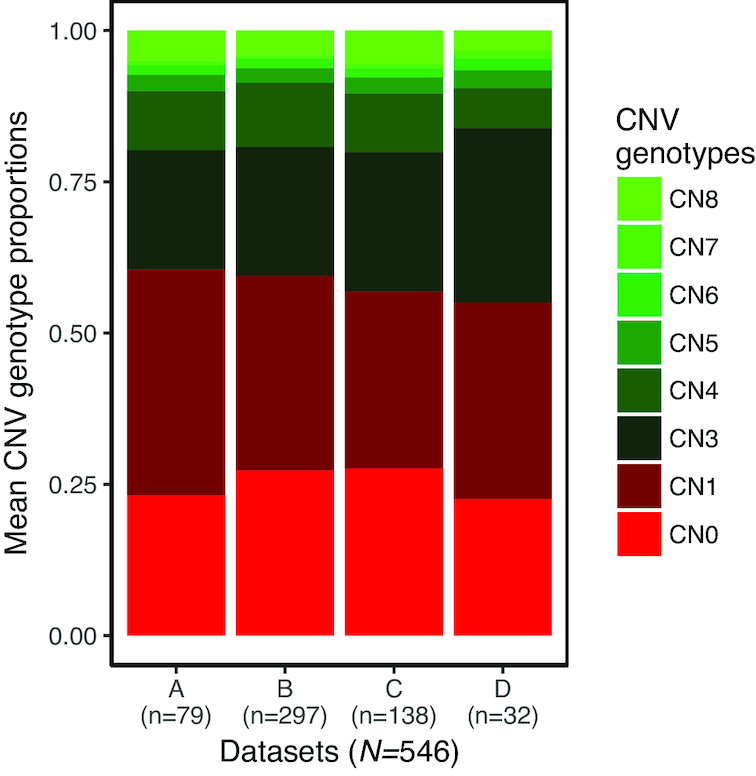
Distributions of CNV genotypes were more consistent across datasets that were analysed individually. When datasets were analysed individually (*N* = 546 after removing PCA outliers and high-coverage outlier samples in dataset A), the distribution of CNV genotypes was consistent among datasets (only autosomal CNVs are depicted here).

**Figure 3:**
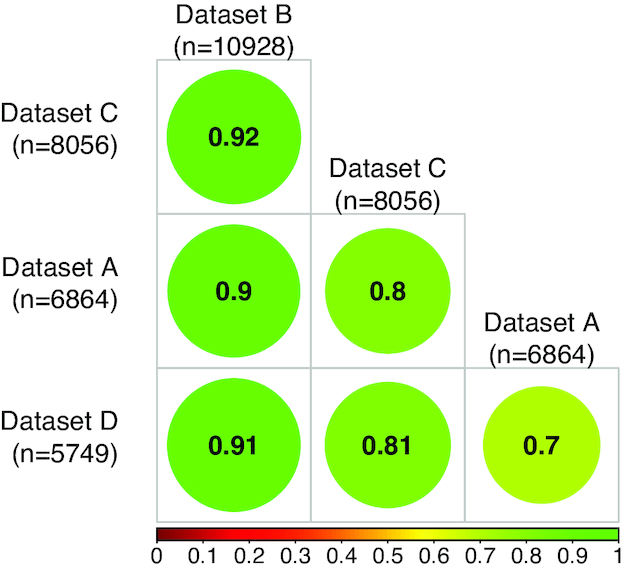
Proportions of overlapping CNVRs amongst datasets. Pairwise comparisons of the proportions of CNVRs in each dataset (rows; ordered by dataset size) that overlap by ≥1 base pair with CNVRs of other larger datasets (columns) are presented.

To avoid the adverse influence of batch effects on CNV discovery with cn.MOPS when combining datasets with genomic regions of imbalanced coverage, we analysed each dataset individually. Using cn.MOPS, CNVs were identified after first excluding the 4 PCA outliers (3 in dataset A and 1 in dataset B; see Fig. 1b) and 3 samples within dataset A that were of substantially higher coverage than the others within that dataset (Supplemental Fig. S1). Contrary to what was observed when datasets were combined, the proportions of DELs among CNVs were quite consistent among datasets analysed individually (Fig. 2), with the mean proportion of DELs ranging between 0.55 (SD, 0.08) for dataset D and 0.61 (SD, 0.09) for dataset B. Additional quality control (QC) steps were applied to identify problematic samples, defined as those that showed marked deviations (i.e., 1.5 times the interquartile range away from the first and third quartiles) in the proportion of DELs or total CNVs discovered within each dataset. The total number of problematic samples identified were 7, 10, 7, and 3, respectively, for datasets A−D. For dataset A, most of the problematic samples identified were amongst the lowest coverage samples (coverage <5×) while for the other datasets with higher coverage, such a trend was not clearly evident. Plots per dataset that indicate the proportion of the different CNV genotypes identified per sample, distributions of CNV genotype counts, proportion of DELs among CNVs, and total CNVs discovered are provided in Supplemental Figs S2−S5 with problematic samples labelled. All CNVs called within problematic samples were removed, which improved the consistency among datasets, with means of the proportion of DELs ranging between 0.57 (SD, 0.06) for dataset C and 0.60 (SD, 0.07) for dataset B. The CNVs, from the 519 samples that remained after QC, were used to construct CNVRs per dataset based on a 50% reciprocal overlap criterion, consistent with the procedure used elsewhere [18, 21]. Finally, refined sets of CNVRs were obtained after filtering out CNVRs observed in only 1 sample per dataset. Based on the genotypes of constituent CNVs, the CNVRs were categorized as DEL (CN0/CN1), AMP (CN3+), or mixed (MIX) type (1 or more of CN0/CN1 and CN3+). Dataset-wise hierarchical clustering of samples based on the CNVR genotypes (representative genotype of CNVs making up each CNVR; see Methods) revealed clear clustering by breeds (Supplemental Figs S6−S9) as expected.

A list of CNVRs discovered in each dataset with the respective CNVR category assignments is provided in Supplemental Table S2. The list consists of a total of 26,223 unique CNVRs, counting those with identical genomic coordinates across datasets only once. The dataset-wise counts of CNVs and CNVRs and the non-redundant genome length covered by CNVRs (Table 2) were all proportional to the sample sizes of the individual datasets. These relationships were as expected and were also observed at the breed level (breed-wise summaries of CNVRs are provided in Supplemental Table S3). Notably, dataset B had the greatest number of CNVRs in total, which may be attributed to its larger sample size and diversity of breeds, which included purebreds, crossbreds, and composites. Conversely, dataset D had the lowest genome coverage by CNVRs, which may be attributed to the fact that it comprised only 1 breed and thus less genomic variability compared with the other datasets with multiple breeds. These differences amongst datasets were also reflected in the chromosome-wise counts of total CNVRs of each category where datasets of larger sample size and breed diversity revealed higher proportions of MIX category CNVRs (Supplemental Fig. S10a−d; lower panel). Chromosomes 12, 15, 14, and 29 had comparatively higher density of CNVRs (CNVR counts per megabase over the third quartile in all datasets) than others whereas chromosomes 2, 11, 13, 24, and 22 were amongst the least dense (Supplemental Fig. S10a−d; upper panel). Phenograms representing the chromosomal locations of CNVRs belonging to the different categories indicate distinct patterns broadly conserved across datasets (Supplemental Fig. S11a−d).

**Table 2:** Dataset-wise summary of CNVs and CNVRs

Dataset	No. post-QC (No. pre-QC)			CNVRs per category (No. of DELs; AMPs; MIX)	Size of largest CNVR (kb)	Non-redundant size of genome (Mb) covered by CNVRs (%)
	Samples	CNVs	CNVRs			
A	72 (79)	35,531 (41,673)	6,864 (11,625)	2,012; 2,660; 2,192	378	53.85430 (2.02)
B	287 (297)	103,040 (117,104)	10,928 (19,139)	2,687; 4,646; 3,595	950	92.48615 (3.48)
C	131 (138)	54,797 (61,050)	8,056 (12,351)	2,522; 2,793; 2,741	501	65.90313 (2.48)
D	29 (32)	17,790 (20,107)	5,749 (8,988)	1,911; 1,845; 1,993	580	44.47765 (1.67)
Summary	519 (546)	157,862 (182,355)	26,223 (44,836)	9,974; 8,302; 9,115	950	107.74670 (4.05)

For the summary, the non-redundant size of genome covered was obtained by merging overlapping or adjacent CNVRs across datasets whereas the numbers of CNVs, CNVRs, and CNVRs per category were obtained by counting CNVRs with unique genomic coordinates.

### Overlaps between CNVRs identified in the 4 datasets were low when compared with those reported in previous studies but high between the datasets themselves

Previous studies that compared CNVRs discovered across studies reported a low percentage of overlap, which is attributable to the numerous differences among studies, e.g., sample size and characteristics, sequencing platform and technology, and CNV detection algorithm. In cattle, the percentage of overlap among CNVRs discovered across multiple studies was generally <40% [3, 18], with overlapping CNVRs defined as those that share ≥1 base position. In agreement, the percentage of overlap between the CNVRs detected in the 4 datasets of the present study and those detected in previous studies was generally low, ranging between 22% and 35% on average (Table 3). A merged list of CNVRs from the 4 datasets consisted of 9,482 CNVRs (mean CNVR size, 11.363 kb; largest CNVR size, 3.152 Mb), of which, on average, 37% overlapped with the CNVRs identified in previous studies (Table 3; ABCD). The list was generated by merging overlapping or adjacent CNVRs across datasets as was performed earlier to determine the overall non-redundant size of genome covered by CNVRs (see Table 2). Surprisingly, in another comparison limited to the 4 datasets, between 70% and 92% of the CNVRs detected in the smaller datasets (A, C, and D) overlapped with CNVRs in dataset B, the dataset with the largest sample size and breed representation (Fig. 3). Despite the differences amongst the 4 datasets, the high degree of overlap between CVNRs identified could point to the choice of the CNV detection algorithm being the factor that contributes most to variability in CNVs discovered across studies.

**Table 3:** Overlaps between CNVRs identified in this study and those from previous published reports

Study	Platform	No. chr.	No. breeds, samples, and CNVRs	% Overlap with CNVRs identified in this study				
				A	B	C	D	ABCD
Fadista et al. [15]	CGH-based	29+X	4; 20; 266	12.0	16.9	13.9	11.3	18.0
Liu et al. [16]		29+X	17; 90; 223	65.5	78.0	71.7	57.4	78.9
Hou et al. [22]	SNP-based (50K chip)	29	21; 521; 743	35.8	48.0	35.1	30.6	51.1
Bae et al. [23] *		29	1; 265; 224	16.5	29.0	14.3	10.3	33.9
Hou et al. [24]		29	1; 472; 500	21.0	31.8	21.0	16.6	35.6
Jiang et al. [25]		22	1; 2,047; 64	31.2	48.4	25.0	21.9	48.4
Hou et al. [26]	SNP-based (HD chip)	29	27; 674; 3,438	19.4	28.4	20.5	15.4	33.0
Wu et al. [27]		29+X	1; 792; 263	38.8	49.8	39.2	29.3	54.4
Bickhart et al. [28]	WGS	29	3; 5; 763	10.6	14.4	11.1	9.3	16.0
Zhan et al. [29]		29	1; 1; 419	8.1	11.5	8.4	9.5	13.8
Stothard et al. [17]		26	2; 2; 634	12.3	15.1	13.2	11.7	16.2
Keel et al. [18]		29+X	7; 154; 1,341	60.8	66.4	64.0	56.3	67.2
Chen et al. [19]		29+X	2; 316; 16,325	6.7	10.7	8.1	5.5	12.2
Mean % overlap				26.05	34.49	26.58	21.93	36.82
No. of breeds, samples, and CNVRs identified in this study				5; 72; 6,864	16; 287; 10,928	6; 131; 8,056	1; 29; 5,749	17; 517; 9,482

^*^For studies that used the Btau 4.0 assembly for mapping, we used the UCSC liftOver tool [33] to convert the genomic coordinates of the CNVRs to UMD 3.1.

### Identification and genotyping of the well-characterized *KIT* locus CNV in our datasets

**Figure 4:**
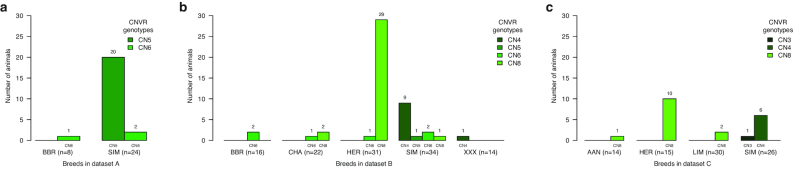
Prevalence and genotypes of the *KIT* locus CNV across breeds and datasets. The breed-wise prevalence and genotypes at CNVR Chr6:71,747,001–71,752,000, found ~45 kb upstream of the *KIT* gene, are depicted here. This CNVR has been reported to be associated with the piebald coat colour phenotype in HER and some SIM cattle, and occurs in high copy numbers in these breeds. The reason for detection of this CNVR in high copy number in 2 of the 22 CHA cattle in dataset B is attributed to potential issues with sourcing or handling of the respective samples.

A CNVR at Chr6:71,747,001–71,752,000, found ~45 kb upstream of the *KIT* gene (Chr6:71,796,318–71,917,431), has been reported to be associated with the piebald coat colour phenotype in HER and some SIM cattle [34–36], but not the dorsal spotting on SIM and HOL cattle or the white patterning on Rouge des Prés [36] (RDP; formerly called Maine-anjou). Because this was one of the few breed-associated cattle CNVs with available genotypes described in the literature, we looked at whether our analysis produced consistent breed specificity and genotypes at the *KIT* locus CNVR. Overall, we found (Fig. 4) high copy numbers (mostly CN8) in most HER and moderate to high copy numbers in some SIM animals (mostly CN4) across all datasets. Datasets A and B also consisted of a very limited number of a composite breed or crossbreds with moderate copy numbers at the *KIT* locus CNVR, which is likely because those animals may have had SIM or HER animals in their pedigree. Surprisingly, in dataset B (Fig. 4b), there were 3 CHA with unexpectedly high CN genotypes and 1 HER with CN2 (30 of the 31 HER cattle with non-CN2 genotypes are depicted in the figure). Furthermore, 2 of those 3 CHA clustered with HER and the CN2 genotype HER clustered with CHA in the hierarchical clustering performed on the basis of genome-wide CNVR genotypes (Supplemental Fig. S7). In an earlier study [37], a PCA of dataset B samples based on their SNP genotypes revealed cross-clustering of the same 3 samples, which was attributed to potential issues with sourcing or handling of those samples. Similarly, in dataset C were an AAN and 2 LIM animals that showed CN8 genotype and clustered with the HER animals while 5 HER animals showed CN2 genotype but did not cluster with the rest of the HER animals in the hierarchical clustering performed on the basis of genome-wide CNVR genotypes (Supplemental Fig. S7). Manual inspection of the BAM files for those animals at the *KIT* locus CNVR indicated that the read coverages were in agreement with the genotypes predicted by cn.MOPS. Finally, as expected, the *KIT* locus CNVR was not detected in dataset D, which consisted exclusively of HOL animals. Another CNVR, ~15 kb in size (Chr6:71,810,000–71,825,000) and located within intron 1 of the *KIT* gene, has been reported to be associated with the piebald coat color [36]. In our analysis, the only CNVR that overlaps with this region and that shows amplification in the majority of HER and some SIM animals is an 11-kb CNVR at Chr6:71,808,000–71,819,000, identified only in dataset B. This CNVR was detected in 25 of the 31 HER (24 as CN3 and 1 as CN8) and 7 of the 34 SIM (all as CN3) individuals in dataset B. Thus, based on our results, the CNVR at Chr6:71,747,001–71,752,000 (upstream of the *KIT* gene) is more clearly associated with the piebald coat color.

### An interactive visual database of CNVRs in taurine cattle

**Figure 5:**
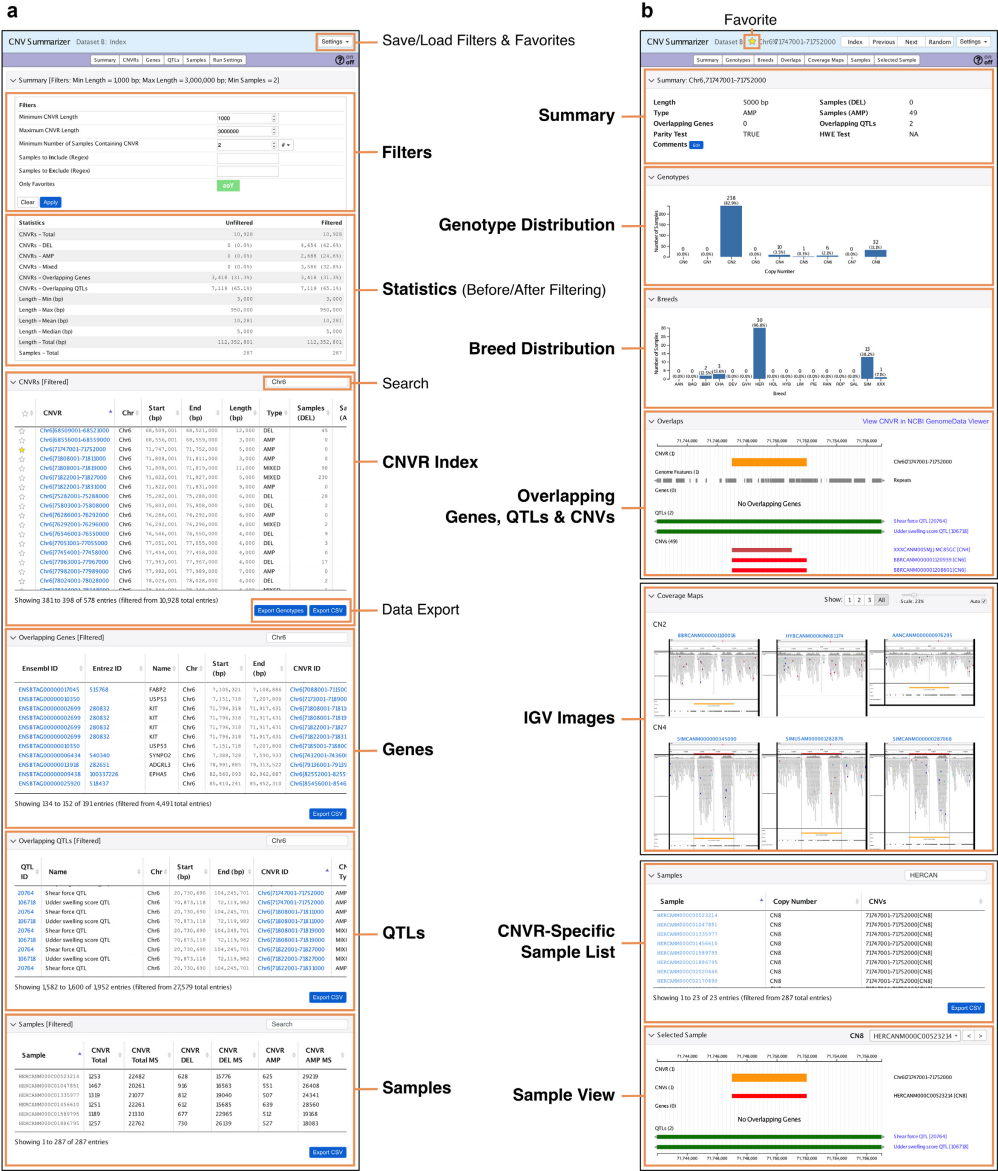
Key features of the functionality of the CNVR database. The database has an index view and a detailed view with an option to enable/disable the help function on the top right of each page. The index page (a) has a panel (Filters) that allows users to apply filters to the CNVRs such as CNVR length or the number of samples that must contain the CNVR and the ability to exclude/include specific samples based on regular expression matches. Another panel (Statistics) provides summary information on the CNVRs before and after applying the filters. The remaining panels on the index page allow users to search and sort on CNVRs, overlapping genes, and QTLs and/or samples to quickly find CNVRs associated with a particular gene/QTL. All or selected data can be exported as CSV files. CNVRs of interest can be noted as favorites; and comments can be added for individual CNVRs. All comments, filters, and/or favorites can be saved as a text file that can be reloaded later using the Settings button options on the top right of the page. Clicking on a CNVR provides a detailed view (b) with panels displaying basic statistics on the CNVR (Summary), a bar plot of the number of samples per CNV genotype (Genotype distribution), and another bar plot of the number of non-CN2 variants per breed (Breed distribution), graphical representation of the CNVR in genomic context (Overlapping genes, QTLs, and CNVs), sequence read coverage at the CNVR for up to 3 samples per genotype (IGV images), a table of all the samples indicating the CNV genotype (CNVR-specific sample list), and finally a sample view that provides, for the selected sample, a graphical representation of the CNVR and CNV in genomic context with overlapping genes and QTLs.

Studies of CNVs usually report CNVR positions but rarely the individual genotypes or the boundaries of constituent CNVs in individual samples, or supportive evidence at the level of individual CNVRs. Here we provide in-depth characterization of CNVRs and present the results in a comprehensive interactive database integrated with visualizations of sequence read alignments, CNV boundaries, and genome features that can be viewed in a modern web browser (for best results, use a recent version of Google Chrome or Mozilla Firefox). In doing so, our strategy better aligns with how we believe the CNVR data will be used: to investigate genome regions of interest for evidence of CNVs and to assess each CNVR with available supportive evidence. The key features of this database are represented in Fig. 5 using the *KIT* locus CNVR in dataset B as an example. An index page includes overall summary statistics on CNVRs, as well as custom filtering options for CNVRs and samples. Individual CNVRs are linked to detailed reports that provide a summary of the CNVR, graphs of CNVR genotypes per sample and breed, and visual representations of genome features (i.e., gaps, repeats, and segmental duplications), genes, quantitative trait loci (QTLs), and CNVs overlapping the CNVR. To determine genes that overlap with CNVRs, we also considered the 5-Mb regions flanking the gene boundaries as part of the gene. Additionally, a link to the NCBI Genome Data Viewer [38, 39] plots the CNVR region in the context of the latest annotations and genomics data available in NCBI for the UMD 3.1.1 bovine reference genome assembly. Using the viewer, the user can, for example, examine how RNA sequencing (RNA-Seq) data from a variety of tissues aligns with the region, which in turn can help to establish the presence or absence of transcribed regions in the vicinity of the CNVR. One of the most powerful and unique features of the CNVR database is the ability to view raw read alignments as images generated using the Integrative Genomics Viewer (IGV) [40, 41]. Images are provided for a random selection of up to 3 representative samples for each genotype, enabling assessment of the validity of the CNV genotypes and refinement of the CNV boundaries. Furthermore, for autosomal CNVRs, information is provided for tests on parity and Hardy-Weinberg equilibrium (HWE) of the CNVR genotypes. The majority of autosomal CNVRs (97% for datasets A–C; 91% for dataset D) passed the parity test (i.e., the combined frequencies of the heterozygote classes did not exceed that of the homozygote classes). Of the diallelic autosomal CNVRs that qualified for the HWE test per dataset (53–57% of the total for the 4 datasets; see Methods), the majority (63–88%) had genotype proportions that were in HWE (χ^2^ test *P*-value ≥ 10^−5^). In genome-wide association studies, departures from HWE based on genotypes of SNP markers are considered to indicate genotyping errors, batch effects, or population stratification, and therefore such markers are typically discarded. HWE results are provided as an additional characteristic/annotation of CNVRs, but we caution against filtering CNVRs on the basis of HWE because the test is limited to diallelic autosomal CNVRs and deviations from HWE could reflect inaccurate genotypes for an otherwise true CNVR of interest. The CNVR databases per dataset are available via the GigaDB data repository [42].

### Exploring the CNVR databases for variants of interest

We demonstrate the use of the CNVR database and the powerful interpretations possible through information on genomic features and visualization of read coverage at CNVRs. Following the creation of the CNVR database, and obtaining basic statistics and summaries of the CNVRs detected in each dataset, we analysed the database for CNVRs that span well-annotated genes and found several thousand CNVRs that partially or completely overlap genes in the 4 datasets. For example, with default filters for CNVR length (minimum 1 kb and maximum 3 Mb) and number of samples in which the CNVR is detected (n = 2), typing “cds del” in the search box of the “Overlapping Genes” panel for database A indicates 195 entries where a DEL type CNVR overlaps specifically with the coding sequence (CDS) of 1 or more genes (Supplemental Fig. S12a). Most of those CNVRs also overlap with other components of a gene such as the untranslated region or intron, or even extend further upstream or downstream of the gene (see column “Overlap Type” in the “Overlapping Genes” panel). Selecting the DEL-type CNVR Chr11:6,754,001–6,757,000 that overlaps with the interleukin 1 receptor type 2 gene (*IL1R2*) for a detailed view (Supplemental Fig. S12b) indicates that the CNVR passed the parity test but was not in HWE for genotype proportions. As discussed in the previous section, deviations from HWE should not be used as a criterion to filter CNVRs; instead visualization of the read coverage and other supporting information at the CNVR available through the CNVR database will help validate the predicted CNVs. The selected CNVR was detected in 5 samples, of which 4 were of CN0 and 1 of CN1 genotype (“Summary” and “Genotypes” panel). Furthermore, the “Overlaps” panel indicates that the CNV in each of the 5 samples overlaps completely with the penultimate exon and extends to the introns on either side of that exon of *IL1R2*, based on the Ensembl annotation of the gene. Viewing the affected region in the NCBI Genome Data Viewer (using the link provided in the report) corroborates the Ensembl gene model and provides additional support via RNA-Seq exon coverage data (Supplemental Fig. S12c). The CNVR was also detected in dataset B with a start position 1 kb upstream and in dataset C with an end position 1 kb downstream, compared to the coordinates of the CNVR in dataset A. The CNVR was not detected in dataset D, which consists only of HOLs, and the breed distribution of the CNVR in dataset B, the only other dataset with HOLs, supports the absence of this CNVR in HOLs (Supplemental Fig. S12d). The coverage maps (Supplemental Fig. S12e) reveal red-coloured reads at the boundaries of the CNVR, indicative of a larger than expected insert size, which is a hallmark of deletions. The coverage maps may also suggest potential genotyping errors by cn.MOPs. For example, in dataset C, the sample assigned CN1 appears, based on the absence of coverage over much of the CNVR, to be CN0. The genotyping may have gone wrong in this case because the end position of that CNVR was wrongly predicted to extend by >1 WL into a region of read coverage, which may have affected the calculation of average coverage across the CNVR during genotype assignment. The ability to view the read coverage maps at the CNVR also enables the refinement of the actual boundaries of the CNVR. CNV detection software that uses read-depth−based algorithms for CNV detection usually requires a detection window size defined according to the average depth of sequencing (1 kb window in the present analysis), and reports CNVR boundaries at the resolution of the window size. A potential improvement that could be made to the cn.MOPS algorithm is to programmatically resolve the CNVR boundaries to a higher resolution in cases where the read coverage at the CNVR allows it, thereby also improving genotype prediction. In the case of the CNVR within *IL1R2*, analysing the coverage maps helps to exclude the penultimate exon of that gene as being part of the CNVR because the map shows evidence of read coverage in all samples and datasets at that exon; therefore, the CNVR is actually limited to the intron. Thus, visualization helps to more precisely assess the potential impacts of the structural variants. It is important to note, however, that intronic CNVRs can affect phenotypes, for example as reported for the Pea-comb phenotype in chickens [43]. Another interesting gene where we detected separate intronic CNVRs covering 2 different introns of the gene across all datasets was calpastatin (*CAST*), wherein multiple SNPs associated with meat tenderness have been reported in beef cattle [44–49]. Here too, viewing the coverage map permits higher resolution determination of the CNVR boundaries (Supplemental Fig. S13a; the first of the 2 intronic CNVRs within *CAST*). Furthermore, the presence of coloured reads at the boundaries of the second intronic CNVR within *CAST*, even in samples of non-DEL genotype (Supplemental Fig. S13b), which initially seemed anomalous, could be explained on the basis of information available through the genomic features tracks, specifically assembly gaps of known (N) and unknown (U) sizes in the region of the CNVR boundaries. The coloured reads in such cases could be reads spanning the assembly gaps.

Finally, we provide an example where we looked for evidence of CNVRs at a region in the cattle genome that contains an interesting expanded family of lysozyme genes, which function in bacteria digestion in the abomasum [50]. A region of ~0.4 Mb on Chr5 between 44.35 and 44.75 kb encompasses several members of the lysozyme gene family located in tandem (Supplemental Fig. S14a). Exploring the CNVR database for dataset B, we identified 11 CNVRs of AMP or MIX type within the region of the lysozyme family of genes (Supplemental Fig. S14b). This example shows how the visualization can help better elucidate the diversity of component CNVs in a complex CNVR, with CNVs of differing genotypes occurring within close proximity to each other and sometimes within the same sample (Supplemental Fig. S14c), thus allowing for a better functional assessment.

Next, we provide an example of a breed-specific CNVR. While there were no CNVRs found fixed in all members of a breed, there were several that were only present in 2 or more members of a particular breed and absent in all other breeds. The number of such breed-specific CNVRs found in datasets A, B, and C (dataset D has only 1 breed and hence was excluded) varied from none in certain breeds to a few hundred in others (Supplemental Table S4) and were correlated with the number of samples per breed. Because our datasets consisted of only 1 dairy breed among the 17 breeds in total, the CNVRs found unique to HOL may indicate association with traits selected for in dairy cattle in general. For example, the CNVR Chr11:78,885,001–78,891,000 was found to be one of the most frequent breed-specific CNVRs in HOL, found in 11 of the 48 HOL in dataset B (all DEL) and 20 of the 32 HOL in dataset D (7 DEL, 13 AMP). Exploring this CNVR in the databases for datasets B (Supplemental Fig. S15a) and D (Supplemental Fig. S15b), the 2 datasets that consisted of HOL, revealed that the coverage maps from IGV support the CNVR genotypes and the red-coloured reads at the boundaries of the CN0 and CN1 genotype CNVRs further suggest a true deletion. The CNVR overlaps a known QTL for body weight (weaning) and the first exon of the Ensembl model for gene *MATN3*. Further exploration of the gene region via the link to the NCBI Genome Data Viewer (Supplemental Fig. S15c) indicates the following: the CNVR is upstream of the NCBI model of *MATN3* and there is no evidence of RNA-Seq exon coverage at the region of the first exon in the Ensembl model of *MATN3*. This absence of evidence of transcription could indicate either that the Ensembl model is not accurate or that the samples that contributed to the RNA-Seq data presented in the NCBI Genome Data Viewer were collected from a tissue or stage in life where the first exon of the gene was not transcribed. A previous study [51] identified a CNVR of almost identical coordinates (Chr11:78,884,928–78,891,111, “BovineCNV3591”) using Genome STRiP software [52] on WGS data from 22 Hanwoo (a Korean breed raised for beef) and 10 HOL breeds. The study reported that the CNVR had a higher deletion frequency in HOL compared to Hanwoo and indicated that the gene *MATN3* was also identified through their analysis of selective sweep signals based on fixation index (F_ST_) values for measures of population differentiation.

Visualization of the read coverages at CNVRs can also help identify potential false-positive calls by cn.MOPS, especially in regions of low sequencing coverage. In the case of the CNVRs depicted in Supplemental Fig. S16, the low coverage is clearly attributable to the numerous assembly gaps at the region. Setting a higher threshold for coverage and removing CNVRs detected within a certain distance from a known assembly gap may help resolve some of these cases at the expense of some loss of true-positive CNVRs. In the future, we plan to implement a filter that examines consistency of coverage across the window, allowing for deviations at the ends, to better identify and remove such cases.

The above examples, together with the example of the CNVR at the *KIT* gene locus described earlier (Figs 4 and 5), demonstrate the value of the CNVR databases created in this study. The data summaries, visualization of gene features, CNV genotypes, CNVR boundaries, and read coverages at CNVRs serve as powerful tools to ascertain the veracity and potential phenotype-altering mechanisms of CNVRs, as well as the prevalence of individual CNV genotypes among breeds and in the populations studied.

## Discussion

With the ever-reducing costs, WGS has become the method of choice for many applications involving CNV detection. Software to predict CNVs has also evolved, and methods that rely on multi-sample read-depth analyses, like cn.MOPS, have become popular owing to their superior ability to control for false-discovery rate [32]. Furthermore, a recent study on simulated data has reported read-depth−based approaches to perform relatively better than those based on paired-end and split-read analyses when analysing datasets composed of samples sequenced at varying levels of coverage [18, 21]. Using cn.MOPS, we analysed each of 4 WGS datasets, which together represent >500 bulls from 17 taurine cattle breeds. Besides CNV detection, cn.MOPS provides integer copy number genotypes to indicate the level of deletion or amplification at the predicted CNVs. We did not use the built-in function within cn.MOPS to construct CNVRs and assign CNVR genotypes because we found that this approach can produce very large CNVRs that obscure the underlying breakpoint diversity across samples and that have genotype assignments that are not always consistent with the majority genotype observed among the constituent CNVs. We therefore used a 50% pairwise reciprocal overlap criterion to construct CNVRs, as has been used in other studies [18, 21], and then assigned genotypes on the basis of a set of rules as described in the Methods section. The assigned CNVR genotypes indicated clear separation of breeds by hierarchical clustering and also confirmed previously reported differences in the amplification levels at the *KIT* locus CNVR between Simmental and Hereford breeds. In future work, individual CNVR genotypes could be used in association analyses aimed at investigating the relationship between copy number and phenotype. In addition, we provide detailed annotation including sequencing read coverage for each CNVR in multiple samples representing the different genotypes identified. All results are presented in a unique interactive visual database that enables the user to assess each CNVR based on sequence read alignments and to examine the boundaries of constituent CNVs in individual samples. Read coverage and alignments within and adjacent to a CNVR can aid in the determination of the breakpoints of constituent CNVs in individual samples because the resolution of the breakpoints reported by the cn.MOPS algorithm is limited to the choice of window size used for CNV detection. The visualization of genome features such as assembly gaps and repeats can highlight potential non−CNV-related coverage and alignment anomalies and thus can further be used in the assessment of predicted CNVs and their breakpoints. We believe that the way we present our results in the CNVR database better aligns with how this information will be used, i.e., to investigate genomic regions or genes of interest for evidence of CNVs; such information is not available at a genome-wide scale in any of the previously published reports on CNVRs in any species.

An important outcome from the present study was the necessity to address batch effects that could affect the reliability of CNVs predicted using algorithms that model read count variations across samples. The batch effects arise from genomic regions of imbalanced coverage across sequence datasets generated from different platforms and technologies. While the batch effects could potentially be controlled to an extent by including only those genomic regions that have adequate coverage across datasets, such an approach would have resulted in losing valuable information on CNVRs from individual datasets that had sufficient coverage at those regions. These observations guided our decision to analyse individual datasets separately.

One limitation of the present study was that some of the breeds had low sample representation; the PIE, RDP, and BBL breeds had <10 samples each while the BAQ, DEV, and SAL breeds had only 1 sample each. Therefore, the breadth of breed-specific CNVRs reported is not as complete for those breeds as are those for the more popular breeds with greater sample representation in the present study. Nevertheless, CNVRs in some of those breeds with smaller representation (e.g., DEV, SAL, BBL) have not been studied or reported earlier at a genome-wide scale, making this study amongst the first to do so in those breeds. Another limitation of the present study is that CNVRs shorter than 3,000 bp are not reported, which was the limit we set for the dataset-wise analyses based on the sequencing coverage of samples in the dataset with the lowest mean coverage.

## Conclusion

This study presents a comprehensive collection of CNVRs in taurine cattle, which can serve as a reference on the locations of CNVRs and their genotype frequencies in a broad range of cattle breeds. The visualizations and annotations included in the interactive databases greatly facilitate assessment of individual CNVRs and should aid the efforts to identify CNVRs that influence phenotype. We recommend that visualization of read coverage at predicted CNVRs be a standard protocol in studies reporting specific CNVRs of interest (e.g., near to a gene or genome region highlighted through some other research activities) among CNVRs identified on a genome-wide scale. Given the issue of false-positive calls inherent to any prediction algorithm and the impracticality of experimental validation for CNVRs at a genome-wide scale, read coverage visualization at CNVRs offers a powerful way not only to overcome those issues but also to refine the CNVR boundaries, among other advantages. Furthermore, we suggest integrating the NCBI Genome Data Viewer into analysis workflows as a way of assessing the NCBI and Ensembl gene models and their supporting evidence (e.g., RNA-Seq reads) when examining how CNVRs overlap with genome features.

## Methods

### Sequence data

The WGS datasets were generated in 4 different projects, which together comprised 553 samples representing 1 taurine dairy cattle breed and 16 taurine beef cattle breeds (Table 1 and Supplemental Table S1). The sequence data were generated following guidelines provided by the 1000 bull genomes project [52, 53]. Details on animal selection, sequence generation, and further analyses performed on datasets A and B have been published earlier [37, 54]. Briefly, DNA samples were extracted from commercial artificial insemination bull semen straws and sequenced using either the 5500xl SOLiD™ system (85 animals) or the HiSeq™ 2000 system (298 animals). Reads that passed standard quality-based filtering criteria were aligned to the UMD 3.1 bovine reference genome assembly [55] using the BWA-backtrack algorithm of Burrows-Wheeler Aligner (BWA, RRID:SCR_010910) [56] version 0.5.9. Local realignment of reads around indels was performed using the IndelRealigner tool of the Genome Analysis Toolkit (GATK) [57] version 2.4, and duplicate reads marked using the MarkDuplicates tool of the Picard toolkit version 1.54 [58]. Details on animal selection, sequence generation, and further analyses performed on datasets C and D were similar to those for the previous datasets except for using more recent versions of the following software: BWA version 0.7.15 for dataset C and version 0.7.12 for dataset D, both using BWA-MEM algorithm; GATK version 3.5; and Picard toolkit version 2.0.1.

### Identification of CNVs from sequence data

Detection of CNVs in the sequence data was performed using the Bioconductor [59] (version 3.6) package cn.MOPS (cn.mops, RRID:SCR_013036) [32] (version 1.24.0) of the R (version 3.4.3) statistical programming language [60] running on a CentOS 7 Linux server with default cn.MOPS parameters except the following: WL 1000 bp and rmdup enabled to count only 1 read for each unique combination of position, strand, and read width. CNVs were reported if 3 adjacent windows showed significant read-depth variations, thereby enabling the detection of CNVs of length ≥3,000 bp in increments of 1,000 bp.

### Constructing CNVRs from CNVs

In cn.MOPS, CNVRs are constructed from CNVs by merging overlapping and adjacent CNVs using the "reduce" function from the Bioconductor package “GenomicRanges.” An initial test run on dataset A using that approach resulted in abnormally large CNVRs. Hence, we followed a more conservative approach to merge CNVs to CNVRs similar to what was used in some previous studies [18, 21] in which CNVRs were constructed by merging only those CNVs across samples that satisfied a 50% pairwise reciprocal overlap criterion based on their genomic coordinates.

### Assigning genotypes to CNVRs

By default, cn.MOPS assigns CNVR genotypes for each sample based on the genotypes of the CNVs making up each CNVR. While the default approach worked well for the majority of cases, the selected genotype was not representative for 2.37% to 6.18% of the CNVRs across datasets where multiple discrete CNVs of differing genotypes occurred in certain individual samples. Such cases were observed more frequently for larger CNVRs. To assign CNVR genotypes, we used the genotype of the CNV type with the largest aggregate width amongst all CNV types making up the CNVR; in case of ties, we assigned the genotype that was closer to CN2. The corrected genotypes were used to perform genotype-based hierarchical clustering of samples (using the hclust function in R with the Spearman correlation–based distance measure and the ward.D2 agglomeration method). Another issue with genotype assignment to CNVRs is associated with the 50% reciprocal overlap criterion that allows creation of overlapping CNVRs. In general, a CN2 genotype is assigned to samples where a CNV is not detected in a particular CNVR; however, it is possible that the same sample may have a CNV of non-CN2 genotype detected on an overlapping CNVR. Therefore, we performed a CN2 correction as follows: for each test CNVR, the genotypes of samples for which cn.MOPS did not detect a CNV were changed from the default CN2 to CN_ in cases where a CNV was detected for that sample in another CNVR that overlapped with the test CNVR. The genotypes subsequently obtained were used for all summary calculations and plots created in the CNVR database.

### Annotation of CNVRs

The CNVRs were annotated for genes based on information obtained from Ensembl (Ensembl, RRID:SCR_002344) [61, 62] Release 88 (Bos_taurus.UMD3.1.88.gff3) and for cattle QTLs (99,652 QTLs) from Animal QTLdb (Animal QTLdb, RRID:SCR_001748) [63] Release 33 (Aug 26, 2017) [64]. Information on segmental duplications in bovines was retrieved from sheet 1 of additional file 3 (Table S3.1–7) of a previous study [65] whereas assembly gaps and repeats were obtained for Bos_taurus_UMD_3.1/bosTau6 (Nov. 2009) assembly University of California Santa Cruz (UCSC) genome table browser [66].

### Hardy-Weinberg equilibrium (HWE) test on CNVR genotypes

We performed Pearson's χ^2^ tests for goodness of fit of CNVR genotype proportions to HWE [67] at diallelic autosomal CNVRs with either a combination of CN0, CN1, and CN2 genotypes (considered as minor-allele homozygous, heterozygous, and reference homozygous) or CN2, CN3, and CN4 genotypes (considered as reference homozygous, heterozygous, and minor-allele homozygous), similar to a previous study [68]. The test was performed using the “HardyWeinberg” package [69] in R. Multi-allelic CNVR genotypes were not tested for HWE here because of the inability to determine what combination of alleles were responsible for a particular genotype. Furthermore, at all autosomal CNVRs, a parity test [70] was performed to test whether the number of individuals that have even CNVR genotypes (CN0, CN2, CN4, and CN8) exceed the number of individuals with odd CNVR genotypes (CN1, CN3, CN5, and CN7), an extension of the observation in SNP genotypes that, at HWE, the combined frequencies of the homozygote classes should exceed those of the heterozygote classes.

## Availability of supporting data and materials

Raw sequence data for datasets A and B are available from Sequence Read Archive (SRA) accessions SRP017441 and SRP044884. Aligned sequence data for 4 samples from dataset B (indicated in Supplemental Table S1) are available from SRA accession SRP017441 whereas those for the rest are available in the *GigaScience* GigaDB database [71]. Both raw and aligned sequence data for datasets C and D are available from SRA accessions SRP150844 and SRP153409 respectively. All supporting data and materials from this study including the CNVR databases per dataset are available in the *GigaScience* GigaDB database [42] or as Supplemental Files.

## Additional files

**Figure S1:** Sample-wise sequencing coverages per dataset.

**Figures S2–S5:** Proportions of the different CNV genotypes identified per sample (a), distributions of CNV genotype counts (b), proportions of DELs among CNVs (c), and total CNVs discovered (d) per dataset.

**Figures S6–S9:** Hierarchical clustering of samples based on the CNVR genotypes per dataset.

**Figure S10:** Chromosome-wise counts of total CNVRs and CNVRs per category (DEL, AMP, MIX) for datasets A (a), B (b), C (c), and D (d).

**Figure S11:** Phenograms representing the chromosomal locations of CNVRs belonging to the different categories for datasets A (a), B (b), C (c), and D (d).

**Figures S12–S16:** Specific examples to depict exploration of the CNVR databases for variants of interest.

**Table S1:** Detailed information on samples and sources of sequence data.

**Table S2:** List of CNVRs discovered in each dataset with the respective CNVR category assignments.

**Table S3:** Breed-wise summaries of CNVRs identified per dataset.

**Table S4:** Breed-specific CNVRs found in datasets A, B, and C.

## Abbreviations

AMP: amplification; BAM: Binary Alignment Map; BBR: Beef Booster; bp: base pairs; BWA: Burrows-Wheeler Aligner; CDS: coding sequence; CGH: comparative genomic hybridization; cn.MOPS: Copy Number estimation by a Mixture Of PoissonS; CNV: copy number variant; CNVR: CNV region; DEL: deletion type; GATK: Genome Analysis Toolkit; HWE: Hardy-Weinberg equilibrium; ICAR: International Committee for Animal Recording; IGV: Integrative Genomics Viewer; IL1R2: interleukin 1 receptor type 2; kb: kilobases; Mb: megabases; NCBI: National Center for Biotechnology Information; NGS: next-generation sequencing; PCA: principal component analysis; RNA-Seq: RNA sequencing; QC: quality control; QTL: quantitative trait locus; RDP: Rouge des Prés; SD: standard deviation; SNP: single-nucleotide polymorphism; SRA: Sequence Read Archive; UMD: University of Maryland; UCSC: University of California Santa Cruz; WGS: whole-genome seqencing; WL: window length.

## Competing interests

The authors declare that they have no competing interests.

## Funding

This research was supported by funding from Genome Canada, Genome Alberta, and Science Foundation Ireland (SFI) principal investigator award grant number 14/IA/2576 as well as a research grant from Science Foundation Ireland and the Department of Agriculture, Food and Marine on behalf of the Government of Ireland under the Grant 16/RC/3835 (VistaMilk).

## Author contributions

P.S. and C.F.B. designed the study. C.F.B., A.M.B., and D.P.B. oversaw sample selection, acquisition, and sequencing. A.K., K.K., A.M.B., and T.R.C. performed sequence analysis and/or CNV detection. J.R.G. developed the interactive CNV database. A.K. performed CNVR identification and downstream analyses steps and drafted the manuscript. All authors read, revised, and approved the manuscript.

## Supplementary Material

giz073_GIGA-D-18-00350_Original_Submission

giz073_GIGA-D-18-00350_Revision_1

giz073_GIGA-D-18-00350_Revision_2

giz073_Response_to_Reviewer_Comments_Original_Submission

giz073_Response_to_Reviewer_Comments_Revision_1

giz073_Reviewer_1_Report_Original_Submission Christine Couldrey -- 11/2/2018 Reviewed

giz073_Reviewer_2_Report_Original_Submission Aniek Bouwman -- 11/17/2018 Reviewed

giz073_Reviewer_2_Report_Revision_1 Aniek Bouwman -- 3/19/2019 Reviewed

giz073_Supplemental_Files
