# Supplementary material for: A large interactive visual database of copy number variants discovered in taurine cattle
Source: Gigascience. 2019 Jun 26;8(6):giz073. doi: 10.1093/gigascience/giz073 (PMC6593363; doi:10.1093/gigascience/giz073)
Supplement: giz073_GIGA-D-18-00350_Original_Submission [file giz073_giga-d-18-00350_original_submission.pdf]

GigaScience

A large interactive visual database of copy number variants discovered in taurine cattle

--Manuscript Draft--

Manuscript Number:	GIGA-D-18-00350
Full Title:	A large interactive visual database of copy number variants discovered in taurine cattle
Article Type:	Research
Funding Information:	Genome Canada — Dr Paul Stothard<br>Dr Christine F. Baes<br>Science Foundation Ireland (14/IA/2576) — Dr Donagh P. Berry<br>Genome Alberta — Dr Paul Stothard
Abstract:	Background<br><br>Copy number variants (CNVs) contribute to genetic diversity and phenotypic variation. We aimed to discover CNVs in taurine cattle using a large collection of whole-genome sequences and to provide an interactive database of the identified CNV regions (CNVRs) that includes visualizations of sequence read alignments, CNV boundaries and genome annotations.<br><br>Results<br><br>CNVs were identified in each of four whole genome sequencing datasets, which together represent over 500 bulls from 17 breeds, using a popular multi-sample read-depth based algorithm, cn.MOPS. Quality control and CNVR construction, performed dataset-wise to avoid batch effects, resulted in a total of 26,223 CNVRs covering 107.75 unique megabases (4.05%) of the bovine genome. Hierarchical clustering of samples by CNVR genotypes indicated clear separation by breeds. An interactive HTML database was created that allows data filtering options, provides graphical and tabular data summaries including Hardy-Weinberg equilibrium tests on genotype proportions, and displays genes and quantitative trait loci at each CNVR. Notably, the database provides sequence read alignments at each CNVR genotype and the boundaries of constituent CNVs in individual samples. Besides numerous novel discoveries, we corroborated the genotypes reported for a CNVR at the KIT locus known to be associated with the piebald coat colour phenotype in Hereford and some Simmental cattle.<br><br>Conclusions<br><br>We present the largest CNV collection yet in cattle in a novel interactive visual database that displays CNV boundaries, read depths and genome features for individual CNVRs, thus providing users with a powerful means to explore and scrutinize CNVRs of interest more thoroughly.
Corresponding Author:	Paul Stothard<br><br>CANADA
Corresponding Author Secondary Information:	
Corresponding Author's Institution:	
Corresponding Author's Secondary Institution:	
First Author:	Paul Stothard
First Author Secondary Information:	
Order of Authors:	Paul Stothard

Powered by Editorial Manager® and ProduXion Manager® from Aries Systems Corporation

	Arun Kommadath
	Jason R. Grant
	Kirill Krivushin
	Adrien Butty
	Christine F. Baes
	Tara R. Carthy
	Donagh P. Berry
**Order of Authors Secondary Information:**	
**Additional Information:**	
**Question**	**Response**
Are you submitting this manuscript to a special series or article collection?	No
**Experimental design and statistics**<br><br>Full details of the experimental design and statistical methods used should be given in the Methods section, as detailed in our Minimum Standards Reporting Checklist. Information essential to interpreting the data presented should be made available in the figure legends.<br><br>Have you included all the information requested in your manuscript?	Yes
**Resources**<br><br>A description of all resources used, including antibodies, cell lines, animals and software tools, with enough information to allow them to be uniquely identified, should be included in the Methods section. Authors are strongly encouraged to cite Research Resource Identifiers (RRIDs) for antibodies, model organisms and tools, where possible.<br><br>Have you included the information requested as detailed in our Minimum Standards Reporting Checklist?	Yes
**Availability of data and materials**	No

All datasets and code on which the conclusions of the paper rely must be either included in your submission or deposited in publicly available repositories (where available and ethically appropriate), referencing such data using a unique identifier in the references and in the "Availability of Data and Materials" section of your manuscript.<br><br>Have you have met the above requirement as detailed in our Minimum Standards Reporting Checklist?	
If not, please give reasons for any omissions below.<br><br>as follow-up to "**Availability of data and materials**<br><br>All datasets and code on which the conclusions of the paper rely must be either included in your submission or deposited in publicly available repositories (where available and ethically appropriate), referencing such data using a unique identifier in the references and in the "Availability of Data and Materials" section of your manuscript.<br><br>Have you have met the above requirement as detailed in our Minimum Standards Reporting Checklist?<br><br>"	Data to be made available through GigaDB require a manuscript ID first, so we will submit them soon after we receive the ID.

Manuscript Click here to download Manuscript CNVR_manuscript.docx

A large interactive visual database of copy number variants discovered in taurine cattle

Arun Kommadath[1], Jason R. Grant[1], Kirill Krivushin[1], Adrien Butty[2], Christine F. Baes[2], Tara R. Carthy[3], Donagh P. Berry[3] and Paul Stothard[1*]

[1] Department of Agricultural, Food and Nutritional Science (AFNS), 1400 College Plaza, 8215 - 112 Street, Edmonton, Alberta, Canada T6G 2C8

[2] Centre for Genetic Improvement of Livestock, Department of Animal Biosciences, University of Guelph, Guelph, Canada

[3] Teagasc, Animal & Grassland Research and Innovation Centre, Moorepark, Fermoy, Ireland

[*] Corresponding author

Email addresses:

Arun Kommadath: kommadat@ualberta.ca

Jason R. Grant: jason.grant@ualberta.ca

Kirill Krivushin: krivushi@ualberta.ca

Adrien Butty: buttya@uoguelph.ca

Christine F. Baes: cbaes@uoguelph.ca

Tara R. Carthy: tara.carthy@teagasc.ie

Donagh P. Berry: donagh.berry@teagasc.ie

Paul Stothard: stothard@ualberta.ca

Keywords: CNV, structural variants, cattle, dairy, beef, whole-genome sequencing, database, sequence visualization

ABSTRACT

Background

Copy number variants (CNVs) contribute to genetic diversity and phenotypic variation. We aimed to discover CNVs in taurine cattle using a large collection of whole-genome sequences and to provide an interactive database of the identified CNV regions (CNVRs) that includes visualizations of sequence read alignments, CNV boundaries and genome annotations.

Results

CNVs were identified in each of four whole genome sequencing datasets, which together represent over 500 bulls from 17 breeds, using a popular multi-sample read-depth based algorithm, cn.MOPS. Quality control and CNVR construction, performed dataset-wise to avoid batch effects, resulted in a total of 26,223 CNVRs covering 107.75 unique megabases (4.05%) of the bovine genome. Hierarchical clustering of samples by CNVR genotypes indicated clear separation by breeds. An interactive HTML database was created that allows data filtering options, provides graphical and tabular data summaries including Hardy-Weinberg equilibrium tests on genotype proportions, and displays genes and quantitative trait loci at each CNVR. Notably, the database provides sequence read alignments at each CNVR genotype and the boundaries of constituent CNVs in individual samples. Besides numerous novel discoveries, we corroborated the genotypes reported for a CNVR at the *KIT* locus known to be associated with the piebald coat colour phenotype in Hereford and some Simmental cattle.

Conclusions

We present the largest CNV collection yet in cattle in a novel interactive visual database that displays CNV boundaries, read depths and genome features for individual CNVRs, thus providing users with a powerful means to explore and scrutinize CNVRs of interest more thoroughly.

INTRODUCTION

Structural variants (SVs), originally defined to include insertions, deletions and inversions greater than 1 kilobase (Kb) in size [1], now encompass events as small as 50 base pairs (bp) [2]; this change in definition is likely due, in part, to developments in sequencing technology that greatly improved the resolution of discovery achievable. Copy number variants (CNVs) are a class of unbalanced structural variants characterized by changes to the number of base pairs in the genome and manifested as gains or losses of regions of genomic sequence between individuals of a species; CNVs therefore contribute to genetic diversity. Several examples have been reported of CNVs associated with normal variation, disease, evolution and adaptive traits in human, animal and plant species [3–7]. With next-generation sequencing (NGS) technology becoming more cost-effective, traditional methods for CNV discovery that involved hybridization-based microarray approaches like array comparative genomic hybridization (array CGH) and SNP microarrays are now being replaced by powerful sequencing-based computational approaches.

Studies on CNV discovery and characterization have been performed on several farm animal species [8–14] with the ultimate objective of using variants that are associated with traits of economic importance in genetic improvement programs. In cattle, several studies [15–29] have been conducted, in both taurine and indicine breeds, using a variety of algorithms to identify thousands of CNVs. While attempts have been made to provide overall assessments on the reliability of CNVRs reported in some of those studies using approaches like parent-offspring trios [9], PCR [8] or a combination of *in silico* and experimental techniques [29], the majority have been limited to providing the CNVR boundaries alone. Assessing the potential impact of CNVRs at individual and population levels becomes difficult in the absence of genotypes and boundaries of CNVs constituting CNVRs in individual samples. A recent study [30] has proposed the use of BAM confirmation (i.e. visually examining read depth and read pairing characteristics) as a strategy to assess the accuracy of predicted CNVRs. This approach was

then applied to a limited number of CNVs selected based on overlap with certain human disease-associated genes [30]. However, to the best of our knowledge, there are no current studies that provide such supportive evidence to assess predicted CNVRs of interest on a genome-wide scale.

The objectives of the present study were to identify and characterize genome-wide CNVRs among popular taurine cattle (*Bos taurus*) breeds and to present the results in a comprehensive interactive database of CNVRs and copy number genotypes, integrated with visualizations of sequence read alignments and genome features. Briefly, CNVs were identified in each of four whole genome sequencing (WGS) datasets, which together represent 553 bulls and 17 different breeds (one dairy and 16 beef breeds). We used cn.MOPS [31], a popular CNV detection software that employs a multi-sample read-depth based algorithm to estimate copy number genotypes per sample. Custom software was then used to convert the results for each dataset into an interactive visual database, a first of its kind for genome-wide CNVR data in any species. The databases, which can be downloaded and then opened using a modern web browser, give users the ability to assess each CNVR with supportive evidence and multiple levels of genome annotation. Further advantages of this format include, for example, the ability to adjust filtering criteria, compare CNV boundaries and genotypes across samples, and search for affected genes or regions of interest.

RESULTS

Adverse influence of batch effects on CNV discovery from combined datasets

We obtained WGS data on a total of 553 bulls from four different sources; all were paired-end sequenced but differed in the sequencing platform used as well as the coverage, read length, sample size and breed representation (Table 1). Detailed information on samples and breed code translations are provided in Supplemental Table S1 online. Dataset A was generated using the SOLiD platform and

had lower read length and mean coverage (Supplemental Figure S1) than datasets generated using the Illumina platform.

Table 1. Sequencing and sample characteristics per dataset

Dataset (year sequenced)	Platform (read length)	Coverage mean (SD)	Total samples	Breed codes * (Number of samples)
A (2012-13)	SOLiD 5500xl (75x35 bp)	7X (4.6)	85	SIM(30), LIM(28), CHA(16), BBR(8), GVH(3)
B (2013-14)	Illumina HiSeq 2000 (100 bp)	11.6X (3.3)	298	HOL(48), AAN(47), SIM(35), HER(33), GVH(28), RAN(26), CHA(25), BBR(16), XXX(14), PIE(7), RDP(7), LIM(6), HYB(3), BAQ(1), DEV(1), SAL(1)
C (2016)	Illumina HiSeq X (150 bp)	10.3X (2.6)	138	CHA(42), LIM(30), SIM(27), AAN(15), HER(15), BBL(9)
D (2017)	Illumina HiSeq X Ten (150 bp)	37.9X (3.6)	32	HOL(32)

* The breed codes used for purebred cattle follow the guidelines provided by the International Committee for Animal Recording (ICAR) for identification of semen straws for international trade. In addition, XXX represents crossbred cattle and HYB represents composite breeds other than Beef Booster (BBR).

Using aligned sequence data from all bulls simultaneously as input into cn.MOPS, we assessed counts of reads aligned to each non-overlapping window across the genome. The window length (WL) was chosen such that each segment comprised on average 100 reads, as is recommended in cn.MOPS

documentation. A WL of 1000 bp satisfied this criterion for datasets A-C. For uniformity, we chose to keep the same WL for dataset D, despite the fact that it had substantially greater sequencing coverage (Table 1) would have allowed for a lower WL. The CNV discovery algorithm implemented in cn.MOPS derives its power from modelling read count variations across samples, therefore read count normalization was performed as a prerequisite. A principal component analysis (PCA) on the normalised read counts per segment across samples revealed clear separation amongst datasets, which was indicative of uncorrected batch effects (Figure 1a). Proceeding with CNV discovery and genotype characterization using those read counts (after excluding the four PCA outliers) revealed considerable differences in the distribution of CNV genotypes per dataset (Figure 1b). The genotype distributions were skewed towards deletion type (DEL) CNVs in datasets A and B (datasets with comparatively lower read lengths) as opposed to datasets C and D where the distributions were skewed towards amplification (AMP) type CNVs. These aberrations may arise from the presence of more regions of limited or no coverage in datasets A and B, which have triggered false DEL type CNV genotype calls when compared across corresponding regions in other datasets with adequate coverage due to longer read length or advances in sequencing technology. Together, these results indicated the necessity to analyse distinct datasets individually with additional dataset-specific filters applied to identify and remove outlier samples.

Distributions of CNV genotypes were more consistent across datasets that were analysed individually

To avoid the adverse influence of batch effects on CNV discovery with cn.MOPS when combining datasets with genomic regions of imbalanced coverage, we analyzed each dataset individually. Using cn.MOPS, CNVs were identified after first excluding the four PCA outliers (3 in dataset A and 1 in dataset B; see Figure 1b) and three samples within dataset A that were of substantially higher coverage than the others within that dataset (Supplemental Figure S1). Contrary to what was observed when datasets were combined, the proportions of DELs among CNVs were quite consistent among datasets

analyzed individually (Figure 2), with mean proportion of DELs ranging between 0.55 (SD 0.08) for dataset D and 0.61 (SD 0.09) for dataset B. Additional quality control (QC) steps were applied to identify problematic samples, defined as those that showed marked deviations (1.5 times the interquartile range away from the first and third quartiles) in the proportion of DELs or total CNVs discovered within each dataset. The total number of problematic samples identified were 7, 10, 7 and 3 respectively for datasets A to D. For dataset A, most of the problematic samples identified were amongst the lowest coverage samples (coverage below 5X) while for the other datasets with higher coverage, such a trend was not clearly evident. Plots per dataset that indicate the proportions of the different CNV genotypes identified per sample, distributions of CNV genotype counts, proportions of DELs among CNVs and total CNVs discovered are provided in Supplemental Figures S2-S5 online with problematic samples labelled. All CNVs called within problematic samples were removed which improved the consistency among datasets, with means of the proportions of DELs ranging between 0.57 (SD 0.06) for dataset C and 0.60 (SD 0.07) for dataset B. The CNVs, from the 519 samples that remained after QC, were used to construct CNVRs per dataset based on a 50% reciprocal overlap criterion, consistent with the procedure used elsewhere [26,29]. Finally, refined sets of CNVRs were obtained after filtering out CNVRs observed in only one sample per dataset. Based on the genotypes of constituent CNVs, the CNVRs were categorised as DEL (CN0/CN1), AMP (CN3+) or mixed (MIX) type (one or more of CN0/CN1 and CN3+). Dataset-wise hierarchical clustering of samples based on the CNVR genotypes (representative genotype of CNVs comprising each CNVR; see Methods) revealed clear clustering by breeds (Supplemental Figures S6-S9 online) as expected.

A list of CNVRs discovered in each dataset with the respective CNVR category assignments is provided in Supplemental Table S2 online. The list consists of a total of 26223 unique CNVRs, counting those with identical genomic coordinates across datasets only once. The dataset-wise counts of CNVs and CNVRs and the non-redundant genome length covered by CNVRs (Table 2) were all proportional to the sample sizes of the individual datasets. These relationships were as expected and were also

observed at the breed level (breed-wise summaries of CNVRs are provided in Supplemental Table S3 online). Notably, dataset B had the greatest number of CNVRs in total, which may be attributed to its larger sample size and diversity of breeds, which included purebreds, crossbreds and composites. Conversely, dataset D had the lowest genome coverage by CNVRs, which may be attributed to the fact that it comprised only one breed and thus less genomic variability compared to the other datasets with multiple breeds. These differences amongst datasets were also reflected in the chromosome-wise counts of total CNVRs of each category where datasets of larger sample size and breed diversity revealed higher proportions of MIX category CNVRs (Supplemental Figure S10 a-d online; lower panel). Chromosomes 12, 15, 14 and 29 had comparatively higher density of CNVRs (CNVR counts per megabase (Mb) over the third quartile in all datasets) than others whereas chromosomes 2, 11, 13, 24 and 22 were amongst the least dense (Supplemental Figure S10 a-d online; upper panel). Phenograms representing the chromosomal locations of CNVRs belonging to the different categories indicate distinct patterns broadly conserved across datasets (Supplemental Figure S11 a-d online).

Table 2. Dataset-wise summary of CNVs and CNVRs

Dataset	Number (No.)				Size (Kb) of largest CNVR	Non-redundant size of genome (Mb) covered by CNVRs (%)
	Samples post-QC (No. pre-QC)	CNVs post-QC (No. pre-QC)	CNVRs post-QC (No. pre-QC)	CNVRs per category (No. of DELs; AMPs; MIX)		
A	72 (79)	35531 (41673)	6864 (11625)	2012; 2660; 2192	378	53.8543 (2.02)
B	287 (297)	103040 (117104)	10928 (19139)	2687; 4646; 3595	950	92.48615 (3.48)
C	131	54797	8056	2522; 2793;	501	65.90313

	(138)	(61050)	(12351)	2741		(2.48)
D	29<br>(32)	17790<br>(20107)	5749<br>(8988)	1911; 1845;<br>1993	580	44.47765<br>(1.67)
Overall summary	519<br>(546)	157862<br>(182355)	26223<br>(44836)	9974; 8302;<br>9115	950	107.7467<br>(4.05)

* For the overall summary, the non-redundant size of genome covered was obtained by merging overlapping or adjacent CNVRs across datasets whereas the numbers of CNVs, CNVRs and CNVRs per category were obtained by counting CNVRs with unique genomic coordinates.

Overlaps between CNVRs identified in the four datasets were low when compared to those reported in previous studies but high between the datasets themselves

Previous studies that compared CNVRs discovered across studies reported low percentages of overlap which is attributable to the numerous differences among studies in sample size and characteristics, sequencing platform and technology and CNV detection algorithm, among others. In cattle, the percentages of overlap among CNVRs discovered across multiple studies were generally below 40% [3,32], with overlapping CNVRs defined as those that share at least one base position. In agreement, the percentages of overlap between the CNVRs detected in 4 datasets of the present study and those detected in previous studies were generally low, ranging between 22 and 35% on average (Table 3). A merged list of CNVRs from the 4 datasets consisted of 9482 CNVRs (mean CNVR size 11.363 Kb; largest CNVR size 3.152 Mb), of which, on average, 37% overlapped with the CNVRs identified in previous studies (Table 3; ABCD). The list was generated by merging overlapping or adjacent CNVRs across datasets as was performed earlier to determine the overall non-redundant size of genome covered by CNVRs (see Table 2). Surprisingly, in another comparison limited to the four datasets, between 70 and 92% of the CNVRs detected in the smaller datasets (A, C and D) overlapped with CNVRs in dataset B, the dataset with the largest sample size and breed representation (Figure 3).

Despite the differences amongst the four datasets, the high degree of overlap between CVNRs identified could point to the choice of the CNV detection algorithm being the factor that contributes most to variability in CNVs discovered across studies.

Table 3. Overlaps between CNVRs identified in this study with those from previous published reports

Study	Platform	Nr. chr.	Nr. breeds, samples and CNVRs	% overlap with CNVRs identified in this study				
				A	B	C	D	ABCD
Fadista et al. [15]	CGH-based	29+X	4; 20; 266	12	16.9	13.9	11.3	18
Liu et al. [16]		29+X	17; 90; 223	65.5	78	71.7	57.4	78.9
Hou et al. [17]	SNP-based (50K chip)	29	21; 521; 743	35.8	48	35.1	30.6	51.1
Bae et al. [18] *		29	1; 265; 224	16.5	29	14.3	10.3	33.9
Hou et al. [19]		29	1; 472; 500	21	31.8	21	16.6	35.6
Jiang et al. [20]		22	1; 2047; 64	31.2	48.4	25	21.9	48.4
Hou et al. [21]	SNP-based (HD chip)	29	27; 674; 3438	19.4	28.4	20.5	15.4	33
Wu et al. [22]		29+X	1; 792; 263	38.8	49.8	39.2	29.3	54.4
Bickhart et al. [23]	Whole genome sequencing (WGS)	29	3; 5; 763	10.6	14.4	11.1	9.3	16
Zhan et al. [24]		29	1; 1; 419	8.1	11.5	8.4	9.5	13.8
Stothard et al. [25]		26	2; 2; 634	12.3	15.1	13.2	11.7	16.2
Keel et al. [26]		29+X	7; 154; 1341	60.8	66.4	64	56.3	67.2
Chen et al. [27]		29+X	2; 316; 16325	6.7	10.7	8.1	5.5	12.2
			Mean % overlap	26.05	34.49	26.58	21.93	36.82

Nr. of breeds, samples and CNVRs identified in this study	5; 72; 6864	16; 287; 10928	6; 131; 8056	1; 29; 5749	17; 517; 9482

***** For studies that used the BTAU 4.0 assembly for mapping, we used the UCSC liftOver tool (https://genome.ucsc.edu/cgi-bin/hgLiftOver) to convert the genomic coordinates of the CNVRs to UMD 3.1.

Identification and genotyping of the well-characterised *KIT* locus CNV in our datasets

A CNVR at Chr6:71747001-71752000, found approximately 45 Kb upstream of the *KIT* gene (Chr6:71796318-71917431) has been reported to be associated with the piebald coat colour phenotype in HER and some SIM cattle [33–35], but not the dorsal spotting on SIM and HOL cattle or the white patterning on Rouge des Prés [35] (RDP; formerly called Maine-anjou). As one of the few breed-associated cattle CNVs with available genotypes described in the literature we looked at whether our analysis produced consistent breed specificity and genotypes at the *KIT* locus CNVR. Overall, we found (Figure 4) high copy numbers (mostly CN8) in most HER and moderate to high copy numbers in some SIM animals (mostly CN4) across all datasets. Datasets A and B also consisted of a very limited number of a composite breed or crossbreds with moderate copy numbers at the *KIT* locus CNVR, which is likely as those animals may have had SIM or HER animals in their pedigree. Surprisingly, in dataset B (Figure 4b), were 3 CHA with unexpectedly high CN genotypes and 1 HER with CN2 (30 of the 31 HER cattle with non-CN2 genotypes are depicted in the figure). Further, 2 of those 3 CHA clustered with HER and the CN2 genotype HER clustered with CHA in the hierarchical clustering performed based on genome-wide CNVR genotypes (Supplemental Figure S7 online). In an earlier study [36], a PCA of dataset B samples based on their SNP genotypes revealed cross-clustering of the same 3 samples, which was attributed to potential issues with sourcing or handling of those samples. Similarly, in dataset C were an AAN and 2 LIM animals that showed CN8 genotype and clustered with the HER animals while 5 HER animals showed CN2 genotype and did not cluster with the rest of the HER animals in the hierarchical clustering performed based on genome-wide CNVR genotypes

(Supplemental Figure S7 online). Manual inspection of the BAM files for those animals at the *KIT* locus CNVR indicated that the read coverages were in agreement with the genotypes predicted by cn.MOPS. Finally, as expected, the *KIT* locus CNVR was not detected in dataset D which consisted exclusively of HOL animals.

An interactive visual database of CNVRs in taurine cattle

Studies of CNVs usually report CNVR positions but rarely the individual genotypes or the boundaries of constituent CNVs in individual samples, or supportive evidence at the level of individual CNVRs. Here we provide in-depth characterization of CNVRs and present the results in a comprehensive interactive database integrated with visualizations of sequence read alignments, CNV boundaries, and genome features that can be viewed in a modern web browser (for best results, use latest versions of Google Chrome or Mozilla Firefox). In doing so, our strategy better aligns with how we believe the CNVR data will be used: to investigate genome regions of interest for evidence of CNVs and to assess each CNVR with available supportive evidence. The key features of this database are represented in Figure 5 using the *KIT* locus CNVR in dataset B as an example. An index page includes overall summary statistics on CNVRs, as well as custom filtering options for CNVRs and samples. Individual CNVRs are linked to detailed reports that provide a summary of the CNVR, graphs of CNVR genotypes per sample and breed and visual representations of genome features (i.e., gaps, repeats and segmental duplications), genes, QTLs, and CNVs overlapping the CNVR. To determine genes that overlap CNVRs, we also considered the 5 Mb regions flanking the gene boundaries as part of the gene. Additionally, a link to the NCBI Genome Data Viewer (www.ncbi.nlm.nih.gov/genome/gdv/) [37] plots the CNVR region in the context of the latest annotations and genomics data available in NCBI. Using the viewer, the user can, for example, examine how RNA-Seq data from a variety of tissues aligns with the region, which in turn can help to establish the presence or absence of transcribed regions in the vicinity of the CNVR. One of the most powerful and unique features of the CNVR database is the ability to view raw read alignments as images generated using the Integrative Genomics Viewer (IGV) [38,39]. Images are provided for a

random selection of up to three representative samples for each genotype, enabling assessment of the validity of the CNV genotypes and refinement of the CNV boundaries. Furthermore, for autosomal CNVRs, information is provided for tests on parity and Hardy-Weinberg equilibrium (HWE) of the CNVR genotypes. The majority of autosomal CNVRs (97% for datasets A-C; 91% for dataset D) passed the parity test (i.e. the combined frequencies of the heterozygote classes did not exceed that of the homozygote classes). Of the CNVRs that qualified for the HWE test per dataset (53-57% of the total for the 4 datasets; see Methods), the majority (63-88%) had genotype proportions that were in HWE (Chi-squared test p-value $\geq 10^{-5}$). In genome-wide association studies, departures from HWE based on genotypes of SNP markers are considered to indicate genotyping errors, batch effects or population stratification and therefore such markers are typically discarded. We recommend that CNVRs with genotypes that deviate from HWE be thoroughly verified based on read coverage and other supporting evidence available for that CNVR. The CNVR databases per dataset are available via the GigaDB data repository (http://gigadb.org/).

Exploring the CNVR databases for variants of interest

We demonstrate the use of the CNVR database and the powerful interpretations possible through information on genomic features and visualization of read coverage at CNVRs. Following the creation of the CNVR database and obtaining basic statistics and summaries of the CNVRs detected in each dataset, we analysed the database for CNVRs that span well-annotated genes and found several thousand CNVRs that partially or completely overlap genes in the four datasets. For example, with default filters for CNVR length (minimum 1 Kb and maximum 3 Mb) and number of samples in which the CNVR is detected (n=2), typing ‘cds del’ in the search box of the ‘Overlapping Genes’ panel for database A indicates 195 entries where a DEL type CNVR overlaps specifically with the coding sequence (CDS) of one or more genes (Supplemental Figure S12 a). Most of those CNVRs also overlap with other components of a gene like the untranslated region (UTR) or intron, or even extend further upstream or downstream of the gene (see column ‘Overlap Type’ in the ‘Overlapping Genes’

panel). Selecting the DEL-type CNVR Chr11:6754001-6757000 that overlaps with the interleukin 1 receptor type 2 gene (*IL1R2*) for a detailed view (Supplemental Figure S12 b) indicates that the CNVR passed the parity test but was not in HWE for genotype proportions. As discussed in the previous section, deviations from HWE could be due to a number of reasons, and visualization of the read coverages and other supporting information at the CNVR available through the CNVR database will help validate the predicted CNVs. The selected CNVR was detected in five samples, of which four were of CN0 and one of CN1 genotype (‘Summary’ and ‘Genotypes’ panel). Further, the ‘Overlaps’ panel indicates that the CNV in each of the 5 samples overlaps completely with the penultimate exon and extends to the introns on either side of that exon of *IL1R2*, based on the Ensembl annotation of the gene. Viewing the affected region in the NCBI Genome Data Viewer (using the link provided in the report) corroborates the Ensembl gene model and provides additional support via RNA-Seq exon coverage data (Supplemental Figure S12 c). The CNVR was also detected in dataset B with a start position 1 Kb upstream and in dataset C with an end position 1 Kb downstream, compared to the coordinates of the CNVR in dataset A. The CNVR was not detected in dataset D which consists only of HOLs, and the breed distribution of the CNVR in dataset B, the only other dataset with HOLs, supports the absence of this CNVR in HOLs (Supplemental Figure S12 d). The coverage maps (Supplemental Figure S12 e) reveal red-coloured reads at the boundaries of the CNVR, indicative of a larger than expected insert size, which is a hallmark for deletions. The coverage maps may also suggest potential genotyping errors by cn.MOPs. For example, in dataset C, the sample assigned CN1 appears, based on the absence of coverage over much of the CNVR, to be CN0. The genotyping may have gone wrong in this case because the end position of that CNVR was wrongly predicted to extend by over one window length into a region of read coverage, which may have affected the calculation of average coverage across the CNVR while assigning the genotype. The ability to view the read coverage maps at the CNVR also allows for refining the actual boundaries on the CNVR. CNV detection software that utilize read-depth based algorithms for CNV detection usually require a detection window size defined according to the average depth of sequencing (1 Kb window in the current analysis), and report CNVR

boundaries at the resolution of the window size. A potential improvement that could be made to the cn.MOPS algorithm is to programmatically resolve the CNVR boundaries to a higher resolution in cases where the read coverage at the CNVR allows it, thereby also improving genotype prediction. . In the case of the CNVR within *IL1R2*, analysing the coverage maps helps to exclude the penultimate exon of that gene as being part of the CNVR, as the map shows evidence of read coverage in all samples and datasets at that exon; therefore, the CNVR is actually limited to the intron. Thus, visualization helps to more precisely assess the potential impacts of the structural variants. It is important to note, however, that intronic CNVRs can have effects on phenotype, for example as reported for the *Pea-comb* phenotype in chickens [40]. Another interesting gene where we detected separate intronic CNVRs covering two different introns of the gene across all datasets was calpastatin (*CAST*), wherein multiple SNPs associated with meat tenderness have been reported in beef cattle [41–46]. Here too, viewing the coverage map permits higher resolution determination of the CNVR boundaries (Supplemental Figure S13 a; the first of the 2 intronic CNVRs within *CAST*). Further, the presence of coloured reads at the boundaries of the second intronic CNVR within *CAST*, even in samples of non-DEL genotype (Supplemental Figure S13 b), which initially appeared anomalous, could be explained based on information available through the genomic features tracks, specifically assembly gaps of known (N) and unknown (U) sizes in the region of the CNVR boundaries. The colored reads in such cases could be reads spanning the assembly gaps.

Next, we provide an example where we looked for evidence of CNVRs at a region in the cattle genome that contains an interesting expanded family of lysozyme genes, which function in bacteria digestion in the abomasum [47]. A region of approximately 0.4 Mb on Chr5 between 44.35 and 44.75 Kb encompasses several members of the lysozyme gene family located in tandem (Supplemental Figure S14 a). Exploring the CNVR database for dataset B, we identified 11 CNVRs of AMP or MIX type within the region of the lysozyme family of genes (Supplemental Figure S14 b). This example shows how the visualization can help better appreciate the diversity of component CNVs in a complex CNVR, with

CNVs of differing genotypes occurring within close proximity to each other and sometimes within the same sample (Supplemental Figure S14 c), thus allowing for a better functional assessment.

Next, we provide an example of a breed-specific CNVR. While there were no CNVRs found fixed in all members of a breed, there were several that were only present in 2 or more members of a particular breed and absent in all other breeds. The number of such breed-specific CNVRs found in datasets A, B and C (dataset D has only one breed and hence excluded) varied from none in certain breeds to a few hundred in others (Supplemental Table S4) and were correlated with the number of samples per breed. Since our datasets consisted of only one dairy breed among the 17 breeds in total, the CNVRs found unique to HOL may indicate association with traits selected for in dairy cattle in general. For example, the CNVR, Chr11:78885001-78891000, was found to be one of the most frequent breed-specific CNVRs in HOL, found in 11 of the 48 HOL in dataset B (all DEL) and 20 of the 32 HOL in dataset D (7 DEL, 13 AMP). Exploring this CNVR in the databases for datasets B (Supplemental Figure S15 a) and D (Supplemental Figure S15 b), the two datasets that consisted of HOL, indicated that the coverage maps from IGV support the CNVR genotypes and the red-coloured reads at the boundaries of the CN0 and CN1 genotype CNVRs further suggest a true deletion. The CNVR overlaps a known QTL for body weight (weaning) and the first exon of the Ensembl model for gene *MATN3*. Further exploration of the gene region via the link to the NCBI Genome Data Viewer (Supplemental Figure S15 c) indicates the following: the CNVR is upstream of the NCBI model of *MATN3* and there is no evidence of RNA-Seq exon coverage at the region of the first exon in the Ensembl model of *MATN3*. This absence of evidence of transcription could indicate that either the Ensembl model is not accurate or that the samples that contributed to the RNA-Seq data presented in the NCBI Genome Data Viewer were collected from a tissue or stage in life where the first exon of the gene was not transcribed. A previous study [48] identified a CNVR of almost identical coordinates (Chr11:78884928-78891111, “BovineCNV3591”) using Genome STRiP software [49] on WGS data from 22 Hanwoo (a Korean breed raised for beef) and 10 HOL breeds. The study reported that the CNVR had a higher deletion frequency

in HOL compared to Hanwoo and indicated that the gene *MATN3* was also identified through their analysis of selective sweep signals based on fixation index (F_{ST}) values for measures of population differentiation.

Visualization of the read coverages at CNVRs can also help identify potential false positive calls by cn.MOPS especially in regions of low sequencing coverage. In the case of the CNVRs depicted in Supplemental Figure S16, the low coverage is clearly attributable to the numerous assembly gaps at the region. Setting a higher threshold for coverage and removing CNVRs detected within a certain distance from a known assembly gap may help resolve some of these cases at the expense of some loss of true positive CNVRs. In future, we plan to implement a filter that looks at consistency of coverage across the window, allowing for deviations at the ends, to better identify and remove such cases.

The above examples, together with the example of the CNVR at the *KIT* gene locus described earlier (Figure 4 and 5), demonstrate the value of the CNVR databases created through this study. The data summaries, visualization of gene features, CNV genotypes, CNVR boundaries and read coverages at CNVRs, serve as powerful tools to ascertain the veracity and potential phenotype-altering mechanisms of CNVRs, as well as the prevalence of individual CNV genotypes among breeds and in the populations studied.

Discussion

With the ever-reducing costs, WGS has become the method of choice for many applications involving CNV detection. Software to predict CNVs have also evolved and methods that rely on multi-sample read-depth analyses, like cn.MOPS, have become popular due to their superior ability to control for false discovery rate. cn.MOPS was used in the present study to analyse each of four WGS datasets which together represent over 500 bulls from 17 taurine cattle breeds. Besides CNV detection,

cn.MOPS provides integer copy number genotypes to indicate the level of deletion or amplification at the predicted CNVR. The detected CNVR genotypes allowed for clear separation of breeds by hierarchical clustering and were used to detect previously reported differences in the amplification levels at the *KIT* locus CNVR between Simmental and Hereford breeds. In future work, individual CNVR genotypes could be used in association analyses aimed at investigating the relationship between copy number and phenotype. In addition, we provide detailed annotation including sequencing read coverage for each CNVR in multiple samples representing the different genotypes identified. All results are presented in a unique, interactive visual database which allows the user to assess each CNVR based on sequence read alignments and to examine the boundaries of constituent CNVs in individual samples; such information is not available at a genome-wide scale in any of the previous published reports on CNVRs in any species. We believe that the way we present our results in the CNVR database better aligns with how this information will be used, that is, to investigate genomic regions or genes of interest for evidence of CNVs.

An important outcome from the present study was the necessity to address batch effects that could affect the reliability of CNVs predicted using algorithms that model read count variations across samples. The batch effects arise from genomic regions of imbalanced coverage across sequence datasets generated from different platforms and technologies. While the batch effects could potentially be controlled to an extent by including only those genomic regions that have adequate coverage across datasets, such an approach would have resulted in losing valuable information on CNVRs from individual datasets that had sufficient coverage at those regions. These observations guided our decision to analyse individual datasets separately. A direction for future research would be to develop methods to impute CNV genotypes at regions of low or no genomic coverage.

One limitation of the present study was that some of the breeds had low sample representation; the PIE, RDP, and BBL breeds had less than 10 samples each while the BAQ, DEV and SAL breeds had

only 1 sample each. Therefore, the breadth of breed-specific CNVRs reported are not as complete for those breeds as are those for the more popular breeds with greater sample representation in the present study. Nevertheless, CNVRs in some of those breeds with smaller representation (for example, DEV, SAL, BBL) have not been studied or reported earlier at a genome-wide scale, making this study amongst the first to do so in those breeds. Another limitation of the present study is that CNVRs shorter than 3000 bp are not reported, which was the limit we set for the dataset-wise analyses based on the sequencing coverage of samples in the dataset with the lowest mean coverage.

To conclude, the present study presents the most comprehensive CNVR collection in taurine cattle to date, which can serve as a reference on the locations of CNVRs and their genotype frequencies in a broad range of taurine cattle breeds. The visualizations and annotations included in the interactive databases greatly facilitate assessment of individual CNVRs and should aid the efforts to identify CNVRs that influence phenotype. We recommend that visualization of read coverage at predicted CNVRs be a standard protocol in studies reporting CNVRs on a genome-wide scale. Given the issue of false positive calls inherent to any prediction algorithm and the impracticality of experimental validation for CNVRs at a genome-wide scale, read coverage visualization at CNVRs offers a powerful way to not only overcome those issues but also to refine the CNVR boundaries, among other advantages. Further, we suggest integrating the NCBI Genome Data Viewer into analysis workflows, as we have done, as a way of assessing the NCBI and Ensembl gene models and their supporting evidence (RNA-Seq reads for example) when examining how CNVRs overlap with genome features.

Methods

Sequence data

The WGS datasets were generated in four different projects which together comprised 553 samples representing 1 taurine dairy cattle breed and 16 taurine beef cattle breeds (Table1 and Supplemental Table S1 online). The sequence data were generated following guidelines provided by the 1000 bull

genomes project (http://www.1000bullgenomes.com/) [50]. Details on animal selection, sequence generation and further analyses performed on datasets A and B have been published earlier [36,50]. Briefly, DNA samples were extracted from commercial artificial insemination bull semen straws and sequenced using either the 5500xl SOLiD™ system (85 animals) or the HiSeq™ 2000 system (298 animals). Reads that passed standard quality-based filtering criteria were aligned to the UMD3.1 bovine reference genome assembly [51] using *BWA-backtrack* algorithm of Burrows-Wheeler Aligner (BWA) [52] version 0.5.9. Local realignment of reads around indels was performed using *IndelRealigner* tool of the Genome Analysis Toolkit (GATK) [53] version 2.4, and duplicate reads marked using *MarkDuplicates* tool of the Picard toolkit version 1.54 (http://broadinstitute.github.io/picard/). Details on animal selection, sequence generation and further analyses performed on datasets C and D were similar to those for the previous datasets except for using more recent versions of the following software: BWA version 0.7.15 for dataset C and version 0.7.12 for dataset D, both using *BWA-MEM* algorithm, GATK version 3.5 and Picard toolkit version 2.0.1.

Identification of CNVs from sequence data

Detection of CNVs in the sequence data was performed using the Bioconductor [54] (version 3.6) package cn.MOPS [31] (version 1.24.0) of R (version 3.4.3) statistical programming language [55] running on a CentOS 7 Linux server with default cn.MOPS parameters except the following: *WL* 1000 bp and *rmdup* enabled to count only one read for each unique combination of position, strand and read width. CNVs were reported if 3 adjacent windows show significant read depth variations, thereby allowing detection of CNVs of length 3000 bp and higher in increments of 1000 bp.

Constructing CNVRs from CNVs

In cn.MOPS, CNVRs are constructed from CNVs by merging overlapping and adjacent CNVs using the *reduce* function from the Bioconductor package “GenomicRanges”. An initial test run on dataset A using that approach resulted in abnormally large CNVRs. Hence we followed a more conservative

approach to merge CNVs to CNVRs similar to what was used in some previous studies [26,29] in which CNVRs were constructed by merging only those CNVs across samples that satisfied a 50% pairwise reciprocal overlap criteria based on their genomic coordinates.

Assigning genotypes to CNVRs

By default, cn.MOPS assigns CNVR genotypes for each sample based on the genotypes of the CNVs comprising each CNVR. While the default approach worked well for the majority of cases, the selected genotype was not representative for 2.37 to 6.18% of the CNVRs across datasets where multiple discrete CNVs of differing genotypes occurred in certain individual samples. Such cases were observed more frequently for larger CNVRs. To assign CNVR genotypes, we used the genotype of the CNV type with the largest aggregate width amongst all CNV types comprising the CNVR; and in case of ties, we assigned the genotype that was closer to CN2. The corrected genotypes were used to perform genotype based hierarchical clustering of samples (using the *hclust* function in R with the *Spearman* correlation based distance measure and the *ward.D2* agglomeration method). Another issue with genotype assignment to CNVRs is associated with the 50% reciprocal overlap criterion that allows creation of overlapping CNVRs. In general, a CN2 genotype is assigned to samples where a CNV is not detected in a particular CNVRs; however, it is possible that the same sample may have a CNV of non-CN2 genotype detected on an overlapping CNVR. Therefore, we performed a CN2 correction as follows: for each test CNVR, the genotypes of samples for which cn.MOPS did not detect a CNV were changed from the default CN2 to CN_ in cases where a CNV was detected for that sample in another CNVR that overlapped with the test CNVR. The genotypes subsequently obtained were used for all summary calculations and plots created in the CNVR database.

Annotation of CNVRs

The CNVRs were annotated for genes based on information obtained from Ensembl [56,57] Release 88 (Bos_taurus.UMD3.1.88.gff3) and for cattle QTLs (99,652 QTLs) from Animal QTLdb [58] Release 33

(Aug 26, 2017) [https://www.animalgenome.org/cgi-bin/QTLdb/BT/index]. Information on segmental duplications in bovines was retrieved from sheet 1 of additional file 3 (Table S3.1-7) of a previous study [59] whereas assembly gaps and repeats were obtained for Bos_taurus_UMD_3.1/bosTau6 (Nov. 2009) assembly UCSC genome table browser (https://genome.ucsc.edu/cgi-bin/hgTables).

Hardy–Weinberg equilibrium (HWE) test on CNVR genotypes

We performed Pearson's chi-squared tests for goodness of fit of CNVR genotype proportions to HWE [60] at diallelic autosomal CNVRs with either a combination of CN0, CN1 and CN2 genotypes (considered as minor allele homozygous, heterozygous, and reference homozygous) or CN2, CN3 and CN4 genotypes (considered as reference homozygous, heterozygous, and minor allele homozygous), similar to a previous study [61]. The test was performed using the "HardyWeinberg" package [62] in R. Multi-allelic CNVR genotypes were not tested for HWE here because of the inability to determine what combination of alleles were responsible for a particular genotype. Furthermore, at all autosomal CNVRs, a parity test [63] was performed to test whether the number of individuals that have even CNVR genotypes (CN0, CN2, CN4 and CN8) exceed the number of individuals with odd CNVR genotypes (CN1, CN3, CN5 and CN7), an extension of the observation in SNP genotypes that, at HWE, the combined frequencies of the homozygote classes should exceed that of the heterozygote classes).

AVAILABILITY OF DATA AND MATERIALS

All data generated during this study are included in the article or as Supplemental files online. Raw sequence data for datasets A, B, C and D have been deposited to public databases (Sequence Read Archive (SRA) accessions SRP017441, SRP044884, SRP150844 and SRP153409 respectively). In addition, aligned sequence data for datasets A and B are available at GigaDB dataset ID 100157 (http://dx.doi.org/10.5524/100157). The CNVR databases per dataset are available via the GigaDB data repository (http://gigadb.org/).

ACKNOWLEDGMENTS

This research was supported by funding from Genome Canada, Genome Alberta, and Science Foundation Ireland (SFI) principal investigator award grant number 14/IA/2576. The analyses were performed, in part, using computing resources provided by WestGrid (http://www.westgrid.ca), Compute Canada (http://www.computecanada.ca) and Cybera (https://www.cybera.ca/).

AUTHOR CONTRIBUTIONS

PS and CFB designed the study. CFB, AB and DPB oversaw sample selection, acquisition and sequencing. AK, KK, AB and TRC performed sequence analysis and/or CNV detection. JRG developed the interactive CNV database. AK performed CNVR identification and downstream analyses steps and drafted the manuscript. All authors read, revised and approved the manuscript.

COMPETING INTERESTS

The authors declare that they have no competing interests in the manuscript.

REFERENCES

1. Feuk L, Carson AR, Scherer SW. Structural variation in the human genome. Nat Rev Genet. Nature Publishing Group; 2006;7:85–97.

2. Sudmant PH, Rausch T, Gardner EJ, Handsaker RE, Abyzov A, Huddleston J, et al. An integrated map of structural variation in 2,504 human genomes. Nature. Nature Publishing Group; 2015;526:75–81.

3. Keel BN, Lindholm-Perry AK, Snelling WM. Evolutionary and Functional Features of Copy Number Variation in the Cattle Genome. Front Genet. Frontiers Media SA; 2016;7:207.

4. Canales CP, Walz K. Copy number variation and susceptibility to complex traits. EMBO Mol Med.

Wiley-Blackwell; 2011;3:1–4.

5. Zarrei M, MacDonald JR, Merico D, Scherer SW. A copy number variation map of the human genome. Nat Rev Genet. Nature Publishing Group; 2015;16:172–83.

6. Prunier J, Caron | S Ebastien, Lamothe | Manuel, Blais | Sylvie, Bousquet J, Isabel N, et al. Gene copy number variations in adaptive evolution: The genomic distribution of gene copy number variations revealed by genetic mapping and their adaptive role in an undomesticated species, white spruce (Picea glauca). 2017;

7. Ricard G, Molina J, Chrast J, Gu W, Gheldof N, Pradervand S, et al. Phenotypic consequences of copy number variation: insights from Smith-Magenis and Potocki-Lupski syndrome mouse models. PLoS Biol. Public Library of Science; 2010;8:e1000543.

8. Fadista J, Nygaard M, Holm L-E, Thomsen B, Bendixen C. A Snapshot of CNVs in the Pig Genome. Kroymann J, editor. PLoS One. Public Library of Science; 2008;3:e3916.

9. Ramayo-Caldas Y, Castelló A, Pena RN, Alves E, Mercadé A, Souza CA, et al. Copy number variation in the porcine genome inferred from a 60 k SNP BeadChip. BMC Genomics. BioMed Central; 2010;11:593.

10. Paudel Y, Madsen O, Megens H-J, Frantz LA, Bosse M, Bastiaansen JW, et al. Evolutionary dynamics of copy number variation in pig genomes in the context of adaptation and domestication. BMC Genomics. BioMed Central; 2013;14:449.

11. Crooijmans RP, Fife MS, Fitzgerald TW, Strickland S, Cheng HH, Kaiser P, et al. Large scale variation in DNA copy number in chicken breeds. BMC Genomics. BioMed Central; 2013;14:398.

12. Yi G, Qu L, Liu J, Yan Y, Xu G, Yang N. Genome-wide patterns of copy number variation in the diversified chicken genomes using next-generation sequencing. BMC Genomics. BioMed Central; 2014;15:962.

13. Fontanesi L, Martelli P, Beretti F, Riggio V, Dall'Olio S, Colombo M, et al. An initial comparative map of copy number variations in the goat (Capra hircus) genome. BMC Genomics. BioMed Central; 2010;11:639.

14. Chen C, Qiao R, Wei R, Guo Y, Ai H, Ma J, et al. A comprehensive survey of copy number variation in 18 diverse pig populations and identification of candidate copy number variable genes associated with complex traits. BMC Genomics. BioMed Central; 2012;13:733.

15. Fadista J, Thomsen B, Holm L-E, Bendixen C. Copy number variation in the bovine genome. BMC Genomics. BioMed Central; 2010;11:284.

16. Liu GE, Hou Y, Zhu B, Cardone MF, Jiang L, Cellamare A, et al. Analysis of copy number variations among diverse cattle breeds. Genome Res. 2010;20:693–703.

17. Hou Y, Liu GE, Bickhart DM, Cardone MF, Wang K, Kim E, et al. Genomic characteristics of cattle copy number variations. BMC Genomics. BioMed Central; 2011;12:127.

18. Bae J, Cheong H, Kim L, NamGung S, Park T, Chun J-Y, et al. Identification of copy number variations and common deletion polymorphisms in cattle. BMC Genomics. BioMed Central; 2010;11:232.

19. Hou Y, Liu GE, Bickhart DM, Matukumalli LK, Li C, Song J, et al. Genomic regions showing copy number variations associate with resistance or susceptibility to gastrointestinal nematodes in Angus cattle. Funct Integr Genomics. 2012;12:81–92.

20. Jiang L, Jiang J, Wang J, Ding X, Liu J, Zhang Q. Genome-Wide Identification of Copy Number Variations in Chinese Holstein. Watson M, editor. PLoS One. Public Library of Science; 2012;7:e48732.

21. Hou Y, Bickhart DM, Hvinden ML, Li C, Song J, Boichard DA, et al. Fine mapping of copy number variations on two cattle genome assemblies using high density SNP array. BMC Genomics. BioMed Central; 2012;13:376.

22. Wu Y, Fan H, Jing S, Xia J, Chen Y, Zhang L, et al. A genome-wide scan for copy number variations using high-density single nucleotide polymorphism array in Simmental cattle. Anim Genet. Wiley/Blackwell (10.1111); 2015;46:289–98.

23. Bickhart DM, Hou Y, Schroeder SG, Alkan C, Cardone MF, Matukumalli LK, et al. Copy number variation of individual cattle genomes using next-generation sequencing. Genome Res. 2012;22:778–90.

24. Zhan B, Fadista J, Thomsen B, Hedegaard J, Panitz F, Bendixen C. Global assessment of genomic variation in cattle by genome resequencing and high-throughput genotyping. BMC Genomics. BioMed Central; 2011;12:557.

25. Stothard P, Choi J-W, Basu U, Sumner-Thomson JM, Meng Y, Liao X, et al. Whole genome resequencing of black Angus and Holstein cattle for SNP and CNV discovery. BMC Genomics. BioMed Central; 2011;12:559.

26. Keel BN, Keele JW, Snelling WM. Genome-wide copy number variation in the bovine genome detected using low coverage sequence of popular beef breeds. Anim Genet. 2017;48:141–50.

27. Chen L, Chamberlain AJ, Reich CM, Daetwyler HD, Hayes BJ. Detection and validation of structural variations in bovine whole-genome sequence data. Genet Sel Evol. BioMed Central; 2017;49:13.

28. Boussaha M, Esquerré D, Barbieri J, Djari A, Pinton A, Letaief R, et al. Genome-wide study of structural variants in bovine Holstein, Montbéliarde and Normande dairy breeds. PLoS One. 2015;10:1–21.

29. Letaief R, Rebours E, Grohs C, Meersseman C, Fritz S, Trouilh L, et al. Identification of copy number variation in French dairy and beef breeds using next-generation sequencing. Genet Sel Evol. BioMed Central; 2017;49:77.

30. Trost B, Walker S, Wang Z, Thiruvahindrapuram B, MacDonald JR, Sung WWL, et al. A Comprehensive Workflow for Read Depth-Based Identification of Copy-Number Variation from Whole-

Genome Sequence Data. Am J Hum Genet. Cell Press; 2018;102:142–55.

31. Klambauer G, Schwarzbauer K, Mayr A, Clevert DA, Mitterecker A, Bodenhofer U, et al. Cn.MOPS: Mixture of Poissons for discovering copy number variations in next-generation sequencing data with a low false discovery rate. Nucleic Acids Res. 2012;40:1–14.

32. Keel BN, Keele JW, Snelling WM. Genome-wide copy number variation in the bovine genome detected using low coverage sequence of popular beef breeds,. Anim Genet. 2017;48:141–50.

33. Olson TA. The genetic basis for piebald patterns in cattle. J Hered. Oxford University Press; 1981;72:113–6.

34. Fontanesi L, Tazzoli M, Russo V, Beever J. Genetic heterogeneity at the bovine *KIT* gene in cattle breeds carrying different putative alleles at the *spotting* locus. Anim Genet. Wiley/Blackwell (10.1111); 2010;41:295–303.

35. Whitacre L. Structural variation at the KIT locus is responsible for the piebald phenotype in Hereford and Simmental cattle. University of Missouri-Columbia; 2014.

36. Stothard P, Liao X, Arantes AS, De Pauw M, Coros C, Plastow GS, et al. A large and diverse collection of bovine genome sequences from the Canadian Cattle Genome Project. Gigascience. BioMed Central; 2015;4:49.

37. Agarwala R, Barrett T, Beck J, Benson DA, Bollin C, Bolton E, et al. Database resources of the National Center for Biotechnology Information. Nucleic Acids Res. Oxford University Press; 2018;46:D8–13.

38. Thorvaldsdottir H, Robinson JT, Mesirov JP. Integrative Genomics Viewer (IGV): high-performance genomics data visualization and exploration. Brief Bioinform. Oxford University Press; 2013;14:178–92.

39. Robinson JT, Thorvaldsdóttir H, Winckler W, Guttman M, Lander ES, Getz G, et al. Integrative genomics viewer. Nat Biotechnol. 2011;29:24–6.

40. Wright D, Boije H, Meadows JRS, Bed'hom B, Gourichon D, Vieaud A, et al. Copy Number Variation in Intron 1 of SOX5 Causes the Pea-comb Phenotype in Chickens. Stern DL, editor. PLoS Genet. Public Library of Science; 2009;5:e1000512.

41. Calvo JH, Iguácel LP, Kirinus JK, Serrano M, Ripoll G, Casasús I, et al. A new single nucleotide polymorphism in the calpastatin (CAST) gene associated with beef tenderness. Meat Sci. 2014;96:775–82.

42. Enriquez-Valencia CE, Pereira GL, Malheiros JM, de Vasconcelos Silva JAII, Albuquerque LG, de Oliveira HN, et al. Effect of the g.98535683A > G SNP in the CAST gene on meat traits of Nellore beef cattle (Bos indicus) and their crosses with Bos taurus. Meat Sci. 2017;123:64–6.

43. Tait RG, Shackelford SD, Wheeler TL, King DA, Casas E, Thallman RM, et al. µ-Calpain, calpastatin, and growth hormone receptor genetic effects on preweaning performance, carcass quality traits, and residual variance of tenderness in Angus cattle selected to increase minor haplotype and allele frequencies1,2,3. J Anim Sci. 2014;92:456–66.

44. Gill JL, Bishop SC, McCorquodale C, Williams JL, Wiener P. Association of selected SNP with carcass and taste panel assessed meat quality traits in a commercial population of Aberdeen Angus-sired beef cattle. Genet Sel Evol. 2009;41:36.

45. Casas E, White SN, Wheeler TL, Shackelford SD, Koohmaraie M, Riley DG, et al. Effects of calpastatin and micro-calpain markers in beef cattle on tenderness traits. J Anim Sci. 2006;84:520–5.

46. Tait RG, Shackelford SD, Wheeler TL, King DA, Keele JW, Casas E, et al. CAPN1, CAST, and DGAT1 genetic effects on preweaning performance, carcass quality traits, and residual variance of tenderness in a beef cattle population selected for haplotype and allele equalization1,2,3,4. J Anim Sci. Oxford University Press; 2014;92:5382–93.

47. Irwin DM. Evolution of the bovine lysozyme gene family: Changes in gene expression and reversion of function. J Mol Evol. Springer-Verlag; 1995;41:299–312.

48. Shin D-H, Lee H-J, Cho S, Kim H, Hwang J, Lee C-K, et al. Deleted copy number variation of Hanwoo and Holstein using next generation sequencing at the population level. BMC Genomics. BioMed Central; 2014;15:240.

49. Handsaker RE, Korn JM, Nemesh J, McCarroll SA. Discovery and genotyping of genome structural polymorphism by sequencing on a population scale. Nat Genet. Nature Publishing Group; 2011;43:269–76.

50. Daetwyler HD, Capitan A, Pausch H, Stothard P, van Binsbergen R, Brøndum RF, et al. Whole-genome sequencing of 234 bulls facilitates mapping of monogenic and complex traits in cattle. Nat Genet. 2014;46:858–65.

51. Zimin A V, Delcher AL, Florea L, Kelley DR, Schatz MC, Puiu D, et al. A whole-genome assembly of the domestic cow, Bos taurus. Genome Biol. BioMed Central; 2009;10:R42.

52. Li H, Durbin R. Fast and accurate short read alignment with Burrows-Wheeler transform. Bioinformatics. 2009;25:1754–60.

53. McKenna A, Hanna M, Banks E, Sivachenko A, Cibulskis K, Kernytsky A, et al. The Genome Analysis Toolkit: A MapReduce framework for analyzing next-generation DNA sequencing data. Genome Res. 2010;20:1297–303.

54. Gentleman RC, Carey VJ, Bates DM, Bolstad B, Dettling M, Dudoit S, et al. Bioconductor: open software development for computational biology and bioinformatics. Genome Biol. 2004;5:R80.

55. Ihaka R, Gentleman R. R: A Language for Data Analysis and Graphics. J Comput Graph Stat. 1996;5:299–314.

56. Aken BL, Ayling S, Barrell D, Clarke L, Curwen V, Fairley S, et al. The Ensembl gene annotation system. Database. Oxford University Press; 2016;2016:baw093.

57. Yates A, Akanni W, Amode MR, Barrell D, Billis K, Carvalho-Silva D, et al. Ensembl 2016. Nucleic

Acids Res. Oxford University Press; 2016;44:D710–6.

58. Hu Z-L, Park CA, Reecy JM. Developmental progress and current status of the Animal QTLdb. Nucleic Acids Res. Oxford University Press; 2016;44:D827–33.

59. Feng X, Jiang J, Padhi A, Ning C, Fu J, Wang A, et al. Characterization of genome-wide segmental duplications reveals a common genomic feature of association with immunity among domestic animals. BMC Genomics. BioMed Central; 2017;18:293.

60. Hardy GH. MENDELIAN PROPORTIONS IN A MIXED POPULATION. Science. American Association for the Advancement of Science; 1908;28:49–50.

61. Mei TS, Salim A, Calza S, Seng KC, Seng CK, Pawitan Y. Identification of recurrent regions of Copy-Number Variants across multiple individuals. BMC Bioinformatics. BioMed Central; 2010;11:147.

62. Graffelman J. Exploring Diallelic Genetic Markers: The **HardyWeinberg** Package. J Stat Softw. 2015;64:1–23.

63. Handsaker RE, Van Doren V, Berman JR, Genovese G, Kashin S, Boettger LM, et al. Large multiallelic copy number variations in humans. Nat Genet. NIH Public Access; 2015;47:296–303.

Figure legends

Figure 1. Batch effects among the 4 datasets contributing to inconsistent distribution of CNV genotypes in the analysis of the combined datasets

(a) PCA based on normalised read counts per segment showed separation by datasets and 4 outliers. (b) When datasets were combined and analysed together using cn.MOPS (*N*=549 after removing PCA outliers), the distribution of CNV genotypes revealed considerable differences among datasets (only autosomal CNVs are depicted here).

Figure 2. Distributions of CNV genotypes were more consistent across datasets that were analysed individually

When datasets were analysed individually (*N*=546 after removing PCA outliers and high coverage outlier samples in dataset A), the distribution of CNV genotypes were consistent among datasets (only autosomal CNVs are depicted here).

Figure 3. Proportions of overlapping CNVRs amongst datasets.

Pair-wise comparisons of the proportions of CNVRs in each dataset (rows; ordered by dataset size) that overlap by at least one base pair with CNVRs of other larger datasets (columns) are presented.

Figure 4. Prevalence and genotypes of the *KIT* locus CNV across breeds and datasets

The breed-wise prevalence and genotypes at CNVR Chr6:71747001-71752000, found approximately 45 Kb upstream of the *KIT* gene is depicted here. This CNVR has been reported to be associated with the piebald coat colour phenotype in HER and some SIM cattle, and occurs in high copy numbers in these breeds. The reason for detection of this CNVR in high copy number in 2 of the 22 CHA cattle in dataset B is attributed to potential issues with sourcing or handling of the respective samples.

Figure 5. Key features of the functionality of the CNVR database

The database has an index view and a detailed view with an option to enable/disable the help function on the top right of each page. The index page (a) has a panel (**Filters**) that allow users to apply filters to the CNVRs such as CNVR length or the number of samples that must contain the CNVR and the ability to exclude/include specific samples based on regular expression matches. Another panel (**Statistics**) provides summary information on the CNVRs before and after applying the filters. The remaining panels on the index page allow users to search and sort on **CNVRs**, overlapping **genes** and **QTLs** and/or **samples** to quickly find CNVRs associated with a particular gene/QTL. All or selected data can be exported as CSV files. CNVRs of interest can be noted as favorites; and comments can be

added for individual CNVRs. All comments, filters and/or favorites can be saved as a text file that can be reloaded later using the **Settings** button options on the top right of the page. Clicking on a CNVR provides a detailed view (b) with panels displaying basic statistics on the CNVR (**Summary**), a bar plot of the number of samples per CNV genotype (**Genotype distribution**) and another bar plot of the number of non-CN2 variants per breed (**Breed distribution**), graphical representation of the CNVR in genomic context (**Overlapping genes, QTLs and CNVs**), sequence read coverage at the CNVR for up to 3 samples per genotype (**IGV images**), a table of all the samples indicating the CNV genotype (**CNVR-specific sample list**) and finally a **sample view** that provides for the selected sample, a graphical representation of the CNVR and CNV in genomic context with overlapping genes and QTLs.

Additional file legends

Figure S1: Sample-wise sequencing coverages per dataset.

Figure S2-S5: Proportions of the different CNV genotypes identified per sample (a), distributions of CNV genotype counts (b), proportions of DELs among CNVs (c) and total CNVs discovered (d) per dataset.

Figure S6-S9: Hierarchical clustering of samples based on the CNVR genotypes per dataset.

Figure S10: Chromosome-wise counts of total CNVRs and CNVRs per category (DEL, AMP, MIX) for datasets A (a), B (b), C (c) and D (d).

Figure S11: Phenograms representing the chromosomal locations of CNVRs belonging to the different categories for datasets A (a), B (b), C (c) and D (d).

Figure S12-S16: Specific examples to depict exploration of the CNVR databases for variants of interest.

Table S1: Detailed information on samples and breed code translations.

Table S2: List of CNVRs discovered in each dataset with the respective CNVR category assignments.

Table S3: Breed-wise summaries of CNVRs identified per dataset.

Table S4: Breed-specific CNVRs found in datasets A, B and C.

Figure 1

Click here to download Figure Figure1.pdf

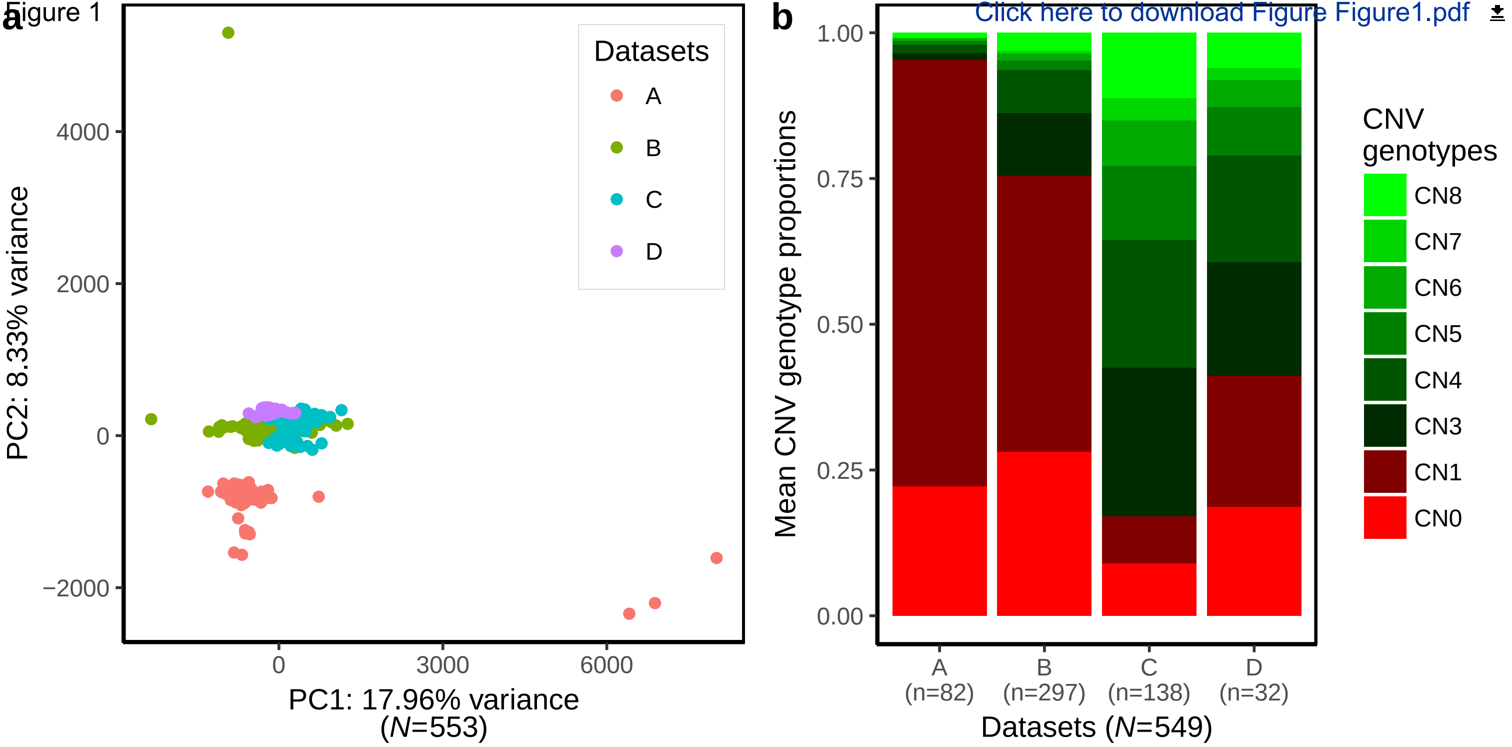

Figure 2

Click here to download Figure Figure2.pdf

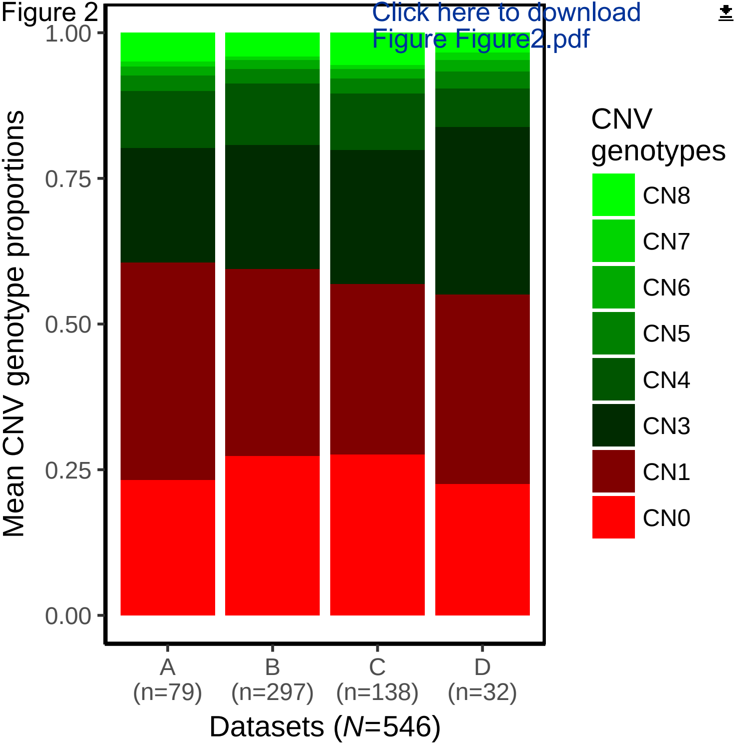

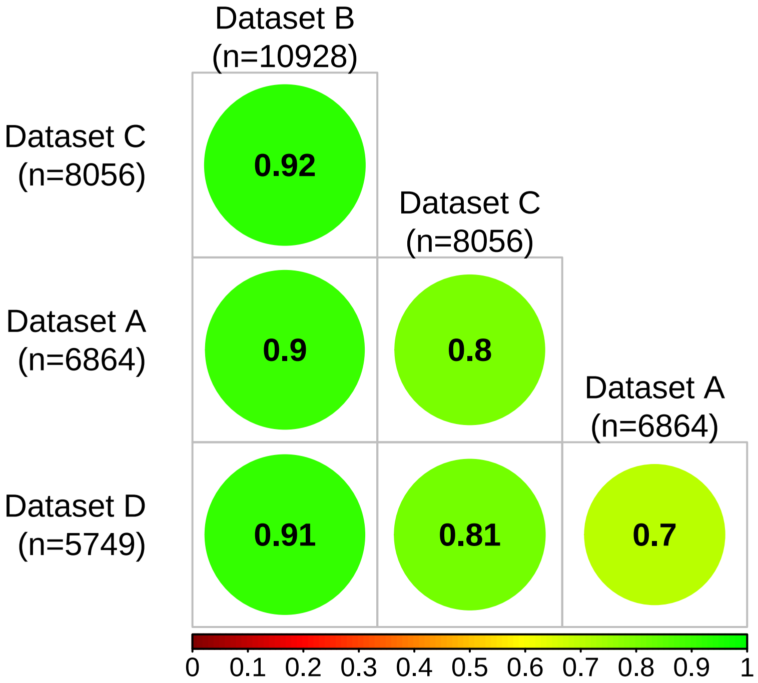
Dataset B
(n=10928)
Dataset C
(n=8056)
Dataset A
(n=6864)
Dataset C
(n=8056)
Dataset A
(n=6864)
Dataset D
(n=5749)
0.92
0.9
0.8
0.91
0.81
0.7
0
0.1
0.2
0.3
0.4
0.5
0.6
0.7
0.8
0.9
1

Figure 4

Click here to download Figure Figure4.pdf

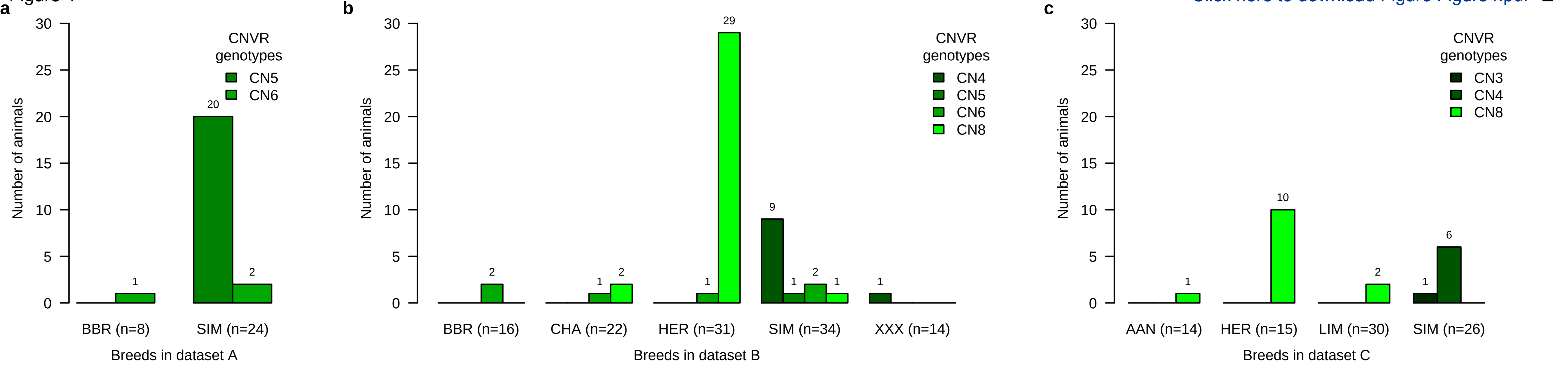

Figure 5 Click here to download Figure Figure5.png

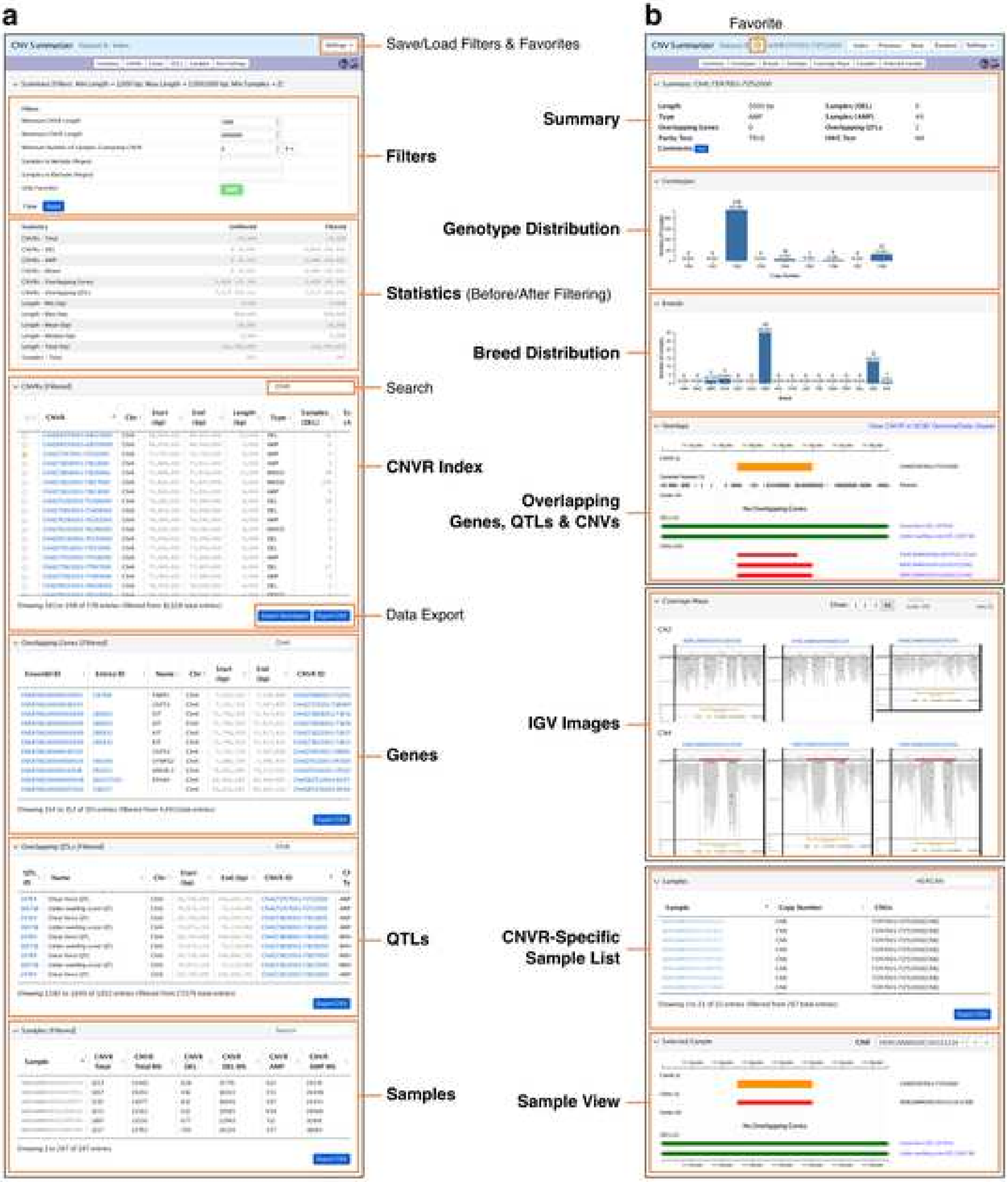

Figure S1

Click here to access/download
Supplementary Material
Supplemental_Figure_S1.pdf

Figure S2

Click here to access/download
Supplementary Material
Supplemental_Figure_S2.pdf

Figure S3

Click here to access/download
Supplementary Material
Supplemental_Figure_S3.pdf

Figure S4

Click here to access/download
Supplementary Material
Supplemental_Figure_S4.pdf

Figure S5

Click here to access/download

Supplementary Material

Supplemental_Figure_S5.pdf

Figure S6

Click here to access/download
Supplementary Material
Supplemental_Figure_S6.pdf

Figure S7

Click here to access/download

Supplementary Material

Supplemental_Figure_S7.pdf

Figure S8

Click here to access/download
Supplementary Material
Supplemental_Figure_S8.pdf

Figure S9

Click here to access/download
Supplementary Material
Supplemental_Figure_S9.pdf

Figure S10

Click here to access/download
Supplementary Material
Supplemental_Figure_S10.pdf

Figure S11

Click here to access/download
Supplementary Material
Supplemental_Figure_S11.pdf

Figure S12

Click here to access/download
Supplementary Material
Supplemental_Figure_S12.pdf

Figure S13

Click here to access/download
Supplementary Material
Supplemental_Figure_S13.pdf

Figure S14

Click here to access/download
Supplementary Material
Supplemental_Figure_S14.pdf

Figure S15

Click here to access/download

Supplementary Material

Supplemental_Figure_S15.pdf

Figure S16

Click here to access/download
Supplementary Material
Supplemental_Figure_S16.pdf

Table S1

Click here to access/download
Supplementary Material
Supplemental_Table_S1.xlsx

Table S2

Click here to access/download
Supplementary Material
Supplemental_Table_S2.xlsx

Table S3

Click here to access/download
Supplementary Material
Supplemental_Table_S3.xlsx

Table S4

Click here to access/download
Supplementary Material
Supplemental_Table_S4.xlsx
