# Supplementary material for: A large interactive visual database of copy number variants discovered in taurine cattle
Source: Gigascience. 2019 Jun 26;8(6):giz073. doi: 10.1093/gigascience/giz073 (PMC6593363; doi:10.1093/gigascience/giz073)
Supplement: giz073_GIGA-D-18-00350_Revision_1 [file giz073_giga-d-18-00350_revision_1.pdf]

GigaScience

A large interactive visual database of copy number variants discovered in taurine cattle

--Manuscript Draft--

**Manuscript Number:**	GIGA-D-18-00350R1
**Full Title:**	A large interactive visual database of copy number variants discovered in taurine cattle
**Article Type:**	Research
**Funding Information:**	Science Foundation Ireland (14/IA/2576) — Dr Donagh P. Berry<br>Genome Alberta — Dr Paul Stothard<br>Genome Canada — Dr Christine F. Baes, Dr Paul Stothard<br>Science Foundation Ireland and the Department of Agriculture, Food and Marine on behalf of the Government of Ireland (16/RC/3835 (VistaMilk)) — Dr Donagh P. Berry

Abstract:

Background
Copy number variants (CNVs) contribute to genetic diversity and phenotypic variation. We aimed to discover CNVs in taurine cattle using a large collection of whole-genome sequences and to provide an interactive database of the identified CNV regions (CNVRs) that includes visualisations of sequence read alignments, CNV boundaries and genome annotations.

Results
CNVs were identified in each of four whole genome sequencing datasets, which together represent over 500 bulls from 17 breeds, using a popular multi-sample read-depth based algorithm, cn.MOPS. Quality control and CNVR construction, performed dataset-wise to avoid batch effects, resulted in a total of 26,223 CNVRs covering 107.75 unique megabases (4.05%) of the bovine genome. Hierarchical clustering of samples by CNVR genotypes indicated clear separation by breeds. An interactive HTML database was created that allows data filtering options, provides graphical and tabular data summaries including Hardy-Weinberg equilibrium tests on genotype proportions, and displays genes and quantitative trait loci at each CNVR. Notably, the database provides sequence read alignments at each CNVR genotype and the boundaries of constituent CNVs in individual samples. Besides numerous novel discoveries, we corroborated the genotypes reported for a CNVR at the KIT locus known to be associated with the piebald coat colour phenotype in Hereford and some Simmental cattle.

Conclusions
We present a large comprehensive collection of taurine cattle CNVs in a novel interactive visual database that displays CNV boundaries, read depths and genome features for individual CNVRs, thus providing users with a powerful means to explore and scrutinise CNVRs of interest more thoroughly.

**Corresponding Author:**	Paul Stothard<br><br>CANADA
**Corresponding Author Secondary Information:**	
**Corresponding Author's Institution:**	
**Corresponding Author's Secondary Institution:**	
**First Author:**	Arun Kommadath
**First Author Secondary Information:**	

Powered by Editorial Manager® and ProduXion Manager® from Aries Systems Corporation

Order of Authors:	Arun Kommadath
	Jason R. Grant
	Kirill Krivushin
	Adrien M. Butty
	Christine F. Baes
	Tara R. Carthy
	Donagh P. Berry
	Paul Stothard
Order of Authors Secondary Information:	
Response to Reviewers:	February 27, 2019<br><br>Dear Editorial Board of GigaScience,<br><br>We are pleased to submit a revised version of our manuscript where we addressed all comments from the reviewers. We found the reviews very useful and thank the reviewers for their time and effort.<br><br>Please note that the order of authors generated earlier through Gigascience's submission portal was different from how it is in the manuscript. Also, one of our co-authors has requested to add an initial for his middle name. We were able to make these changes ourselves through Gigascience's submission portal.<br><br>We thank you for receiving our revised manuscript and look forward to a positive review.<br><br>Sincerely,<br><br>Paul Stothard<br>(Corresponding author on behalf of all authors)<br><br>==<br>Response to reviewers' comments:<br>==<br><br>==<br>Reviewer #1:<br>==<br><br>This manuscript describes the discovery and genotyping of copy number variations (CNVs) in multiple datasets using a single CNV algorithm together with the development of a platform that integrates multiple tools to allow identified CNVs to be easily assessed visually in multiple ways. The development of the platform will be of great use to the field of CNVs as streamlines the otherwise laborious task of characterizing individual CNVs. The manuscript describes selected CNVs as examples of this platform. The manuscript is well written and represents a significant advance to the field. There are some points in the manuscript I feel could be improved upon:<br><br>We thank you for your thorough review and valuable comments that helped us improve our manuscript. We have addressed your comments as listed below and highlighted in yellow the corresponding changes in the revised manuscript. Some other changes that we made, mostly grammatical, are highlighted green.<br><br>==<br><br>Lines 45-48 The authors say they are presenting the "the largest CNV set". However, no genome wide validation of the CNVs appears to have been undertaken (although

the database described is set up in a manner to allow researchers to validate). Given that CNV discovery is plagued by false positives having the "largest set of CNVs " does not necessarily mean the best set - producing the largest set of putative CNVs is easy, but determining which are real, is hard. These sentences should be rewritten, particularly given that later in the manuscript the authors do acknowledge gaps affect the detection of CNVs.

Thanks for pointing this out. We do understand that the size of the predicted CNVs does not mean much unless validated. What we wanted to emphasize was the breath of this set of CNVs, which was generated using over 500 bulls from 17 different breeds. We have rephrased the sentence as follows (Page 2; Line 45):
"We present a large comprehensive collection of taurine cattle CNVs in a novel interactive visual database that displays CNV boundaries, read depths and genome features for individual CNVRs, thus providing users with a powerful means to explore and scrutinize CNVRs of interest more thoroughly."

==

Line 75-81 The authors indicate that there has been no genome wide validation of CNVs called by various CNV algorithms, however, Couldrey et al 2017 - this paper did successfully undertake genome wide assessment (Detection and assessment of copy number variation using PacBio long-read and Illumina sequencing in New Zealand dairy cattle). The introduction should be amended to include this information, the manner in which the authors wrote this sentence implies that they have undertaken genome wide validation which they have not done.

Thanks for bringing this omission to our notice. We have referenced that study and added the following to the introduction (Page 4; Lines 79-91):
"Couldrey et al. [31] illustrated the use of long-read sequence information combined with a CNV transmission-based approach to confirm a subset of CNVs that segregate in the New Zealand dairy cattle population. Briefly, the putative CNVs discovered from long-read sequence information in a prominent Holstein-Friesian bull used in New Zealand were first compared with those discovered from short-read sequences in the same bull. Next, a population of 556 cattle representing the wider New Zealand dairy cattle population were short-read sequenced and genotyped at those putative CNV regions, followed by a genome-wide assessment of transmission level of copy number based on pedigree. Visual assessment of highly transmissible CNV regions provided additional evidence to support the presence of CNV across the sequenced animals. Currently, the high cost of long-read sequencing limits adoption of this approach to large numbers of animals representing different breeds, and other studies that provide supportive evidence on a genome-wide scale to help assess the quality of CNVs predicted from short-read sequencing or SNP array data are extremely limited."

==

Please include further discussion about the accuracy of breakpoints for CNVs and CNVRs, are these consistent between samples, if not, how does this affect analysis between samples and accuracy of assigned copy number genotype? Please also comment further on how the same genomic location was defined, if a CNVR in one animal was a subset of a larger one in another (or there was an overlap between animals) were these counted as unique or separate CNVRs

The breakpoints for CNVs at a CNVR are not always consistent across all samples in which they are identified and this does not mean the breakpoints identified are inaccurate as can be verified from the visualisation provided for read alignments at the CNVR. A consequence of having multiple adjacent CNVs at a CNVR that vary in their breakpoints by one or more windows of 1 Kb (the window size parameter we chose for cn.MOPS algorithm) is that a simple merging of those CNVs using the default CNVR construction method in cn.MOPS would result in some CNVRs that are abnormally large. Also, we would then lose information on the CNV diversity across samples at that CNVR and the genotype assigned to that CNVR may not be representative of the underlying CNVs. Our solution to avoid those issues was to use a 50% pairwise

	reciprocal overlap criterion to construct CNVRs following which genotypes were assigned based on a set of rules as described in the Methods section. Therefore, at the same genomic location, if a CNV in one animal was a subset of a larger one in another, the decision to merge them into a single CNVR or to keep them as separate CNVRs was dependent on the mutual overlap between those CNVs. More details on these points can be found in sections "Constructing CNVRs from CNVs" and "Assigning genotypes to CNVRs" of the Methods section. Also, to emphasize the above points, we have added the following to the Discussion section:<br>Page 19; Lines 421-427:<br>"We did not use the built-in function within cn.MOPS to construct CNVRs and assign CNVR genotypes as we found that this approach can produce very large CNVRs which obscure the underlying breakpoint diversity across samples and that have genotype assignments that are not always consistent with the majority genotype observed among the constituent CNVs. We therefore employed a 50% pairwise reciprocal overlap criterion to construct CNVRs, as has been used in other studies [26,29] and then assigned genotypes based on a set of rules as described in the Methods section."<br>Page 19; Lines 435-440<br>"Read coverage and alignments within and adjacent to a CNVR can aid in the determination of the breakpoints of constituent CNVs in individual samples, as the resolution of the breakpoints reported by the cn.MOPS algorithm is limited to the choice of window size used for CNV detection. The visualisation of genome features like assembly gaps and repeats can highlight potential non-CNV related coverage and alignment anomalies, and thus can further be used in the assessment of predicted CNVs and their breakpoints."<br><br>===<br><br>Line 214 please indicate if the CNVR upstream of KIT is the causative as determined by J. Taylors lab or another CNVR in the same area (also reported by the Taylor lab).<br><br>We had looked into that CNVR too and we have added the following lines to the Results section “Identification and genotyping of the well-characterised KIT locus CNV in our datasets” to address this (Page 13: Lines 257-263):<br>"Another CVNR, approximately 15 Kb in size (Chr6:71810000-71825000) and located within intron 1 of the KIT gene, has been reported to be associated with the piebald coat color [36] In our analysis, the only CNVR that overlaps with this region and that shows amplification in the majority of HER and some SIM animals is an 11 Kb CNVR at Chr6:71808000-71819000, identified only in dataset B. This CNVR was detected in 25 of the 31 HER (24 as CN3 and 1 as CN8) and 7 of the 34 SIM (all as CN3) individuals in dataset B. Thus based on our results, the CNVR at Chr6:71747001-71752000 (upstream of the KIT gene) is more clearly associated with the piebald coat color."<br><br>===<br><br>Given that historically deletions have been significantly easier to detect from short read sequence using read depth please add some discussion on the low frequency of deletion detection relative to duplication<br><br>Following quality control (QC), the frequency of DEL type CNVs was still higher than AMP type in all our datasets with means of the proportions of DELs ranging between 0.57 (SD 0.06) for dataset C and 0.60 (SD 0.07) for dataset B. Samples with outlier proportions of DEL type CNVs, generally seen in low coverage samples (see Figures S2-S5), were removed during QC as indicated in the Results section, "Distributions of CNV genotypes were more consistent across datasets that were analysed individually":<br>"Additional quality control (QC) steps were applied to identify problematic samples, defined as those that showed marked deviations (i.e., 1.5 times the interquartile range away from the first and third quartiles) in the proportion of DELs or total CNVs discovered within each dataset."<br><br>===<br><br>===

Powered by Editorial Manager® and ProduXion Manager® from Aries Systems Corporation

Reviewer #2
==

This paper describes CNV(R) detection in sequenced cattle of four different datasets. It highlights some important issues that may arise during CNV detection in data from different sequencing platforms, but also in CNV detection in general. The authors have provided a valuable database for future research that can serve as a resource to confirm CNVs and visualize them.

We thank you for your thorough review and valuable comments that helped us improve our manuscript. We have addressed all your comments as listed below and highlighted in yellow the corresponding changes in the revised manuscript. Some other changes mostly grammatical that we made are highlighted green.

==

I have not been able to find the actual database on GigaDB. This makes it hard to value the usefulness.

The database was uploaded on GigaDB but the editor had informed us about the delay in making it available to reviewers. It should be accessible now.

==

My main remark is that there are a number of recommendations done, for which the reason and tools to do so are lacking (e.g., HWE) or unrealistic (e.g., confirm genome wide CNV visually), see the line by line comments. There are many methods to predict CNV, the authors seem very confident about their approach, but other than the platform effect, there are limited results shown to back that. Visualizing seems to be the key. However, this is very unpractical when screening the whole genome of hundreds of individuals. On the other hand, when interested in a specific CNV visualisation to confirm the CNV is needed, and then this database would be useful for confirmation.

HWE is provided as an additional annotation, however, we do not recommend its use as a criterion to filter CNVRs. We edited Lines 296-299 (Page 14) to clarify this: "HWE results are provided as an additional characteristic / annotation of CNVRs but we caution against filtering CNVRs based on HWE as the test is limited to diallelic autosomal CNVRs and deviations from HWE could reflect inaccurate genotypes for an otherwise true CNVR of interest."

The strength of our database, as you indicate and we emphasize, is to provide the user the ability to assess individual CNVRs of interest (for example, CNVRs in the vicinity of a candidate gene or GWAS peak) among CNVRs identified on a genome wide scale. We do not claim to provide genome wide validation of CNVRs.

Our rationale for the choice of the CNV prediction software used in our research is discussed in our response to one of your specific comments on this that follow.

==

L122 You refer to 'batch effect' here however to me it seems a sequencing platform effect. Can different batches sequenced on the same platform with similar coverage be combined? Was that the case here? With different batches I mean a group of animals sequenced in January and another group in June for instance (same platform, same intended coverage).

We call this a “batch effect” and not a “sequencing platform effect” as we found that samples sequenced using the same platform (Illumina) but differing in read sequence length despite similar coverage also clustered separately based on CNV genotypes. The time of sequencing may have had an effect too through differences in sequencing chemistries within the same sequencing platform technology.

==

Powered by Editorial Manager® and ProduXion Manager® from Aries Systems Corporation

L122 Please indicate that the CNV discovery referred to here was done in the complete dataset

We have rephrased the sentence to indicate this (Page 6; Line 135):
"Proceeding with CNV discovery and genotype characterization using those read counts from all datasets together (after excluding the four PCA outliers) revealed considerable differences in the distribution of CNV genotypes per dataset (Figure 1b)."

==

L129 Change figure 1b into figure 1a

There is no reference to figures at this line. The references to Figure 1 in the previous lines have been verified to be correct.

==

L152-153 When you remove samples should you not rerun CNV detection?

Reruns of the CNV detection algorithm after removing samples based on outlier status might still detect new samples as outliers. So we perform the sample filtering only once to capture the most significant outliers and remove them. Further, of the CNVRs generated from the filtered set of CNVs, only those that were identified in at least 2 samples were kept. The quality of the resulting CNVRs is evident based on the clear clustering of samples by breed (Figures S6-S9) based on CNVR genotypes.

==

L243-244 Which version number of Chrome/Firefox is that? Or will this be true in the future as well?

Either browser updated to a recent version, not necessarily “latest” should work fine. Future versions of those browsers should be ok too as they would be built to be backward compatible and we have used standard features of html and JavaScript to provide functionality. In L270 (Page 13), the word "latest" has been replaced with "recent":
"(for best results, use a recent version of Google Chrome or Mozilla Firefox)."

==

L254 Does this mean that the CNVs detected with UMD3.1 can now be plotted in the new genome build ARS-UCD 1.2?

It is possible to map the CNVRs we identified on UMD3.1 onto ARS-UCD1.2 using liftOver coordinates now available for this assembly. However, we have not currently implemented that feature in our database. We have edited L281 (Page 13) to clarify that the links we generated to view CNVRs on the NCBI genome data viewer are also based on UMD3.1.
“Additionally, a link to the NCBI Genome Data Viewer (www.ncbi.nlm.nih.gov/genome/gdv/) [38] plots the CNVR region in the context of the latest annotations and genomics data available in NCBI for the UMD3.1.1 bovine reference genome assembly.”

==

L261 Were HWE and parity test part of the QC mentioned in table 2?

No, they were not used in the QC but are provided as an additional resource to users.

==

L264-266 This sentence confused me. If 63-88% of the CNVR that passed HWE had a

	p-value >=10exp-5 what was then your threshold to qualify HWE? It is also not given in M&M.<br><br>As mentioned in section "Hardy–Weinberg equilibrium (HWE) test on CNVR genotypes" of the Methods section, the CNVRs that qualified for HWE testing were diallelic autosomal CNVRs with either a combination of CN0, CN1 and CN2 genotypes (considered as minor allele homozygous, heterozygous, and reference homozygous) or CN2, CN3 and CN4 genotypes (considered as reference homozygous, heterozygous, and minor allele homozygous). Multi-allelic CNVR genotypes were not tested for HWE here because of the inability to determine what combination of alleles were responsible for a particular genotype. We edited Line 291 (Page 14) to include "diallelic autosomal" CNVRs:<br>"Of the diallelic autosomal CNVRs that qualified for the HWE test per dataset (53-57% of the total for the 4 datasets; see Methods), the majority (63-88%) had genotype proportions that were in HWE (Chi-squared test p-value ≥ 10-5)."<br><br>==<br><br>L268-270 Why do you recommend this and how should one verify this? It seems that you know something you forgot to inform us about. Why not simply discard them, as we do with SNPs?<br><br>We have replaced that line with the following clarification (Lines 296-299; Page 14):<br>"HWE results are provided as an additional characteristic / annotation of CNVRs but we caution against filtering CNVRs based on HWE as the test is limited to diallelic autosomal CNVRs and deviations from HWE could reflect inaccurate genotypes for an otherwise true CNVR of interest."<br><br>==<br><br>L287-288 I didn't see this discussion in previous section. Yes HWE was mentioned but there is no proper discussion on the reasons of deviation from HWE and how your database can verify them. So please add. See also previous comment.<br>We have edited Lines 316-319 (Page 15) to address this:<br>"As discussed in the previous section, deviations from HWE should not be used as a criterion to filter CNVRs; instead visualisation of the read coverage and other supporting information at the CNVR available through the CNVR database will help validate the predicted CNVs."<br><br>==<br><br>L313 Remove additional '.' at end of sentence<br><br>Thanks, this is now corrected.<br><br>==<br><br>L386-387 Can you back this superiority with a reference? Are methods that combine for instance read depth and paired end reads not better?<br><br>There is no consensus on what method is best as different methods have different strengths and weaknesses. Methods combining read depth and paired-end information will have some strengths that cn.MOPS does not and vice-versa. The one obvious strength in a multi-sample test like cn.MOPS is their superior ability to control for false discovery rate. While the issue of FDR can be moderated in approaches that use paired-end read information, this limits their discovery power, especially in regions of low coverage. These points are all discussed in the cn.MOPS paper along with benchmarks on the performance of cn.MOPS against 5 other methods that showed cn.MOPS performed significantly better than those methods based on precision-recall curves; we therefore reference the cn.MOPS paper here in Lines 417-421 (Page 18) along with another paper by Keel et al. (2017) where it was shown through simulation studies that read-depth based methods (which included cn.MOPS) performed better than paired end and split read based methods in the majority of datasets composed of samples at carrying levels of sequencing coverage:

"Software to predict CNVs has also evolved and methods that rely on multi-sample read-depth analyses, like cn.MOPS, have become popular due to their superior ability to control for false discovery rate [32]. Furthermore, a recent study on simulated data has reported read depth based approaches to perform relatively better than those based on paired end and split read analyses when analyzing datasets comprised of samples sequenced at varying levels of coverage [26]."

==

L410-411 Interesting comment about imputation. Like to get your thoughts on this subject. Based on what can you perform imputation for CNV? Pedigree? SNPs? The SNP-CNV LD seems very limited for imputation, but perhaps there is more information in sequence data.

We have looked into this further and choose to remove this statement in light of additional reading which revealed the challenges involved in imputation of CNVs from SNP data and the non-availability of pedigree information for our datasets.

==

L427-429 Do you mean visualizing each CNVR one by one? Noble recommendation, but that is a pain in the ass job, for instance for 26000 CNVR detected in your study. Have you visualized them all? I assume only the ones you report in detail about. Isn't that the reason why we apply methods like cn.MOPS, to automate the job. I know that CNV calling methods have their issues, but on a genome wide scale checking each detected CNV manually seems a bit too much to do, especially as sample sizes are getting bigger and bigger. I agree that CNVR of interest that will be explored further because of association or something, they should be manually checked, but genome wide is a bit much I would say, unless you can automate it.

The strength of our database, as you indicate and we emphasize, is to provide the user the ability to verify individual CNVRs of interest (for example near to a gene or genome region highlighted through some other research activities) among CNVRs identified on a genome wide scale. We do not claim to provide genome wide validation of CNVRs and we have added/edited the sentence (Page 21; Lines 468-470) to avoid implying that:
"We recommend that visualisation of read coverage at predicted CNVRs be a standard protocol in studies reporting specific CNVRs of interest (for example near to a gene or genome region highlighted through some other research activities) among CNVRs identified on a genome-wide scale."

==

L457 Can you add why you chose cn.MOPS? There are so many methods, and as you indicated the method is relevant.

Please refer to our earlier response to your comment regarding, “Can you back this superiority with a reference”. Our datasets consisted of large number of samples sequenced at moderate coverage, we could leverage the strength of cn.MOPS algorithm to control FDR through its multi-sample approach.

==

Figure 4 more distinctive colours for the bars would be appreciated

The colours used here are kept consistent with the colours we used in several other plots to represent genotypes: gradients of red for DELs and green for AMPs. Hence we keep to the same colour scheme here. However, to assist with interpretation, we have added a genotype label at the base of each bar in the revised figure 4.

==

Powered by Editorial Manager® and ProduXion Manager® from Aries Systems Corporation

	Figure 5 Hard to see what is in the panels, and the database itself I could not find on GigaDB. Also in Supplementary material figure S12-16.<br><br>The editor had informed us of the delay in making the database available for the first review but that it is now available to reviewers.<br>Figure 5 is meant to be an overall illustration of the key features of the functionality of the CNVR database, and therefore we prefer to keep those panels in one figure. We understand the panels would not be readable in its original size when printed on paper, and hence we had annotated them with labels and described each panel in the accompanying legend. However we did note resolution issues with some figures and have recreated several at higher resolution including figures S12-16, so that users can view them at a larger size / zoom in to see much more detail.<br><br>==
**Additional Information:**	
**Question**	**Response**
Are you submitting this manuscript to a special series or article collection?	No
**Experimental design and statistics**<br><br>Full details of the experimental design and statistical methods used should be given in the Methods section, as detailed in our Minimum Standards Reporting Checklist. Information essential to interpreting the data presented should be made available in the figure legends.<br><br>Have you included all the information requested in your manuscript?	Yes
**Resources**<br><br>A description of all resources used, including antibodies, cell lines, animals and software tools, with enough information to allow them to be uniquely identified, should be included in the Methods section. Authors are strongly encouraged to cite Research Resource Identifiers (RRIDs) for antibodies, model organisms and tools, where possible.<br><br>Have you included the information requested as detailed in our Minimum Standards Reporting Checklist?	Yes
**Availability of data and materials**	No

All datasets and code on which the conclusions of the paper rely must be either included in your submission or deposited in publicly available repositories (where available and ethically appropriate), referencing such data using a unique identifier in the references and in the "Availability of Data and Materials" section of your manuscript.<br><br>Have you have met the above requirement as detailed in our Minimum Standards Reporting Checklist?	
If not, please give reasons for any omissions below.<br><br>as follow-up to "**Availability of data and materials**<br><br>All datasets and code on which the conclusions of the paper rely must be either included in your submission or deposited in publicly available repositories (where available and ethically appropriate), referencing such data using a unique identifier in the references and in the "Availability of Data and Materials" section of your manuscript.<br><br>Have you have met the above requirement as detailed in our Minimum Standards Reporting Checklist?<br><br>"	Data to be made available through GigaDB require a manuscript ID first, so we will submit them soon after we receive the ID.

Revised manuscript
Click here to access/download;Manuscript;revised_manuscript.docx
Click here to view linked References

A large interactive visual database of copy number variants discovered in taurine cattle

Arun Kommadath[1], Jason R. Grant[1], Kirill Krivushin[1], Adrien M. Butty[2], Christine F. Baes[2,3], Tara R. Carthy[4], Donagh P. Berry[4] and Paul Stothard[1*]

[1] Department of Agricultural, Food and Nutritional Science (AFNS), University of Alberta, Edmonton, Alberta, Canada

[2] Centre for Genetic Improvement of Livestock, Department of Animal Biosciences, University of Guelph, Guelph, Canada

[3] Institute of Genetics, Vetsuisse Faculty, University of Bern, Bern, Switzerland

[4] Teagasc, Animal & Grassland Research and Innovation Centre, Moorepark, Fermoy, Ireland

[*] Corresponding author

Email addresses:

Arun Kommadath: kommadat@ualberta.ca

Jason R. Grant: jason.grant@ualberta.ca

Kirill Krivushin: krivushi@ualberta.ca

Adrien M. Butty: buttya@uoguelph.ca

Christine F. Baes: cbaes@uoguelph.ca

Tara R. Carthy: tara.carthy@teagasc.ie

Donagh P. Berry: donagh.berry@teagasc.ie

Paul Stothard: stothard@ualberta.ca

Keywords: CNV, structural variants, cattle, dairy, beef, whole-genome sequencing, database, sequence visualisation

ABSTRACT

Background

Copy number variants (CNVs) contribute to genetic diversity and phenotypic variation. We aimed to discover CNVs in taurine cattle using a large collection of whole-genome sequences and to provide an interactive database of the identified CNV regions (CNVRs) that includes visualisations of sequence read alignments, CNV boundaries and genome annotations.

Results

CNVs were identified in each of four whole genome sequencing datasets, which together represent over 500 bulls from 17 breeds, using a popular multi-sample read-depth based algorithm, cn.MOPS. Quality control and CNVR construction, performed dataset-wise to avoid batch effects, resulted in a total of 26,223 CNVRs covering 107.75 unique megabases (4.05%) of the bovine genome. Hierarchical clustering of samples by CNVR genotypes indicated clear separation by breeds. An interactive HTML database was created that allows data filtering options, provides graphical and tabular data summaries including Hardy-Weinberg equilibrium tests on genotype proportions, and displays genes and quantitative trait loci at each CNVR. Notably, the database provides sequence read alignments at each CNVR genotype and the boundaries of constituent CNVs in individual samples. Besides numerous novel discoveries, we corroborated the genotypes reported for a CNVR at the *KIT* locus known to be associated with the piebald coat colour phenotype in Hereford and some Simmental cattle.

Conclusions

We present a large comprehensive collection of taurine cattle CNVs in a novel interactive visual database that displays CNV boundaries, read depths and genome features for individual CNVRs, thus providing users with a powerful means to explore and scrutinise CNVRs of interest more thoroughly.

INTRODUCTION

Structural variants (SVs), originally defined to include insertions, deletions and inversions greater than 1 kilobase (Kb) in size [1], now encompass events as small as 50 base pairs (bp) [2]; this change in definition is likely due, in part, to developments in sequencing technology that greatly improved the resolution of discovery achievable. Copy number variants (CNVs) are a class of unbalanced structural variants characterised by changes to the number of base pairs in the genome and manifested as gains or losses of regions of genomic sequence between individuals of a species; CNVs therefore contribute to genetic diversity. Several examples have been reported of CNVs associated with normal variation, disease, evolution and adaptive traits in human, animal and plant species [3–7]. With next-generation sequencing (NGS) technology becoming more cost-effective, traditional methods for CNV discovery that involved hybridisation-based microarray approaches like array comparative genomic hybridisation (array CGH) and SNP microarrays are now being replaced by powerful sequencing-based computational approaches.

Studies on CNV discovery and characterisation have been performed on several farm animal species [8–14] with the ultimate objective of using variants that are associated with traits of economic importance in genetic improvement programs. In cattle, several studies [15–29] have been conducted, in both taurine and indicine breeds, using a variety of algorithms to identify thousands of CNVs. While attempts have been made to provide overall assessments on the reliability of CNV regions (CNVRs) reported in some of those studies using approaches like parent-offspring trios [9], PCR [8] or a combination of *in silico* and experimental techniques [29], the majority have been limited to providing the CNVR boundaries alone. Assessing the potential impact of CNVRs at individual and population levels becomes difficult in the absence of genotypes and boundaries of CNVs constituting CNVRs in individual samples. A recent study [30] has proposed the use of BAM confirmation (i.e. visually examining read depth and read pairing characteristics) as a strategy to assess the accuracy of

predicted CNVRs. This approach was then applied to a limited number of CNVs selected based on overlap with certain human disease-associated genes [30]. Couldrey et al. [31] illustrated the use of long-read sequence information combined with a CNV transmission-based approach to confirm a subset of CNVs that segregate in the New Zealand dairy cattle population. Briefly, the putative CNVs discovered from long-read sequence information in a prominent Holstein-Friesian bull used in New Zealand were first compared with those discovered from short-read sequences in the same bull. Next, a population of 556 cattle representing the wider New Zealand dairy cattle population were short-read sequenced and genotyped at those putative CNV regions, followed by a genome-wide assessment of transmission level of copy number based on pedigree. Visual assessment of highly transmissible CNV regions provided additional evidence to support the presence of CNV across the sequenced animals. Currently, the high cost of long-read sequencing limits adoption of this approach to large numbers of animals representing different breeds, and other studies that provide supportive evidence on a genome-wide scale to help assess the quality of CNVs predicted from short-read sequencing or SNP array data are extremely limited.

The objectives of the present study were to identify and characterise genome-wide CNVRs among popular taurine cattle (*Bos taurus*) breeds and to present the results in a comprehensive interactive database of CNVRs and copy number genotypes, integrated with visualisations of sequence read alignments and genome features. Briefly, CNVs were identified in each of four available whole genome sequence (WGS) datasets, which together represented 553 bulls from 17 different breeds (one dairy and 16 beef breeds). We used cn.MOPS [32], a popular CNV detection software that employs a multi-sample read-depth based algorithm to estimate copy number genotypes per sample. Custom software was then used to convert the results for each dataset into an interactive visual database, a first of its kind for genome-wide CNVR data in any species. The databases, which can be downloaded and then opened using a modern web browser, give users the ability to assess each CNVR with supportive evidence and multiple levels of genome annotation. Further advantages of this format include, for

example, the ability to adjust filtering criteria, compare CNV boundaries and genotypes across samples, and search for affected genes or regions of interest.

RESULTS

Adverse influence of batch effects on CNV discovery from combined datasets

We obtained WGS data on a total of 553 bulls from four different sources; all were paired-end sequenced but differed in the sequencing platform used as well as the coverage, read length, sample size and breed representation (Table 1). Detailed information on samples and breed code translations are provided in the Supplemental Table S1 online. Dataset A was generated using the SOLiD platform and had lower read length and mean coverage (Supplemental Figure S1) than datasets generated using the Illumina platform.

Using aligned sequence data from all bulls simultaneously as input into cn.MOPS, we assessed counts of reads aligned to each non-overlapping window across the genome. The window length (WL) was chosen such that each segment comprised on average 100 reads, as is recommended in cn.MOPS documentation. A WL of 1000 bp satisfied this criterion for datasets A-C. For uniformity, we chose to keep the same WL for dataset D, despite the fact that it had substantially greater sequencing coverage (Table 1) and would have allowed for a lower WL. The CNV discovery algorithm implemented in cn.MOPS derives its power from modelling read count variability across samples, and therefore read count normalisation was performed as a prerequisite. A principal component analysis (PCA) on the normalised read counts per segment across samples revealed clear separation amongst datasets, which was indicative of uncorrected batch effects (Figure 1a). Proceeding with CNV discovery and genotype characterisation using those read counts from all datasets together (after excluding the four PCA outliers) revealed considerable differences in the distribution of CNV genotypes per dataset (Figure 1b). The genotype distributions were skewed towards deletion type (DEL) CNVs in datasets A

and B (datasets with comparatively lower read lengths) as opposed to datasets C and D where the distributions were skewed towards amplification (AMP) type CNVs. These aberrations may arise from the presence of more regions of limited or no coverage in datasets A and B, which triggered false DEL type CNV genotype calls when compared across corresponding regions in other datasets with adequate coverage due to longer read length or advances in sequencing technology. Together, these results indicated the necessity to analyse distinct datasets individually with additional dataset-specific filters applied to identify and remove outlier samples.

Table 1. Sequencing and sample characteristics per dataset

Dataset (year sequenced)	Platform (read length)	Coverage mean (SD)	Total samples	Breed codes * (Number of samples)
A (2012-13)	SOLiD 5500xl (75x35 bp)	7X (4.6)	85	SIM(30), LIM(28), CHA(16), BBR(8), GVH(3)
B (2013-14)	Illumina HiSeq 2000 (100 bp)	11.6X (3.3)	298	HOL(48), AAN(47), SIM(35), HER(33), GVH(28), RAN(26), CHA(25), BBR(16), XXX(14), PIE(7), RDP(7), LIM(6), HYB(3), BAQ(1), DEV(1), SAL(1)
C (2016)	Illumina HiSeq X (150 bp)	10.3X (2.6)	138	CHA(42), LIM(30), SIM(27), AAN(15), HER(15), BBL(9)
D (2017)	Illumina HiSeq X Ten (150 bp)	37.9X (3.6)	32	HOL(32)

* The breed codes used for purebred cattle follow the guidelines provided by the International Committee for Animal Recording (ICAR) for identification of semen straws for international trade. In

addition, XXX represents crossbred cattle and HYB represents composite breeds other than Beef Booster (BBR).

Distributions of CNV genotypes were more consistent across datasets that were analysed individually

To avoid the adverse influence of batch effects on CNV discovery with cn.MOPS when combining datasets with genomic regions of imbalanced coverage, we analysed each dataset individually. Using cn.MOPS, CNVs were identified after first excluding the four PCA outliers (3 in dataset A and 1 in dataset B; see Figure 1b) and three samples within dataset A that were of substantially higher coverage than the others within that dataset (Supplemental Figure S1). Contrary to what was observed when datasets were combined, the proportions of DELs among CNVs were quite consistent among datasets analysed individually (Figure 2), with the mean proportion of DELs ranging between 0.55 (SD 0.08) for dataset D and 0.61 (SD 0.09) for dataset B. Additional quality control (QC) steps were applied to identify problematic samples, defined as those that showed marked deviations (i.e., 1.5 times the interquartile range away from the first and third quartiles) in the proportion of DELs or total CNVs discovered within each dataset. The total number of problematic samples identified were 7, 10, 7 and 3 respectively for datasets A to D. For dataset A, most of the problematic samples identified were amongst the lowest coverage samples (coverage below 5X) while for the other datasets with higher coverage, such a trend was not clearly evident. Plots per dataset that indicate the proportion of the different CNV genotypes identified per sample, distributions of CNV genotype counts, proportion of DELs among CNVs and total CNVs discovered are provided in Supplemental Figures S2-S5 online with problematic samples labelled. All CNVs called within problematic samples were removed which improved the consistency among datasets, with means of the proportion of DELs ranging between 0.57 (SD 0.06) for dataset C and 0.60 (SD 0.07) for dataset B. The CNVs, from the 519 samples that remained after QC, were used to construct CNVRs per dataset based on a 50% reciprocal overlap criterion, consistent with the procedure used elsewhere [26,29]. Finally, refined sets of CNVRs were

obtained after filtering out CNVRs observed in only one sample per dataset. Based on the genotypes of constituent CNVs, the CNVRs were categorised as DEL (CN0/CN1), AMP (CN3+) or mixed (MIX) type (one or more of CN0/CN1 and CN3+). Dataset-wise hierarchical clustering of samples based on the CNVR genotypes (representative genotype of CNVs comprising each CNVR; see Methods) revealed clear clustering by breeds (Supplemental Figures S6-S9 online) as expected.

A list of CNVRs discovered in each dataset with the respective CNVR category assignments is provided in Supplemental Table S2 online. The list consists of a total of 26223 unique CNVRs, counting those with identical genomic coordinates across datasets only once. The dataset-wise counts of CNVs and CNVRs and the non-redundant genome length covered by CNVRs (Table 2) were all proportional to the sample sizes of the individual datasets. These relationships were as expected and were also observed at the breed level (breed-wise summaries of CNVRs are provided in Supplemental Table S3 online). Notably, dataset B had the greatest number of CNVRs in total, which may be attributed to its larger sample size and diversity of breeds, which included purebreds, crossbreds and composites. Conversely, dataset D had the lowest genome coverage by CNVRs, which may be attributed to the fact that it comprised only one breed and thus less genomic variability compared to the other datasets with multiple breeds. These differences amongst datasets were also reflected in the chromosome-wise counts of total CNVRs of each category where datasets of larger sample size and breed diversity revealed higher proportions of MIX category CNVRs (Supplemental Figure S10 a-d online; lower panel). Chromosomes 12, 15, 14 and 29 had comparatively higher density of CNVRs (CNVR counts per megabase (Mb) over the third quartile in all datasets) than others whereas chromosomes 2, 11, 13, 24 and 22 were amongst the least dense (Supplemental Figure S10 a-d online; upper panel). Phenograms representing the chromosomal locations of CNVRs belonging to the different categories indicate distinct patterns broadly conserved across datasets (Supplemental Figure S11 a-d online).

Table 2. Dataset-wise summary of CNVs and CNVRs

Dataset	Number (No.)				Size (Kb) of largest CNVR	Non-redundant size of genome (Mb) covered by CNVRs (%)
	Samples post-QC (No. pre-QC)	CNVs post-QC (No. pre-QC)	CNVRs post-QC (No. pre-QC)	CNVRs per category (No. of DELs; AMPs; MIX)		
A	72 (79)	35531 (41673)	6864 (11625)	2012; 2660; 2192	378	53.8543 (2.02)
B	287 (297)	103040 (117104)	10928 (19139)	2687; 4646; 3595	950	92.48615 (3.48)
C	131 (138)	54797 (61050)	8056 (12351)	2522; 2793; 2741	501	65.90313 (2.48)
D	29 (32)	17790 (20107)	5749 (8988)	1911; 1845; 1993	580	44.47765 (1.67)
Overall summary	519 (546)	157862 (182355)	26223 (44836)	9974; 8302; 9115	950	107.7467 (4.05)

* For the overall summary, the non-redundant size of genome covered was obtained by merging overlapping or adjacent CNVRs across datasets whereas the numbers of CNVs, CNVRs and CNVRs per category were obtained by counting CNVRs with unique genomic coordinates.

Overlaps between CNVRs identified in the four datasets were low when compared to those reported in previous studies but high between the datasets themselves

Previous studies that compared CNVRs discovered across studies reported low percentage of overlap which is attributable to the numerous differences among studies in sample size and characteristics, sequencing platform and technology and CNV detection algorithm, among others. In cattle, the

percentage of overlap among CNVRs discovered across multiple studies was generally below 40% [3,33], with overlapping CNVRs defined as those that share at least one base position. In agreement, the percentage of overlap between the CNVRs detected in 4 datasets of the present study and those detected in previous studies were generally low, ranging between 22 and 35% on average (Table 3). A merged list of CNVRs from the 4 datasets consisted of 9482 CNVRs (mean CNVR size 11.363 Kb; largest CNVR size 3.152 Mb), of which, on average, 37% overlapped with the CNVRs identified in previous studies (Table 3; ABCD). The list was generated by merging overlapping or adjacent CNVRs across datasets as was performed earlier to determine the overall non-redundant size of genome covered by CNVRs (see Table 2). Surprisingly, in another comparison limited to the four datasets, between 70 and 92% of the CNVRs detected in the smaller datasets (A, C and D) overlapped with CNVRs in dataset B, the dataset with the largest sample size and breed representation (Figure 3). Despite the differences amongst the four datasets, the high degree of overlap between CVNRs identified could point to the choice of the CNV detection algorithm being the factor that contributes most to variability in CNVs discovered across studies.

Table 3. Overlaps between CNVRs identified in this study with those from previous published reports

Study	Platform	Nr. chr.	Nr. breeds, samples and CNVRs	% overlap with CNVRs identified in this study				
				A	B	C	D	ABCD
Fadista et al. [15]	CGH-based	29+X	4; 20; 266	12	16.9	13.9	11.3	18
Liu et al. [16]		29+X	17; 90; 223	65.5	78	71.7	57.4	78.9
Hou et al. [17]	SNP-based (50K chip)	29	21; 521; 743	35.8	48	35.1	30.6	51.1
Bae et al. [18] *		29	1; 265; 224	16.5	29	14.3	10.3	33.9
Hou et al. [19]		29	1; 472; 500	21	31.8	21	16.6	35.6
Jiang et al. [20]		22	1; 2047; 64	31.2	48.4	25	21.9	48.4
Hou et al. [21]	SNP-based (HD chip)	29	27; 674; 3438	19.4	28.4	20.5	15.4	33
Wu et al. [22]		29+X	1; 792; 263	38.8	49.8	39.2	29.3	54.4
Bickhart et al. [23]	Whole genome sequencing (WGS)	29	3; 5; 763	10.6	14.4	11.1	9.3	16
Zhan et al. [24]		29	1; 1; 419	8.1	11.5	8.4	9.5	13.8
Stothard et al. [25]		26	2; 2; 634	12.3	15.1	13.2	11.7	16.2
Keel et al. [26]		29+X	7; 154; 1341	60.8	66.4	64	56.3	67.2
Chen et al. [27]		29+X	2; 316; 16325	6.7	10.7	8.1	5.5	12.2
Mean % overlap				26.05	34.49	26.58	21.93	36.82
Nr. of breeds, samples and CNVRs identified in this study				5; 72; 6864	16; 287; 10928	6; 131; 8056	1; 29; 5749	17; 517; 9482

* For studies that used the BTAU 4.0 assembly for mapping, we used the UCSC liftOver tool (https://genome.ucsc.edu/cgi-bin/hgLiftOver) to convert the genomic coordinates of the CNVRs to UMD 3.1.

Identification and genotyping of the well-characterised *KIT* locus CNV in our datasets

A CNVR at Chr6:71747001-71752000, found approximately 45 Kb upstream of the *KIT* gene, (Chr6:71796318-71917431) has been reported to be associated with the piebald coat colour phenotype in HER and some SIM cattle [34–36], but not the dorsal spotting on SIM and HOL cattle or the white patterning on Rouge des Prés [36] (RDP; formerly called Maine-anjou). As one of the few breed-associated cattle CNVs with available genotypes described in the literature we looked at whether our analysis produced consistent breed specificity and genotypes at the *KIT* locus CNVR. Overall, we found (Figure 4) high copy numbers (mostly CN8) in most HER and moderate to high copy numbers in some SIM animals (mostly CN4) across all datasets. Datasets A and B also consisted of a very limited number of a composite breed or crossbreds with moderate copy numbers at the *KIT* locus CNVR, which is likely as those animals may have had SIM or HER animals in their pedigree. Surprisingly, in dataset B (Figure 4b), were 3 CHA with unexpectedly high CN genotypes and 1 HER with CN2 (30 of the 31 HER cattle with non-CN2 genotypes are depicted in the figure). Furthermore, 2 of those 3 CHA clustered with HER and the CN2 genotype HER clustered with CHA in the hierarchical clustering performed based on genome-wide CNVR genotypes (Supplemental Figure S7 online). In an earlier study [37], a PCA of dataset B samples based on their SNP genotypes revealed cross-clustering of the same 3 samples, which was attributed to potential issues with sourcing or handling of those samples. Similarly, in dataset C were an AAN and 2 LIM animals that showed CN8 genotype and clustered with the HER animals while 5 HER animals showed CN2 genotype but did not cluster with the rest of the HER animals in the hierarchical clustering performed based on genome-wide CNVR genotypes (Supplemental Figure S7 online). Manual inspection of the BAM files for those animals at the *KIT* locus CNVR indicated that the read coverages were in agreement with the genotypes predicted by cn.MOPS.

Finally, as expected, the *KIT* locus CNVR was not detected in dataset D which consisted exclusively of HOL animals. Another CVNR, approximately 15 Kb in size (Chr6:71810000-71825000) and located within intron 1 of the *KIT* gene, has been reported to be associated with the piebald coat color [36] In our analysis, the only CNVR that overlaps with this region and that shows amplification in the majority of HER and some SIM animals is an 11 Kb CNVR at Chr6:71808000-71819000, identified only in dataset B. This CNVR was detected in 25 of the 31 HER (24 as CN3 and 1 as CN8) and 7 of the 34 SIM (all as CN3) individuals in dataset B. Thus based on our results, the CNVR at Chr6:71747001-71752000 (upstream of the *KIT* gene) is more clearly associated with the piebald coat color.

An interactive visual database of CNVRs in taurine cattle

Studies of CNVs usually report CNVR positions but rarely the individual genotypes or the boundaries of constituent CNVs in individual samples, or supportive evidence at the level of individual CNVRs. Here we provide in-depth characterisation of CNVRs and present the results in a comprehensive interactive database integrated with visualisations of sequence read alignments, CNV boundaries, and genome features that can be viewed in a modern web browser (for best results, use a recent version of Google Chrome or Mozilla Firefox). In doing so, our strategy better aligns with how we believe the CNVR data will be used: to investigate genome regions of interest for evidence of CNVs and to assess each CNVR with available supportive evidence. The key features of this database are represented in Figure 5 using the *KIT* locus CNVR in dataset B as an example. An index page includes overall summary statistics on CNVRs, as well as custom filtering options for CNVRs and samples. Individual CNVRs are linked to detailed reports that provide a summary of the CNVR, graphs of CNVR genotypes per sample and breed and visual representations of genome features (i.e., gaps, repeats and segmental duplications), genes, QTLs, and CNVs overlapping the CNVR. To determine genes that overlap with CNVRs, we also considered the 5 Mb regions flanking the gene boundaries as part of the gene. Additionally, a link to the NCBI Genome Data Viewer (www.ncbi.nlm.nih.gov/genome/gdv/) [38] plots the CNVR region in the context of the latest annotations and genomics data available in NCBI for UMD3.1.1 bovine reference

genome assembly. Using the viewer, the user can, for example, examine how RNA-Seq data from a variety of tissues aligns with the region, which in turn can help to establish the presence or absence of transcribed regions in the vicinity of the CNVR. One of the most powerful and unique features of the CNVR database is the ability to view raw read alignments as images generated using the Integrative Genomics Viewer (IGV) [39,40]. Images are provided for a random selection of up to three representative samples for each genotype, enabling assessment of the validity of the CNV genotypes and refinement of the CNV boundaries. Furthermore, for autosomal CNVRs, information is provided for tests on parity and Hardy-Weinberg equilibrium (HWE) of the CNVR genotypes. The majority of autosomal CNVRs (97% for datasets A-C; 91% for dataset D) passed the parity test (i.e. the combined frequencies of the heterozygote classes did not exceed that of the homozygote classes). Of the diallelic autosomal CNVRs that qualified for the HWE test per dataset (53-57% of the total for the 4 datasets; see Methods), the majority (63-88%) had genotype proportions that were in HWE (Chi-squared test p-value $\geq 10^{-5}$). In genome-wide association studies, departures from HWE based on genotypes of SNP markers are considered to indicate genotyping errors, batch effects or population stratification and therefore such markers are typically discarded. HWE results are provided as an additional characteristic / annotation of CNVRs but we caution against filtering CNVRs based on HWE as the test is limited to diallelic autosomal CNVRs and deviations from HWE could reflect inaccurate genotypes for an otherwise true CNVR of interest. The CNVR databases per dataset are available via the GigaDB data repository (http://gigadb.org/).

Exploring the CNVR databases for variants of interest

We demonstrate the use of the CNVR database and the powerful interpretations possible through information on genomic features and visualisation of read coverage at CNVRs. Following the creation of the CNVR database, and obtaining basic statistics and summaries of the CNVRs detected in each dataset, we analysed the database for CNVRs that span well-annotated genes and found several thousand CNVRs that partially or completely overlap genes in the four datasets. For example, with

default filters for CNVR length (minimum 1 Kb and maximum 3 Mb) and number of samples in which the CNVR is detected (n=2), typing 'cds del' in the search box of the 'Overlapping Genes' panel for database A indicates 195 entries where a DEL type CNVR overlaps specifically with the coding sequence (CDS) of one or more genes (Supplemental Figure S12 a). Most of those CNVRs also overlap with other components of a gene like the untranslated region (UTR) or intron, or even extend further upstream or downstream of the gene (see column 'Overlap Type' in the 'Overlapping Genes' panel). Selecting the DEL-type CNVR Chr11:6754001-6757000 that overlaps with the interleukin 1 receptor type 2 gene (*IL1R2*) for a detailed view (Supplemental Figure S12 b) indicates that the CNVR passed the parity test but was not in HWE for genotype proportions. As discussed in the previous section, deviations from HWE should not be used as a criterion to filter CNVRs; instead visualisation of the read coverage and other supporting information at the CNVR available through the CNVR database will help validate the predicted CNVs. The selected CNVR was detected in five samples, of which four were of CN0 and one of CN1 genotype ('Summary' and 'Genotypes' panel). Further, the 'Overlaps' panel indicates that the CNV in each of the 5 samples overlaps completely with the penultimate exon and extends to the introns on either side of that exon of *IL1R2*, based on the Ensembl annotation of the gene. Viewing the affected region in the NCBI Genome Data Viewer (using the link provided in the report) corroborates the Ensembl gene model and provides additional support via RNA-Seq exon coverage data (Supplemental Figure S12 c). The CNVR was also detected in dataset B with a start position 1 Kb upstream and in dataset C with an end position 1 Kb downstream, compared to the coordinates of the CNVR in dataset A. The CNVR was not detected in dataset D which consists only of HOLs, and the breed distribution of the CNVR in dataset B, the only other dataset with HOLs, supports the absence of this CNVR in HOLs (Supplemental Figure S12 d). The coverage maps (Supplemental Figure S12 e) reveal red-coloured reads at the boundaries of the CNVR, indicative of a larger than expected insert size, which is a hallmark of deletions. The coverage maps may also suggest potential genotyping errors by cn.MOPs. For example, in dataset C, the sample assigned CN1 appears, based on the absence of coverage over much of the CNVR, to be CN0. The genotyping may have gone wrong

in this case because the end position of that CNVR was wrongly predicted to extend by over one window length into a region of read coverage, which may have affected the calculation of average coverage across the CNVR while assigning the genotype. The ability to view the read coverage maps at the CNVR also enables the refining of the actual boundaries of the CNVR. CNV detection software that utilise read-depth based algorithms for CNV detection usually require a detection window size defined according to the average depth of sequencing (1 Kb window in the current analysis), and report CNVR boundaries at the resolution of the window size. A potential improvement that could be made to the cn.MOPS algorithm is to programmatically resolve the CNVR boundaries to a higher resolution in cases where the read coverage at the CNVR allows it, thereby also improving genotype prediction. In the case of the CNVR within *IL1R2*, analysing the coverage maps helps to exclude the penultimate exon of that gene as being part of the CNVR, as the map shows evidence of read coverage in all samples and datasets at that exon; therefore, the CNVR is actually limited to the intron. Thus, visualisation helps to more precisely assess the potential impacts of the structural variants. It is important to note, however, that intronic CNVRs can affect phenotypes, for example as reported for the *Pea-comb* phenotype in chickens [41]. Another interesting gene where we detected separate intronic CNVRs covering two different introns of the gene across all datasets was calpastatin (*CAST*), wherein multiple SNPs associated with meat tenderness have been reported in beef cattle [42–47]. Here too, viewing the coverage map permits higher resolution determination of the CNVR boundaries (Supplemental Figure S13 a; the first of the 2 intronic CNVRs within *CAST*). Further, the presence of coloured reads at the boundaries of the second intronic CNVR within *CAST*, even in samples of non-DEL genotype (Supplemental Figure S13 b), which initially appeared anomalous, could be explained based on information available through the genomic features tracks, specifically assembly gaps of known (N) and unknown (U) sizes in the region of the CNVR boundaries. The colored reads in such cases could be reads spanning the assembly gaps.

Finally, we provide an example where we looked for evidence of CNVRs at a region in the cattle genome that contains an interesting expanded family of lysozyme genes, which function in bacteria digestion in the abomasum [48]. A region of approximately 0.4 Mb on Chr5 between 44.35 and 44.75 Kb encompasses several members of the lysozyme gene family located in tandem (Supplemental Figure S14 a). Exploring the CNVR database for dataset B, we identified 11 CNVRs of AMP or MIX type within the region of the lysozyme family of genes (Supplemental Figure S14 b). This example shows how the visualisation can help better appreciate the diversity of component CNVs in a complex CNVR, with CNVs of differing genotypes occurring within close proximity to each other and sometimes within the same sample (Supplemental Figure S14 c), thus allowing for a better functional assessment.

Next, we provide an example of a breed-specific CNVR. While there were no CNVRs found fixed in all members of a breed, there were several that were only present in 2 or more members of a particular breed and absent in all other breeds. The number of such breed-specific CNVRs found in datasets A, B and C (dataset D has only one breed and hence excluded) varied from none in certain breeds to a few hundred in others (Supplemental Table S4) and were correlated with the number of samples per breed. Since our datasets consisted of only one dairy breed among the 17 breeds in total, the CNVRs found unique to HOL may indicate association with traits selected for in dairy cattle in general. For example, the CNVR, Chr11:78885001-78891000, was found to be one of the most frequent breed-specific CNVRs in HOL, found in 11 of the 48 HOL in dataset B (all DEL) and 20 of the 32 HOL in dataset D (7 DEL, 13 AMP). Exploring this CNVR in the databases for datasets B (Supplemental Figure S15 a) and D (Supplemental Figure S15 b), the two datasets that consisted of HOL, indicated that the coverage maps from IGV support the CNVR genotypes and the red-coloured reads at the boundaries of the CN0 and CN1 genotype CNVRs further suggest a true deletion. The CNVR overlaps a known QTL for body weight (weaning) and the first exon of the Ensembl model for gene *MATN3*. Further exploration of the gene region via the link to the NCBI Genome Data Viewer (Supplemental Figure S15 c) indicates the following: the CNVR is upstream of the NCBI model of *MATN3* and there is no evidence of RNA-Seq

exon coverage at the region of the first exon in the Ensembl model of *MATN3*. This absence of evidence of transcription could indicate that either the Ensembl model is not accurate or that the samples that contributed to the RNA-Seq data presented in the NCBI Genome Data Viewer were collected from a tissue or stage in life where the first exon of the gene was not transcribed. A previous study [49] identified a CNVR of almost identical coordinates (Chr11:78884928-78891111, "BovineCNV3591") using Genome STRiP software [50] on WGS data from 22 Hanwoo (a Korean breed raised for beef) and 10 HOL breeds. The study reported that the CNVR had a higher deletion frequency in HOL compared to Hanwoo and indicated that the gene *MATN3* was also identified through their analysis of selective sweep signals based on fixation index (F_{ST}) values for measures of population differentiation.

Visualisation of the read coverages at CNVRs can also help identify potential false positive calls by cn.MOPS especially in regions of low sequencing coverage. In the case of the CNVRs depicted in Supplemental Figure S16, the low coverage is clearly attributable to the numerous assembly gaps at the region. Setting a higher threshold for coverage and removing CNVRs detected within a certain distance from a known assembly gap may help resolve some of these cases at the expense of some loss of true positive CNVRs. In the future, we plan to implement a filter that examines consistency of coverage across the window, allowing for deviations at the ends, to better identify and remove such cases.

The above examples, together with the example of the CNVR at the *KIT* gene locus described earlier (Figure 4 and 5), demonstrate the value of the CNVR databases created in this study. The data summaries, visualisation of gene features, CNV genotypes, CNVR boundaries and read coverages at CNVRs, serve as powerful tools to ascertain the veracity and potential phenotype-altering mechanisms of CNVRs, as well as the prevalence of individual CNV genotypes among breeds and in the populations studied.

Discussion

With the ever-reducing costs, WGS has become the method of choice for many applications involving CNV detection. Software to predict CNVs has also evolved and methods that rely on multi-sample read-depth analyses, like cn.MOPS, have become popular due to their superior ability to control for false discovery rate [32]. Furthermore, a recent study on simulated data has reported read depth based approaches to perform relatively better than those based on paired end and split read analyses when analyzing datasets comprised of samples sequenced at varying levels of coverage [26]. Using cn.MOPS, we analysed each of four WGS datasets which together represent over 500 bulls from 17 taurine cattle breeds. Besides CNV detection, cn.MOPS provides integer copy number genotypes to indicate the level of deletion or amplification at the predicted CNVs. We did not use the built-in function within cn.MOPS to construct CNVRs and assign CNVR genotypes as we found that this approach can produce very large CNVRs which obscure the underlying breakpoint diversity across samples and that have genotype assignments that are not always consistent with the majority genotype observed among the constituent CNVs. We therefore employed a 50% pairwise reciprocal overlap criterion to construct CNVRs, as has been used in other studies [26,29] and then assigned genotypes based on a set of rules as described in the Methods section. The assigned CNVR genotypes indicated clear separation of breeds by hierarchical clustering and also confirmed previously reported differences in the amplification levels at the *KIT* locus CNVR between Simmental and Hereford breeds. In future work, individual CNVR genotypes could be used in association analyses aimed at investigating the relationship between copy number and phenotype. In addition, we provide detailed annotation including sequencing read coverage for each CNVR in multiple samples representing the different genotypes identified. All results are presented in a unique interactive visual database which enables the user to assess each CNVR based on sequence read alignments and to examine the boundaries of constituent CNVs in individual samples. Read coverage and alignments within and adjacent to a CNVR can aid in the determination of the breakpoints of constituent CNVs in individual samples, as the resolution of the

breakpoints reported by the cn.MOPS algorithm is limited to the choice of window size used for CNV detection. The visualisation of genome features like assembly gaps and repeats can highlight potential non-CNV related coverage and alignment anomalies, and thus can further be used in the assessment of predicted CNVs and their breakpoints. We believe that the way we present our results in the CNVR database better aligns with how this information will be used, that is, to investigate genomic regions or genes of interest for evidence of CNVs; such information is not available at a genome-wide scale in any of the previously published reports on CNVRs in any species.

An important outcome from the present study was the necessity to address batch effects that could affect the reliability of CNVs predicted using algorithms that model read count variations across samples. The batch effects arise from genomic regions of imbalanced coverage across sequence datasets generated from different platforms and technologies. While the batch effects could potentially be controlled to an extent by including only those genomic regions that have adequate coverage across datasets, such an approach would have resulted in losing valuable information on CNVRs from individual datasets that had sufficient coverage at those regions. These observations guided our decision to analyse individual datasets separately.

One limitation of the present study was that some of the breeds had low sample representation; the PIE, RDP, and BBL breeds had less than 10 samples each while the BAQ, DEV and SAL breeds had only 1 sample each. Therefore, the breadth of breed-specific CNVRs reported is not as complete for those breeds as are those for the more popular breeds with greater sample representation in the present study. Nevertheless, CNVRs in some of those breeds with smaller representation (for example, DEV, SAL, BBL) have not been studied or reported earlier at a genome-wide scale, making this study amongst the first to do so in those breeds. Another limitation of the present study is that CNVRs shorter than 3000 bp are not reported, which was the limit we set for the dataset-wise analyses based on the sequencing coverage of samples in the dataset with the lowest mean coverage.

To conclude, this study presents a comprehensive collection of CNVRs in taurine cattle, which can serve as a reference on the locations of CNVRs and their genotype frequencies in a broad range of cattle breeds. The visualisations and annotations included in the interactive databases greatly facilitate assessment of individual CNVRs and should aid the efforts to identify CNVRs that influence phenotype. We recommend that visualisation of read coverage at predicted CNVRs be a standard protocol in studies reporting specific CNVRs of interest (for example near to a gene or genome region highlighted through some other research activities) among CNVRs identified on a genome-wide scale. Given the issue of false positive calls inherent to any prediction algorithm and the impracticality of experimental validation for CNVRs at a genome-wide scale, read coverage visualisation at CNVRs offers a powerful way to not only overcome those issues but also to refine the CNVR boundaries, among other advantages. Further, we suggest integrating the NCBI Genome Data Viewer into analysis workflows as a way of assessing the NCBI and Ensembl gene models and their supporting evidence (for example, RNA-Seq reads) when examining how CNVRs overlap with genome features.

Methods

Sequence data

The WGS datasets were generated in four different projects which together comprised 553 samples representing 1 taurine dairy cattle breed and 16 taurine beef cattle breeds (Table1 and Supplemental Table S1 online). The sequence data were generated following guidelines provided by the 1000 bull genomes project (http://www.1000bullgenomes.com/) [51]. Details on animal selection, sequence generation and further analyses performed on datasets A and B have been published earlier [37,51]. Briefly, DNA samples were extracted from commercial artificial insemination bull semen straws and sequenced using either the 5500xl SOLiD™ system (85 animals) or the HiSeq™ 2000 system (298 animals). Reads that passed standard quality-based filtering criteria were aligned to the UMD3.1 bovine reference genome assembly [52] using *BWA-backtrack* algorithm of Burrows-Wheeler Aligner (BWA)

[53] version 0.5.9. Local realignment of reads around indels was performed using *IndelRealigner* tool of the Genome Analysis Toolkit (GATK) [54] version 2.4, and duplicate reads marked using *MarkDuplicates* tool of the Picard toolkit version 1.54 (http://broadinstitute.github.io/picard/). Details on animal selection, sequence generation and further analyses performed on datasets C and D were similar to those for the previous datasets except for using more recent versions of the following software: BWA version 0.7.15 for dataset C and version 0.7.12 for dataset D, both using *BWA-MEM* algorithm, GATK version 3.5 and Picard toolkit version 2.0.1.

Identification of CNVs from sequence data

Detection of CNVs in the sequence data was performed using the Bioconductor [55] (version 3.6) package cn.MOPS [32] (version 1.24.0) of R (version 3.4.3) statistical programming language [56] running on a CentOS 7 Linux server with default cn.MOPS parameters except the following: *WL* 1000 bp and *rmdup* enabled to count only one read for each unique combination of position, strand and read width. CNVs were reported if 3 adjacent windows show significant read depth variations, thereby enabling the detection of CNVs of length 3000 bp and higher in increments of 1000 bp.

Constructing CNVRs from CNVs

In cn.MOPS, CNVRs are constructed from CNVs by merging overlapping and adjacent CNVs using the *reduce* function from the Bioconductor package “GenomicRanges”. An initial test run on dataset A using that approach resulted in abnormally large CNVRs. Hence we followed a more conservative approach to merge CNVs to CNVRs similar to what was used in some previous studies [26,29] in which CNVRs were constructed by merging only those CNVs across samples that satisfied a 50% pairwise reciprocal overlap criteria based on their genomic coordinates.

Assigning genotypes to CNVRs

By default, cn.MOPS assigns CNVR genotypes for each sample based on the genotypes of the CNVs comprising each CNVR. While the default approach worked well for the majority of cases, the selected genotype was not representative for 2.37 to 6.18% of the CNVRs across datasets where multiple discrete CNVs of differing genotypes occurred in certain individual samples. Such cases were observed more frequently for larger CNVRs. To assign CNVR genotypes, we used the genotype of the CNV type with the largest aggregate width amongst all CNV types comprising the CNVR; in case of ties, we assigned the genotype that was closer to CN2. The corrected genotypes were used to perform genotype-based hierarchical clustering of samples (using the *hclust* function in R with the *Spearman* correlation based distance measure and the *ward.D2* agglomeration method). Another issue with genotype assignment to CNVRs is associated with the 50% reciprocal overlap criterion that allows creation of overlapping CNVRs. In general, a CN2 genotype is assigned to samples where a CNV is not detected in a particular CNVRs; however, it is possible that the same sample may have a CNV of non-CN2 genotype detected on an overlapping CNVR. Therefore, we performed a CN2 correction as follows: for each test CNVR, the genotypes of samples for which cn.MOPS did not detect a CNV were changed from the default CN2 to CN_ in cases where a CNV was detected for that sample in another CNVR that overlapped with the test CNVR. The genotypes subsequently obtained were used for all summary calculations and plots created in the CNVR database.

Annotation of CNVRs

The CNVRs were annotated for genes based on information obtained from Ensembl [57,58] Release 88 (Bos_taurus.UMD3.1.88.gff3) and for cattle QTLs (99,652 QTLs) from Animal QTLdb [59] Release 33 (Aug 26, 2017) [https://www.animalgenome.org/cgi-bin/QTLdb/BT/index]. Information on segmental duplications in bovines was retrieved from sheet 1 of additional file 3 (Table S3.1-7) of a previous study [60] whereas assembly gaps and repeats were obtained for Bos_taurus_UMD_3.1/bosTau6 (Nov. 2009) assembly UCSC genome table browser (https://genome.ucsc.edu/cgi-bin/hgTables).

Hardy–Weinberg equilibrium (HWE) test on CNVR genotypes

We performed Pearson's chi-squared tests for goodness of fit of CNVR genotype proportions to HWE [61] at diallelic autosomal CNVRs with either a combination of CN0, CN1 and CN2 genotypes (considered as minor allele homozygous, heterozygous, and reference homozygous) or CN2, CN3 and CN4 genotypes (considered as reference homozygous, heterozygous, and minor allele homozygous), similar to a previous study [62]. The test was performed using the "HardyWeinberg" package [63] in R. Multi-allelic CNVR genotypes were not tested for HWE here because of the inability to determine what combination of alleles were responsible for a particular genotype. Furthermore, at all autosomal CNVRs, a parity test [64] was performed to test whether the number of individuals that have even CNVR genotypes (CN0, CN2, CN4 and CN8) exceed the number of individuals with odd CNVR genotypes (CN1, CN3, CN5 and CN7), an extension of the observation in SNP genotypes that, at HWE, the combined frequencies of the homozygote classes should exceed that of the heterozygote classes).

AVAILABILITY OF DATA AND MATERIALS

All data generated during this study are included in the article or as Supplemental files online. Raw sequence data for datasets A, B, C and D have been deposited to public databases (Sequence Read Archive (SRA) accessions SRP017441, SRP044884, SRP150844 and SRP153409 respectively). In addition, aligned sequence data for datasets A and B are available at GigaDB dataset ID 100157 (http://dx.doi.org/10.5524/100157). The CNVR databases per dataset are available via the GigaDB data repository (http://gigadb.org/).

ACKNOWLEDGMENTS

This research was supported by funding from Genome Canada, Genome Alberta, and Science Foundation Ireland (SFI) principal investigator award grant number 14/IA/2576 as well as a research grant from Science Foundation Ireland and the Department of Agriculture, Food and Marine on behalf

of the Government of Ireland under the Grant 16/RC/3835 (VistaMilk). The analyses were performed, in part, using computing resources provided by WestGrid (http://www.westgrid.ca), Compute Canada (http://www.computecanada.ca) and Cybera (https://www.cybera.ca/).

AUTHOR CONTRIBUTIONS

PS and CFB designed the study. CFB, AB and DPB oversaw sample selection, acquisition and sequencing. AK, KK, AB and TRC performed sequence analysis and/or CNV detection. JRG developed the interactive CNV database. AK performed CNVR identification and downstream analyses steps and drafted the manuscript. All authors read, revised and approved the manuscript.

COMPETING INTERESTS

The authors declare that they have no competing interests in the manuscript.

REFERENCES

1. Feuk L, Carson AR, Scherer SW. Structural variation in the human genome. Nat Rev Genet. Nature Publishing Group; 2006;7:85–97.

2. Sudmant PH, Rausch T, Gardner EJ, Handsaker RE, Abyzov A, Huddleston J, et al. An integrated map of structural variation in 2,504 human genomes. Nature. Nature Publishing Group; 2015;526:75–81.

3. Keel BN, Lindholm-Perry AK, Snelling WM. Evolutionary and Functional Features of Copy Number Variation in the Cattle Genome. Front Genet. Frontiers Media SA; 2016;7:207.

4. Canales CP, Walz K. Copy number variation and susceptibility to complex traits. EMBO Mol Med. Wiley-Blackwell; 2011;3:1–4.

5. Zarrei M, MacDonald JR, Merico D, Scherer SW. A copy number variation map of the human genome. Nat Rev Genet. Nature Publishing Group; 2015;16:172–83.

6. Prunier J, Caron | S Ebastien, Lamothe | Manuel, Blais | Sylvie, Bousquet J, Isabel N, et al. Gene copy number variations in adaptive evolution: The genomic distribution of gene copy number variations revealed by genetic mapping and their adaptive role in an undomesticated species, white spruce (Picea glauca). 2017;

7. Ricard G, Molina J, Chrast J, Gu W, Gheldof N, Pradervand S, et al. Phenotypic consequences of copy number variation: insights from Smith-Magenis and Potocki-Lupski syndrome mouse models. PLoS Biol. Public Library of Science; 2010;8:e1000543.

8. Fadista J, Nygaard M, Holm L-E, Thomsen B, Bendixen C. A Snapshot of CNVs in the Pig Genome. Kroymann J, editor. PLoS One. Public Library of Science; 2008;3:e3916.

9. Ramayo-Caldas Y, Castelló A, Pena RN, Alves E, Mercadé A, Souza CA, et al. Copy number variation in the porcine genome inferred from a 60 k SNP BeadChip. BMC Genomics. BioMed Central; 2010;11:593.

10. Paudel Y, Madsen O, Megens H-J, Frantz LA, Bosse M, Bastiaansen JW, et al. Evolutionary dynamics of copy number variation in pig genomes in the context of adaptation and domestication. BMC Genomics. BioMed Central; 2013;14:449.

11. Crooijmans RP, Fife MS, Fitzgerald TW, Strickland S, Cheng HH, Kaiser P, et al. Large scale variation in DNA copy number in chicken breeds. BMC Genomics. BioMed Central; 2013;14:398.

12. Yi G, Qu L, Liu J, Yan Y, Xu G, Yang N. Genome-wide patterns of copy number variation in the diversified chicken genomes using next-generation sequencing. BMC Genomics. BioMed Central; 2014;15:962.

13. Fontanesi L, Martelli P, Beretti F, Riggio V, Dall'Olio S, Colombo M, et al. An initial comparative map of copy number variations in the goat (Capra hircus) genome. BMC Genomics. BioMed Central; 2010;11:639.

14. Chen C, Qiao R, Wei R, Guo Y, Ai H, Ma J, et al. A comprehensive survey of copy number variation

in 18 diverse pig populations and identification of candidate copy number variable genes associated with complex traits. BMC Genomics. BioMed Central; 2012;13:733.

15. Fadista J, Thomsen B, Holm L-E, Bendixen C. Copy number variation in the bovine genome. BMC Genomics. BioMed Central; 2010;11:284.

16. Liu GE, Hou Y, Zhu B, Cardone MF, Jiang L, Cellamare A, et al. Analysis of copy number variations among diverse cattle breeds. Genome Res. 2010;20:693–703.

17. Hou Y, Liu GE, Bickhart DM, Cardone MF, Wang K, Kim E, et al. Genomic characteristics of cattle copy number variations. BMC Genomics. BioMed Central; 2011;12:127.

18. Bae J, Cheong H, Kim L, NamGung S, Park T, Chun J-Y, et al. Identification of copy number variations and common deletion polymorphisms in cattle. BMC Genomics. BioMed Central; 2010;11:232.

19. Hou Y, Liu GE, Bickhart DM, Matukumalli LK, Li C, Song J, et al. Genomic regions showing copy number variations associate with resistance or susceptibility to gastrointestinal nematodes in Angus cattle. Funct Integr Genomics. 2012;12:81–92.

20. Jiang L, Jiang J, Wang J, Ding X, Liu J, Zhang Q. Genome-Wide Identification of Copy Number Variations in Chinese Holstein. Watson M, editor. PLoS One. Public Library of Science; 2012;7:e48732.

21. Hou Y, Bickhart DM, Hvinden ML, Li C, Song J, Boichard DA, et al. Fine mapping of copy number variations on two cattle genome assemblies using high density SNP array. BMC Genomics. BioMed Central; 2012;13:376.

22. Wu Y, Fan H, Jing S, Xia J, Chen Y, Zhang L, et al. A genome-wide scan for copy number variations using high-density single nucleotide polymorphism array in Simmental cattle. Anim Genet. Wiley/Blackwell (10.1111); 2015;46:289–98.

23. Bickhart DM, Hou Y, Schroeder SG, Alkan C, Cardone MF, Matukumalli LK, et al. Copy number

variation of individual cattle genomes using next-generation sequencing. Genome Res. 2012;22:778–90.

24. Zhan B, Fadista J, Thomsen B, Hedegaard J, Panitz F, Bendixen C. Global assessment of genomic variation in cattle by genome resequencing and high-throughput genotyping. BMC Genomics. BioMed Central; 2011;12:557.

25. Stothard P, Choi J-W, Basu U, Sumner-Thomson JM, Meng Y, Liao X, et al. Whole genome resequencing of black Angus and Holstein cattle for SNP and CNV discovery. BMC Genomics. BioMed Central; 2011;12:559.

26. Keel BN, Keele JW, Snelling WM. Genome-wide copy number variation in the bovine genome detected using low coverage sequence of popular beef breeds. Anim Genet. 2017;48:141–50.

27. Chen L, Chamberlain AJ, Reich CM, Daetwyler HD, Hayes BJ. Detection and validation of structural variations in bovine whole-genome sequence data. Genet Sel Evol. BioMed Central; 2017;49:13.

28. Boussaha M, Esquerré D, Barbieri J, Djari A, Pinton A, Letaief R, et al. Genome-wide study of structural variants in bovine Holstein, Montbéliarde and Normande dairy breeds. PLoS One. 2015;10:1–21.

29. Letaief R, Rebours E, Grohs C, Meersseman C, Fritz S, Trouilh L, et al. Identification of copy number variation in French dairy and beef breeds using next-generation sequencing. Genet Sel Evol. BioMed Central; 2017;49:77.

30. Trost B, Walker S, Wang Z, Thiruvahindrapuram B, MacDonald JR, Sung WWL, et al. A Comprehensive Workflow for Read Depth-Based Identification of Copy-Number Variation from Whole-Genome Sequence Data. Am J Hum Genet. Cell Press; 2018;102:142–55.

31. Couldrey C, Keehan M, Johnson T, Tiplady K, Winkelman A, Littlejohn MD, et al. Detection and assessment of copy number variation using PacBio long-read and Illumina sequencing in New Zealand dairy cattle. J Dairy Sci. Elsevier; 2017;100:5472–8.

32. Klambauer G, Schwarzbauer K, Mayr A, Clevert DA, Mitterecker A, Bodenhofer U, et al. Cn.MOPS: Mixture of Poissons for discovering copy number variations in next-generation sequencing data with a low false discovery rate. Nucleic Acids Res. 2012;40:1–14.

33. Keel BN, Keele JW, Snelling WM. Genome-wide copy number variation in the bovine genome detected using low coverage sequence of popular beef breeds,. Anim Genet. 2017;48:141–50.

34. Olson TA. The genetic basis for piebald patterns in cattle. J Hered. Oxford University Press; 1981;72:113–6.

35. Fontanesi L, Tazzoli M, Russo V, Beever J. Genetic heterogeneity at the bovine *KIT* gene in cattle breeds carrying different putative alleles at the *spotting* locus. Anim Genet. Wiley/Blackwell (10.1111); 2010;41:295–303.

36. Whitacre L. Structural variation at the KIT locus is responsible for the piebald phenotype in Hereford and Simmental cattle. University of Missouri-Columbia; 2014.

37. Stothard P, Liao X, Arantes AS, De Pauw M, Coros C, Plastow GS, et al. A large and diverse collection of bovine genome sequences from the Canadian Cattle Genome Project. Gigascience. BioMed Central; 2015;4:49.

38. Agarwala R, Barrett T, Beck J, Benson DA, Bollin C, Bolton E, et al. Database resources of the National Center for Biotechnology Information. Nucleic Acids Res. Oxford University Press; 2018;46:D8–13.

39. Thorvaldsdottir H, Robinson JT, Mesirov JP. Integrative Genomics Viewer (IGV): high-performance genomics data visualization and exploration. Brief Bioinform. Oxford University Press; 2013;14:178–92.

40. Robinson JT, Thorvaldsdóttir H, Winckler W, Guttman M, Lander ES, Getz G, et al. Integrative genomics viewer. Nat Biotechnol. 2011;29:24–6.

41. Wright D, Boije H, Meadows JRS, Bed'hom B, Gourichon D, Vieaud A, et al. Copy Number

Variation in Intron 1 of SOX5 Causes the Pea-comb Phenotype in Chickens. Stern DL, editor. PLoS Genet. Public Library of Science; 2009;5:e1000512.

42. Calvo JH, Iguácel LP, Kirinus JK, Serrano M, Ripoll G, Casasús I, et al. A new single nucleotide polymorphism in the calpastatin (CAST) gene associated with beef tenderness. Meat Sci. 2014;96:775–82.

43. Enriquez-Valencia CE, Pereira GL, Malheiros JM, de Vasconcelos Silva JAII, Albuquerque LG, de Oliveira HN, et al. Effect of the g.98535683A > G SNP in the CAST gene on meat traits of Nellore beef cattle (Bos indicus) and their crosses with Bos taurus. Meat Sci. 2017;123:64–6.

44. Tait RG, Shackelford SD, Wheeler TL, King DA, Casas E, Thallman RM, et al. µ-Calpain, calpastatin, and growth hormone receptor genetic effects on preweaning performance, carcass quality traits, and residual variance of tenderness in Angus cattle selected to increase minor haplotype and allele frequencies1,2,3. J Anim Sci. 2014;92:456–66.

45. Gill JL, Bishop SC, McCorquodale C, Williams JL, Wiener P. Association of selected SNP with carcass and taste panel assessed meat quality traits in a commercial population of Aberdeen Angus-sired beef cattle. Genet Sel Evol. 2009;41:36.

46. Casas E, White SN, Wheeler TL, Shackelford SD, Koohmaraie M, Riley DG, et al. Effects of calpastatin and micro-calpain markers in beef cattle on tenderness traits. J Anim Sci. 2006;84:520–5.

47. Tait RG, Shackelford SD, Wheeler TL, King DA, Keele JW, Casas E, et al. CAPN1, CAST, and DGAT1 genetic effects on preweaning performance, carcass quality traits, and residual variance of tenderness in a beef cattle population selected for haplotype and allele equalization1,2,3,4. J Anim Sci. Oxford University Press; 2014;92:5382–93.

48. Irwin DM. Evolution of the bovine lysozyme gene family: Changes in gene expression and reversion of function. J Mol Evol. Springer-Verlag; 1995;41:299–312.

49. Shin D-H, Lee H-J, Cho S, Kim H, Hwang J, Lee C-K, et al. Deleted copy number variation of

Hanwoo and Holstein using next generation sequencing at the population level. BMC Genomics. BioMed Central; 2014;15:240.

50. Handsaker RE, Korn JM, Nemesh J, McCarroll SA. Discovery and genotyping of genome structural polymorphism by sequencing on a population scale. Nat Genet. Nature Publishing Group; 2011;43:269–76.

51. Daetwyler HD, Capitan A, Pausch H, Stothard P, van Binsbergen R, Brøndum RF, et al. Whole-genome sequencing of 234 bulls facilitates mapping of monogenic and complex traits in cattle. Nat Genet. 2014;46:858–65.

52. Zimin A V, Delcher AL, Florea L, Kelley DR, Schatz MC, Puiu D, et al. A whole-genome assembly of the domestic cow, Bos taurus. Genome Biol. BioMed Central; 2009;10:R42.

53. Li H, Durbin R. Fast and accurate short read alignment with Burrows-Wheeler transform. Bioinformatics. 2009;25:1754–60.

54. McKenna A, Hanna M, Banks E, Sivachenko A, Cibulskis K, Kernytsky A, et al. The Genome Analysis Toolkit: A MapReduce framework for analyzing next-generation DNA sequencing data. Genome Res. 2010;20:1297–303.

55. Gentleman RC, Carey VJ, Bates DM, Bolstad B, Dettling M, Dudoit S, et al. Bioconductor: open software development for computational biology and bioinformatics. Genome Biol. 2004;5:R80.

56. Ihaka R, Gentleman R. R: A Language for Data Analysis and Graphics. J Comput Graph Stat. 1996;5:299–314.

57. Aken BL, Ayling S, Barrell D, Clarke L, Curwen V, Fairley S, et al. The Ensembl gene annotation system. Database. Oxford University Press; 2016;2016:baw093.

58. Yates A, Akanni W, Amode MR, Barrell D, Billis K, Carvalho-Silva D, et al. Ensembl 2016. Nucleic Acids Res. Oxford University Press; 2016;44:D710–6.

59. Hu Z-L, Park CA, Reecy JM. Developmental progress and current status of the Animal QTLdb. Nucleic Acids Res. Oxford University Press; 2016;44:D827–33.

60. Feng X, Jiang J, Padhi A, Ning C, Fu J, Wang A, et al. Characterization of genome-wide segmental duplications reveals a common genomic feature of association with immunity among domestic animals. BMC Genomics. BioMed Central; 2017;18:293.

61. Hardy GH. MENDELIAN PROPORTIONS IN A MIXED POPULATION. Science. American Association for the Advancement of Science; 1908;28:49–50.

62. Mei TS, Salim A, Calza S, Seng KC, Seng CK, Pawitan Y. Identification of recurrent regions of Copy-Number Variants across multiple individuals. BMC Bioinformatics. BioMed Central; 2010;11:147.

63. Graffelman J. Exploring Diallelic Genetic Markers: The **HardyWeinberg** Package. J Stat Softw. 2015;64:1–23.

64. Handsaker RE, Van Doren V, Berman JR, Genovese G, Kashin S, Boettger LM, et al. Large multiallelic copy number variations in humans. Nat Genet. NIH Public Access; 2015;47:296–303.

Figure legends

Figure 1. Batch effects among the 4 datasets contributing to inconsistent distribution of CNV genotypes in the analysis of the combined datasets

(a) PCA based on normalised read counts per segment showed separation by datasets and 4 outliers. (b) When datasets were combined and analysed together using cn.MOPS (N=549 after removing PCA outliers), the distribution of CNV genotypes revealed considerable differences among datasets (only autosomal CNVs are depicted here).

Figure 2. Distributions of CNV genotypes were more consistent across datasets that were analysed individually

When datasets were analysed individually (*N*=546 after removing PCA outliers and high coverage outlier samples in dataset A), the distribution of CNV genotypes were consistent among datasets (only autosomal CNVs are depicted here).

Figure 3. Proportions of overlapping CNVRs amongst datasets.

Pair-wise comparisons of the proportions of CNVRs in each dataset (rows; ordered by dataset size) that overlap by at least one base pair with CNVRs of other larger datasets (columns) are presented.

Figure 4. Prevalence and genotypes of the *KIT* locus CNV across breeds and datasets

The breed-wise prevalence and genotypes at CNVR Chr6:71747001-71752000, found approximately 45 Kb upstream of the *KIT* gene is depicted here. This CNVR has been reported to be associated with the piebald coat colour phenotype in HER and some SIM cattle, and occurs in high copy numbers in these breeds. The reason for detection of this CNVR in high copy number in 2 of the 22 CHA cattle in dataset B is attributed to potential issues with sourcing or handling of the respective samples.

Figure 5. Key features of the functionality of the CNVR database

The database has an index view and a detailed view with an option to enable/disable the help function on the top right of each page. The index page (a) has a panel (**Filters**) that allow users to apply filters to the CNVRs such as CNVR length or the number of samples that must contain the CNVR and the ability to exclude/include specific samples based on regular expression matches. Another panel (**Statistics**) provides summary information on the CNVRs before and after applying the filters. The remaining panels on the index page allow users to search and sort on **CNVRs**, overlapping **genes** and **QTLs** and/or **samples** to quickly find CNVRs associated with a particular gene/QTL. All or selected data can be exported as CSV files. CNVRs of interest can be noted as favorites; and comments can be added for individual CNVRs. All comments, filters and/or favorites can be saved as a text file that can be reloaded later using the **Settings** button options on the top right of the page. Clicking on a CNVR

provides a detailed view (b) with panels displaying basic statistics on the CNVR (**Summary**), a bar plot of the number of samples per CNV genotype (**Genotype distribution**) and another bar plot of the number of non-CN2 variants per breed (**Breed distribution**), graphical representation of the CNVR in genomic context (**Overlapping genes, QTLs and CNVs**), sequence read coverage at the CNVR for up to 3 samples per genotype (**IGV images**), a table of all the samples indicating the CNV genotype (**CNVR-specific sample list**) and finally a **sample view** that provides for the selected sample, a graphical representation of the CNVR and CNV in genomic context with overlapping genes and QTLs.

Additional file legends

Figure S1: Sample-wise sequencing coverages per dataset.

Figure S2-S5: Proportions of the different CNV genotypes identified per sample (a), distributions of CNV genotype counts (b), proportions of DELs among CNVs (c) and total CNVs discovered (d) per dataset.

Figure S6-S9: Hierarchical clustering of samples based on the CNVR genotypes per dataset.

Figure S10: Chromosome-wise counts of total CNVRs and CNVRs per category (DEL, AMP, MIX) for datasets A (a), B (b), C (c) and D (d).

Figure S11: Phenograms representing the chromosomal locations of CNVRs belonging to the different categories for datasets A (a), B (b), C (c) and D (d).

Figure S12-S16: Specific examples to depict exploration of the CNVR databases for variants of interest.

Table S1: Detailed information on samples and breed code translations.

Table S2: List of CNVRs discovered in each dataset with the respective CNVR category assignments.

Table S3: Breed-wise summaries of CNVRs identified per dataset.

Table S4: Breed-specific CNVRs found in datasets A, B and C.

Figure 1

Click here to access/download;Figure;Figure1.pdf

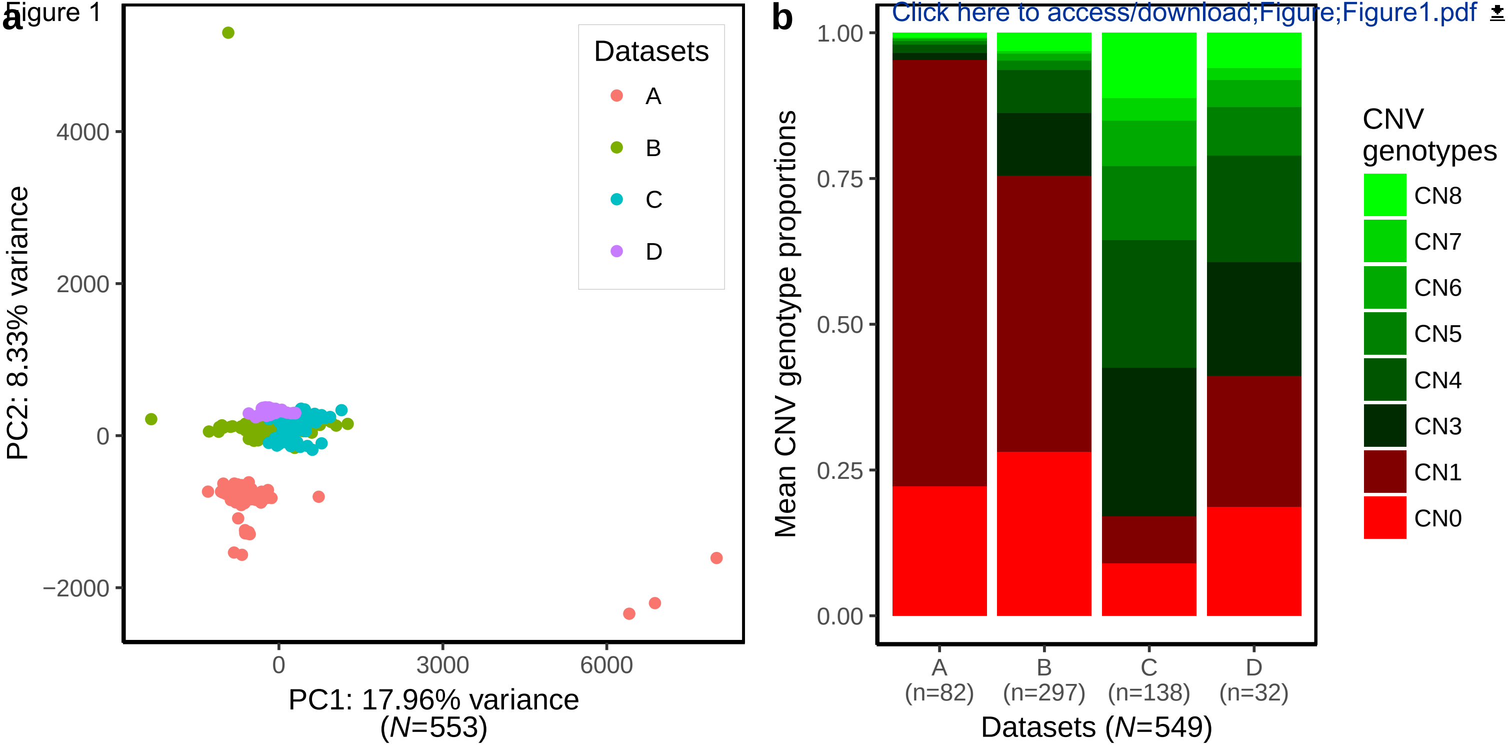

Figure 2

Click here to access/download;Figure;Fig

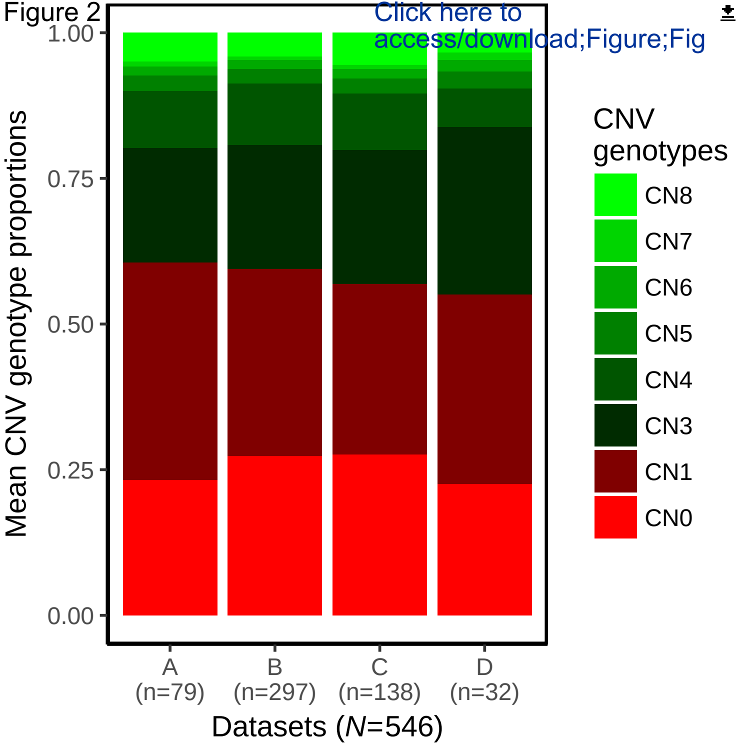

Figure 3

Click here to access/download;Figure;Figure3.pdf

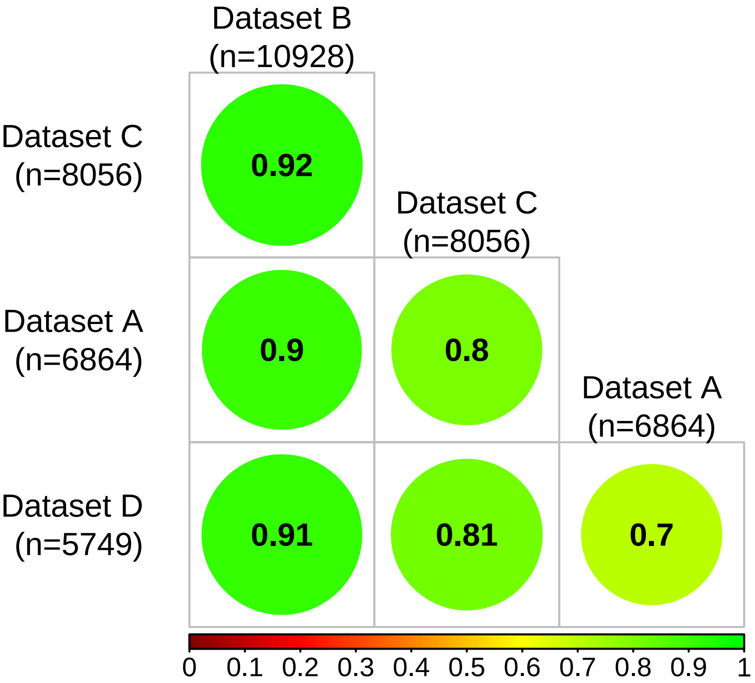

Figure 4

Click here to access/download;Figure;Figure4.pdf

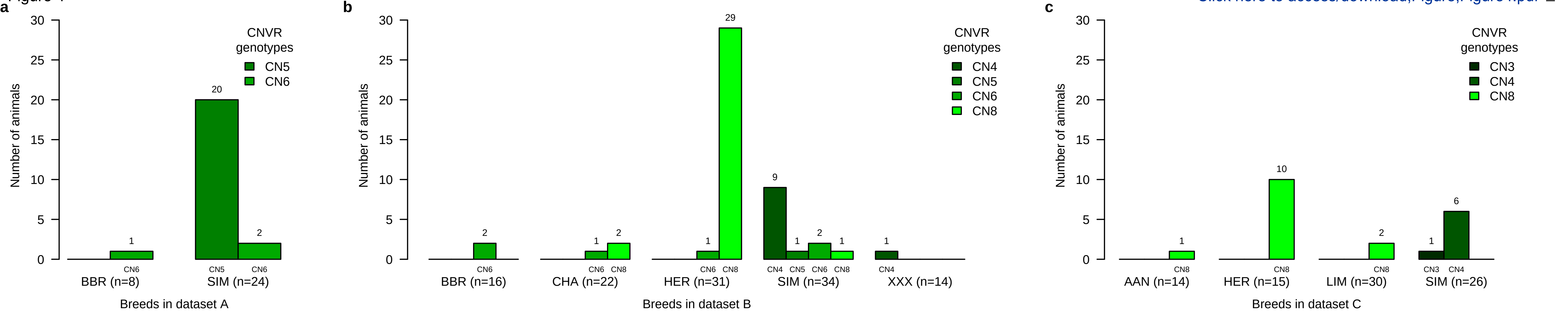

Figure 5

Click here to access/download;Figure;Figure5.png

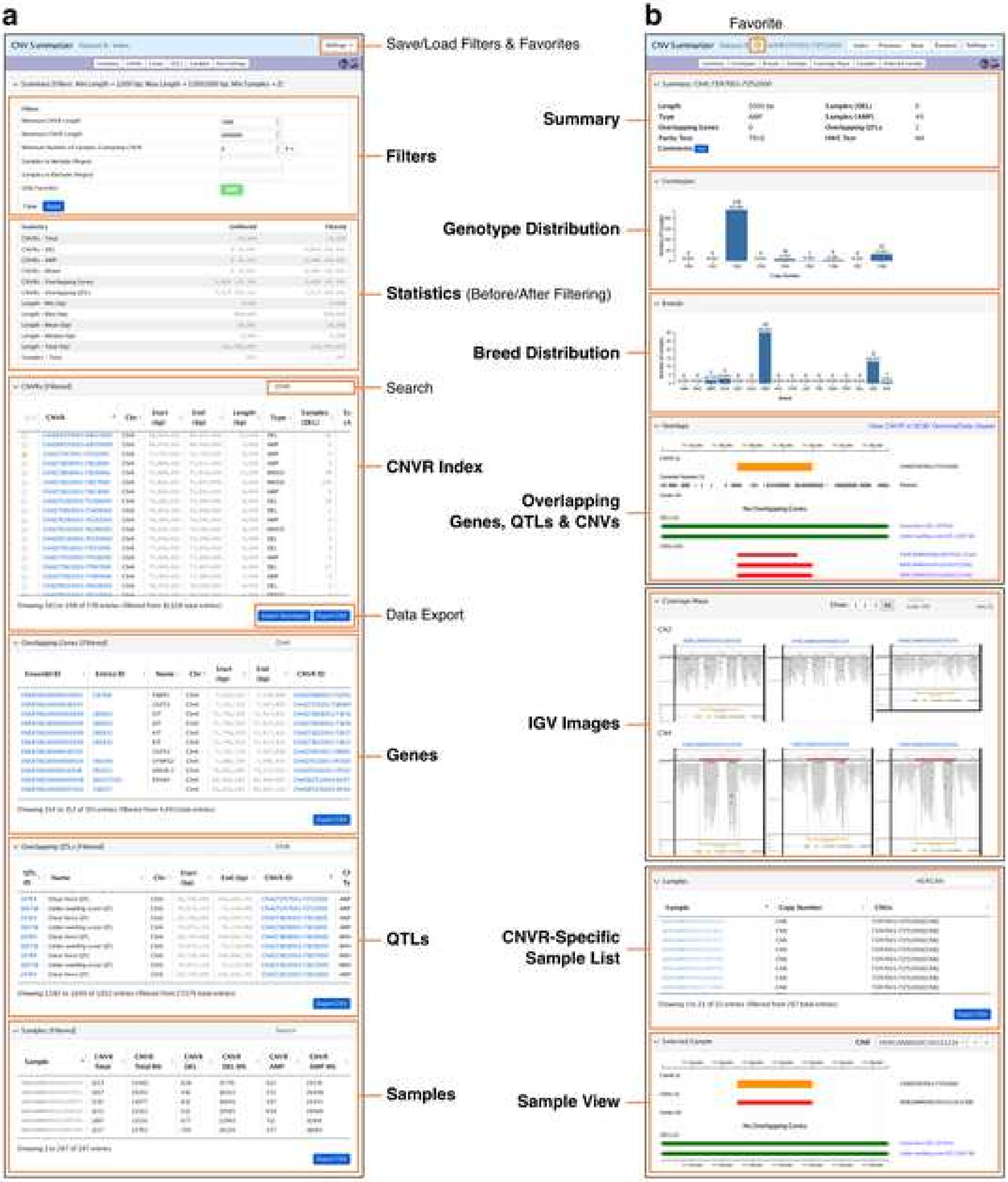

Figure S1

Click here to access/download
Supplementary Material
Supplemental_Figure_S1.pdf

Figure S2

Click here to access/download
Supplementary Material
Supplemental_Figure_S2.pdf

Figure S3

Click here to access/download
Supplementary Material
Supplemental_Figure_S3.pdf

Figure S4

Click here to access/download
Supplementary Material
Supplemental_Figure_S4.pdf

Figure S5

Click here to access/download
Supplementary Material
Supplemental_Figure_S5.pdf

Figure S6

Click here to access/download
Supplementary Material
Supplemental_Figure_S6.pdf

Figure S7

Click here to access/download
Supplementary Material
Supplemental_Figure_S7.pdf

Figure S8

Click here to access/download
Supplementary Material
Supplemental_Figure_S8.pdf

Figure S9

Click here to access/download
Supplementary Material
Supplemental_Figure_S9.pdf

Figure S10

Click here to access/download
Supplementary Material
Supplemental_Figure_S10.pdf

Figure S11

Click here to access/download
Supplementary Material
Supplemental_Figure_S11.pdf

Figure S12

Click here to access/download
Supplementary Material
Supplemental_Figure_S12.pdf

Figure S13

Click here to access/download
Supplementary Material
Supplemental_Figure_S13.pdf

Figure S14

Click here to access/download
Supplementary Material
Supplemental_Figure_S14.pdf

Figure S15

Click here to access/download
Supplementary Material
Supplemental_Figure_S15.pdf

Figure S16

Click here to access/download
Supplementary Material
Supplemental_Figure_S16.pdf

Table S1

Click here to access/download
Supplementary Material
Supplemental_Table_S1.xlsx

Table S2

Click here to access/download
Supplementary Material
Supplemental_Table_S2.xlsx

Table S3

Click here to access/download
Supplementary Material
Supplemental_Table_S3.xlsx

Table S4

Click here to access/download
Supplementary Material
Supplemental_Table_S4.xlsx
