# Supplementary material for: A large interactive visual database of copy number variants discovered in taurine cattle
Source: Gigascience. 2019 Jun 26;8(6):giz073. doi: 10.1093/gigascience/giz073 (PMC6593363; doi:10.1093/gigascience/giz073)
Supplement: giz073_Supplemental_Files [file giz073_supplemental_files.zip › Supplemental_Figure_S10.pdf]

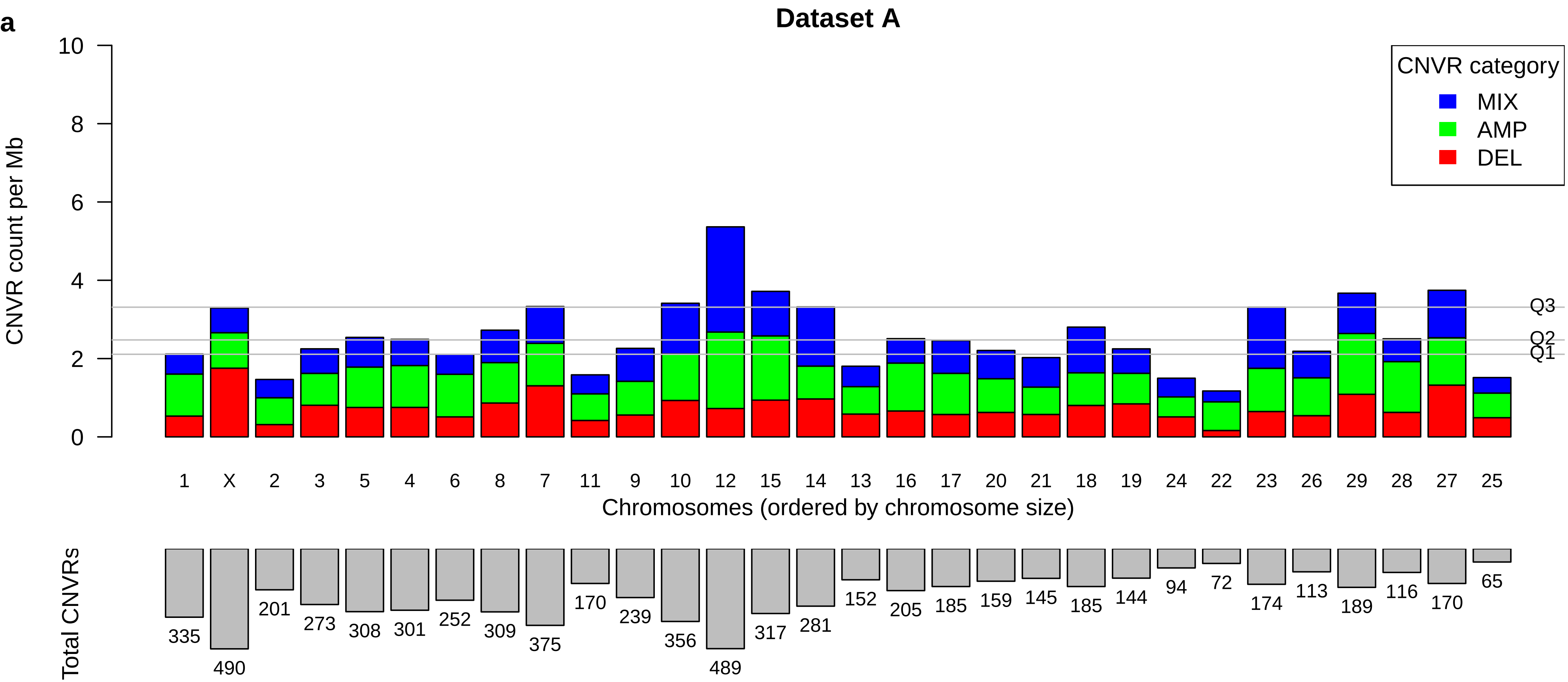

a
Dataset A
10
8
6
4
2
0
CNVR count per Mb
CNVR category
MIX
AMP
DEL
Q3
Q2
Q1
1 X 2 3 5 4 6 8 7 11 9 10 12 15 14 13 16 17 20 21 18 19 24 22 23 26 29 28 27 25
Chromosomes (ordered by chromosome size)
Total CNVRs
335 490 201 273 308 301 252 309 375 170 239 356 489 317 281 152 205 185 159 145 185 144 94 72 174 113 189 116 170 65

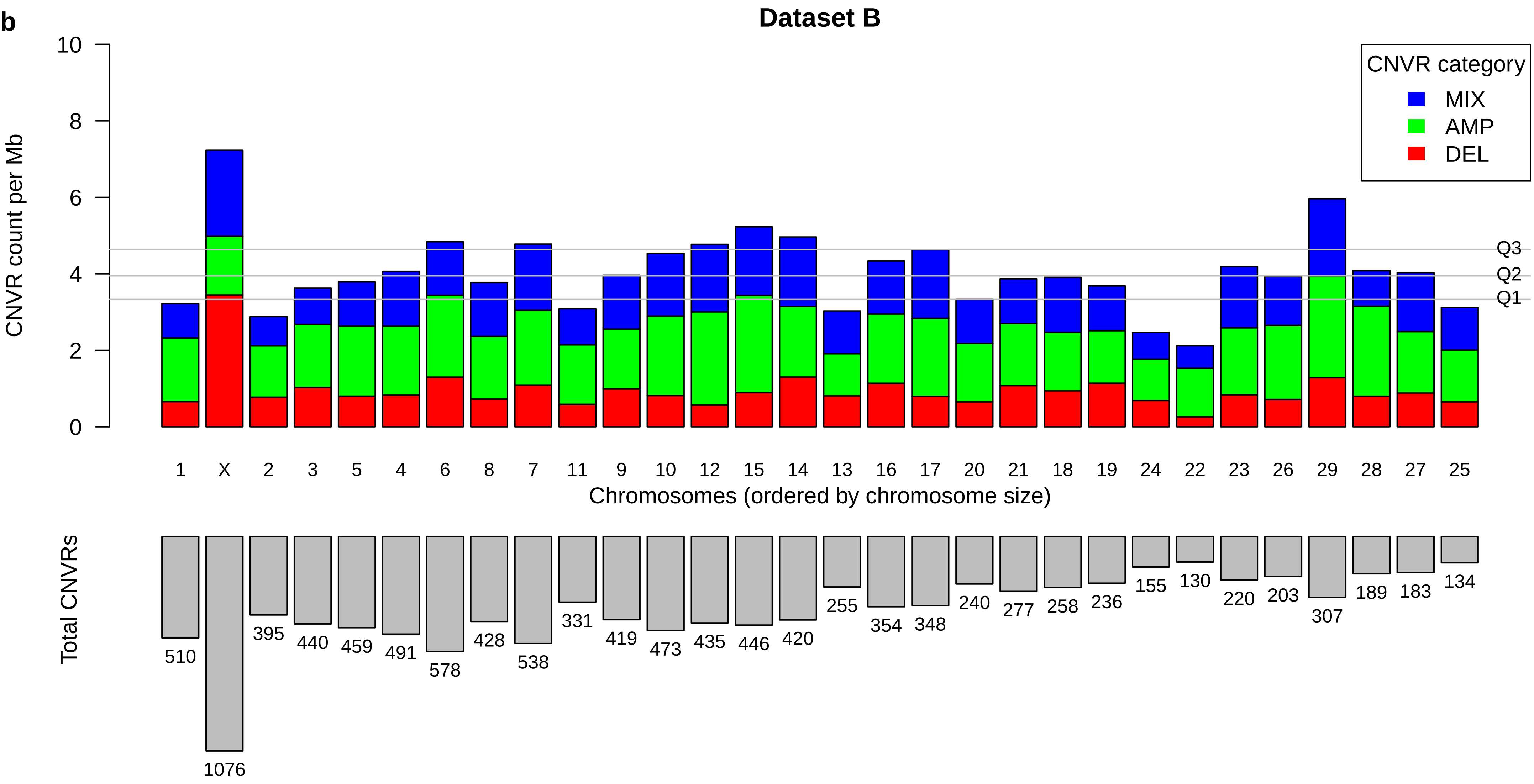

b
Dataset B
CNVR count per Mb
0
2
4
6
8
10
CNVR category
MIX
AMP
DEL
Q3
Q2
Q1
1 X 2 3 5 4 6 8 7 11 9 10 12 15 14 13 16 17 20 21 18 19 24 22 23 26 29 28 27 25
Chromosomes (ordered by chromosome size)
Total CNVRs
510 1076 395 440 459 491 578 428 538 331 419 473 435 446 420 255 354 348 240 277 258 236 155 130 220 203 307 189 183 134

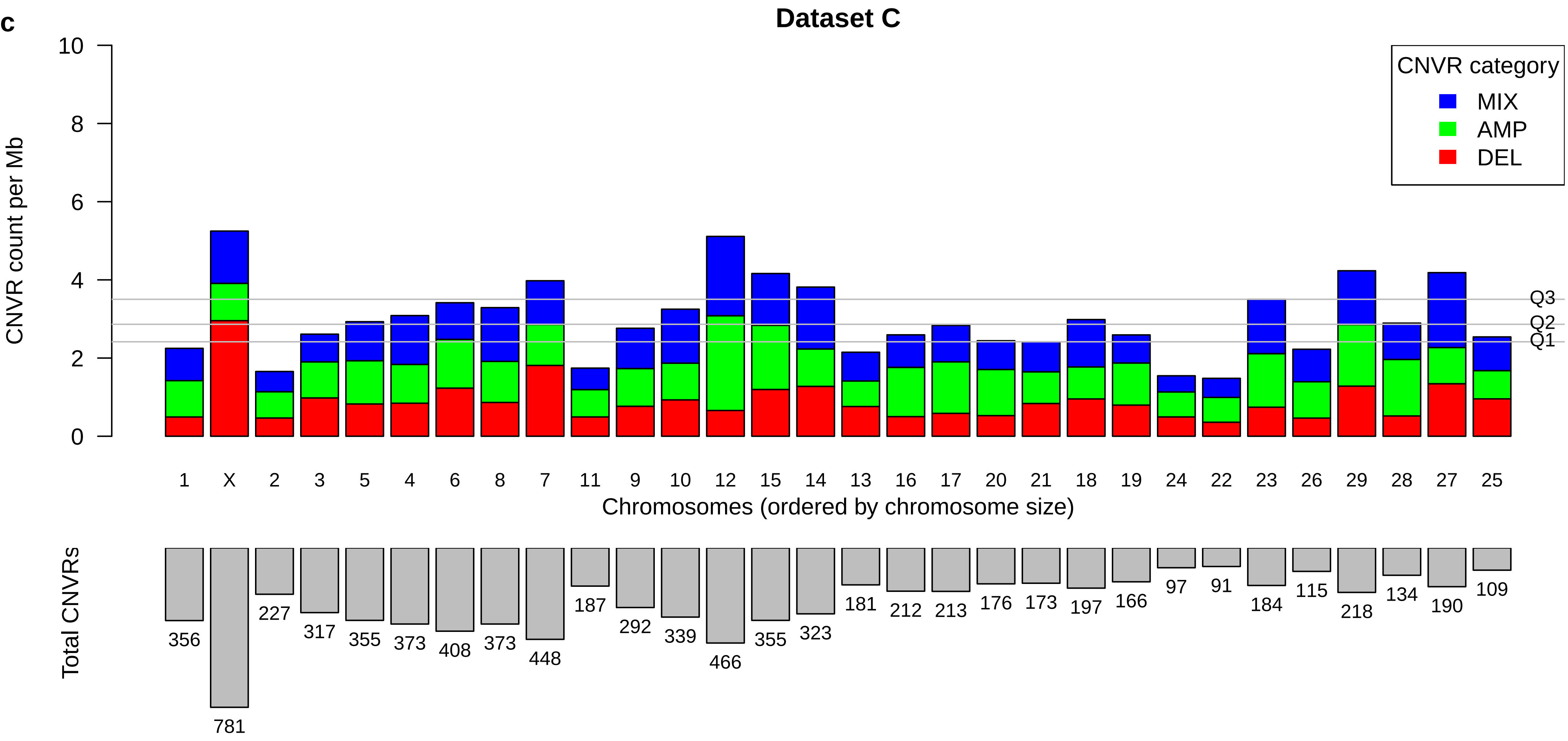

c
Dataset C
CNVR count per Mb
0
2
4
6
8
10
CNVR category
MIX
AMP
DEL
Q3
Q2
Q1
1 X 2 3 5 4 6 8 7 11 9 10 12 15 14 13 16 17 20 21 18 19 24 22 23 26 29 28 27 25
Chromosomes (ordered by chromosome size)
Total CNVRs
356 781 227 317 355 373 408 373 448 187 292 339 466 355 323 181 212 213 176 173 197 166 97 91 184 115 218 134 190 109

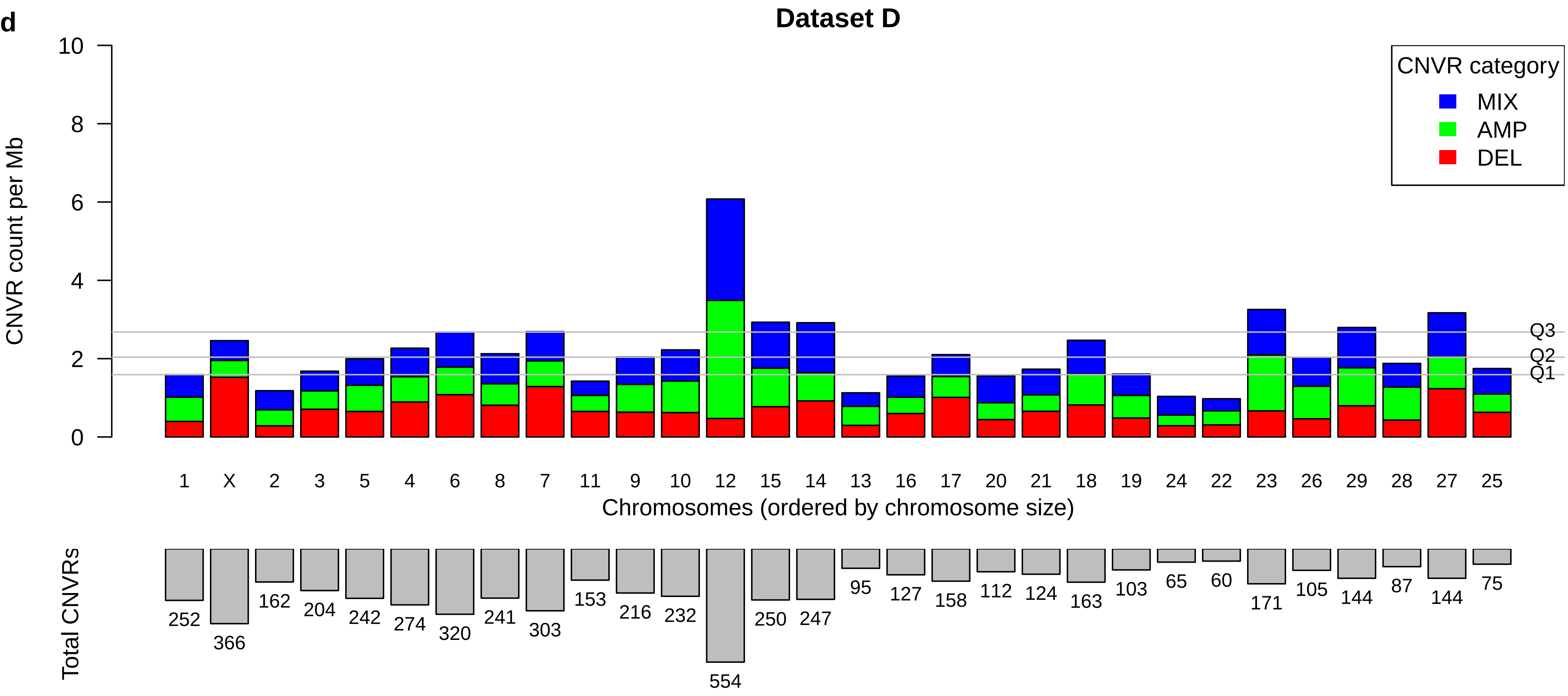
d
Dataset D
CNVR count per Mb
10
8
6
4
2
0
CNVR category
MIX
AMP
DEL
Q3
Q2
Q1
1 X 2 3 5 4 6 8 7 11 9 10 12 15 14 13 16 17 20 21 18 19 24 22 23 26 29 28 27 25
Chromosomes (ordered by chromosome size)
Total CNVRs
252 366 162 204 242 274 320 241 303 153 216 232 554 250 247 95 127 158 112 124 163 103 65 60 171 105 144 87 144 75
