# Supplementary material for: A large interactive visual database of copy number variants discovered in taurine cattle
Source: Gigascience. 2019 Jun 26;8(6):giz073. doi: 10.1093/gigascience/giz073 (PMC6593363; doi:10.1093/gigascience/giz073)
Supplement: giz073_Supplemental_Files [file giz073_supplemental_files.zip › Supplemental_Figure_S12.pdf]

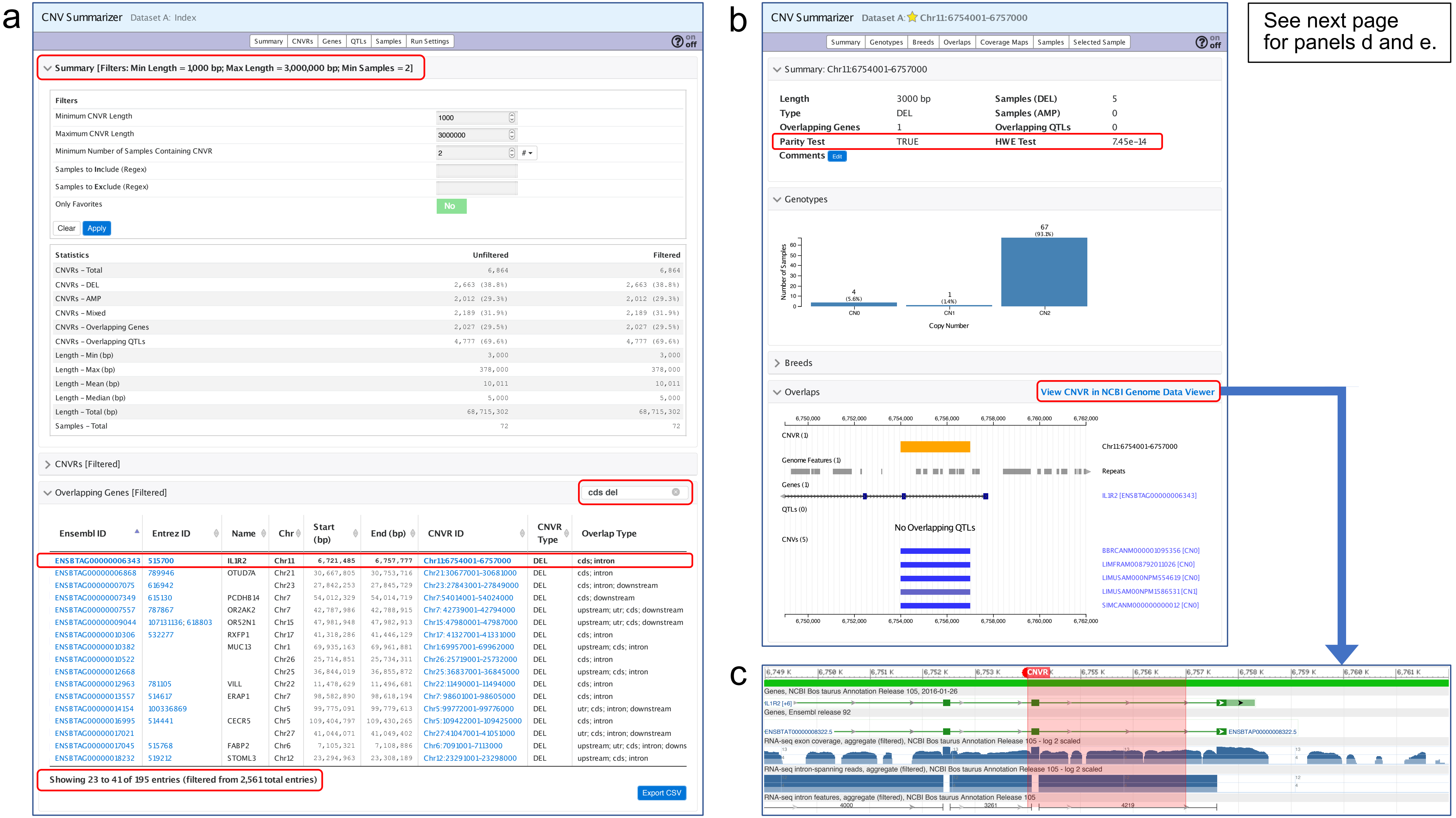
a
CNV Summarizer Dataset A: Index
Summary CNVRs Genes QTLs Samples Run Settings
Summary [Filters: Min Length = 1,000 bp; Max Length = 3,000,000 bp; Min Samples = 2]
Filters
Minimum CNVR Length 1000
Maximum CNVR Length 3000000
Minimum Number of Samples Containing CNVR 2 #
Samples to Include (Regex)
Samples to Exclude (Regex)
Only Favorites No
Clear Apply
Statistics Unfiltered Filtered
CNVRs – Total 6,864 6,864
CNVRs – DEL 2,663 (38.8%) 2,663 (38.8%)
CNVRs – AMP 2,012 (29.3%) 2,012 (29.3%)
CNVRs – Mixed 2,189 (31.9%) 2,189 (31.9%)
CNVRs – Overlapping Genes 2,027 (29.5%) 2,027 (29.5%)
CNVRs – Overlapping QTLs 4,777 (69.6%) 4,777 (69.6%)
Length – Min (bp) 3,000 3,000
Length – Max (bp) 378,000 378,000
Length – Mean (bp) 10,011 10,011
Length – Median (bp) 5,000 5,000
Length – Total (bp) 68,715,302 68,715,302
Samples – Total 72 72
CNVRs [Filtered]
Overlapping Genes [Filtered]
cds del
Ensembl ID | Entrez ID | Name | Chr | Start (bp) | End (bp) | CNVR ID | CNVR Type | Overlap Type
ENSBTAG00000006343 | 515700 | IL1R2 | Chr11 | 6,721,485 | 6,757,777 | Chr11:6754001-6757000 | DEL | cds; intron
ENSBTAG00000006868 | 789946 | OTUD7A | Chr21 | 30,667,805 | 30,753,716 | Chr21:30677001-30681000 | DEL | cds; intron
ENSBTAG00000007075 | 616942 | | Chr23 | 27,842,253 | 27,845,729 | Chr23:27843001-27849000 | DEL | cds; intron; downstream
ENSBTAG00000007349 | 615130 | PCDHB14 | Chr7 | 54,012,329 | 54,014,719 | Chr7:54014001-54024000 | DEL | cds; downstream
ENSBTAG00000007557 | 787867 | OR2AK2 | Chr7 | 42,787,986 | 42,788,915 | Chr7:42739001-42794000 | DEL | upstream; utr; cds; downstream
ENSBTAG00000009044 | 107131136; 618803 | OR52N1 | Chr15 | 47,981,948 | 47,982,913 | Chr15:47980001-47987000 | DEL | upstream; utr; cds; downstream
ENSBTAG00000010306 | 532277 | RXFP1 | Chr17 | 41,318,286 | 41,446,129 | Chr17:41327001-41331000 | DEL | cds; intron
ENSBTAG00000010382 | | MUC13 | Chr1 | 69,935,163 | 69,961,881 | Chr1:69957001-69962000 | DEL | upstream; cds; intron
ENSBTAG00000010522 | | | Chr26 | 25,714,851 | 25,734,311 | Chr26:25719001-25732000 | DEL | cds; intron
ENSBTAG00000012668 | | | Chr25 | 36,844,019 | 36,855,872 | Chr25:36837001-36845000 | DEL | upstream; cds; intron
ENSBTAG00000012963 | 781105 | VILL | Chr22 | 11,478,629 | 11,496,681 | Chr22:11490001-11494000 | DEL | cds; intron
ENSBTAG00000013557 | 514617 | ERAP1 | Chr7 | 98,582,890 | 98,618,194 | Chr7:98601001-98605000 | DEL | cds; intron
ENSBTAG00000014154 | 100336869 | | Chr5 | 99,775,091 | 99,779,613 | Chr5:99772001-99776000 | DEL | utr; cds; intron; downstream
ENSBTAG00000016995 | 514441 | CECR5 | Chr5 | 109,404,797 | 109,430,265 | Chr5:109422001-109425000 | DEL | cds; intron
ENSBTAG00000017021 | | | Chr27 | 41,044,071 | 41,049,402 | Chr27:41047001-41051000 | DEL | utr; cds; intron; downstream
ENSBTAG00000017045 | 515768 | FABP2 | Chr6 | 7,105,321 | 7,108,886 | Chr6:7091001-7113000 | DEL | upstream; utr; cds; intron; downs
ENSBTAG00000018232 | 519212 | STOML3 | Chr12 | 23,294,963 | 23,308,189 | Chr12:23291001-23298000 | DEL | upstream; cds; intron
Showing 23 to 41 of 195 entries (filtered from 2,561 total entries)
Export CSV
b
CNV Summarizer Dataset A: Chr11:6754001-6757000
Summary Genotypes Breeds Overlaps Coverage Maps Samples Selected Sample
Summary: Chr11:6754001-6757000
Length 3000 bp
Type DEL
Overlapping Genes 1
Parity Test TRUE
Comments Edit
Samples (DEL) 5
Samples (AMP) 0
Overlapping QTLs 0
HWE Test 7.45e–14
Genotypes
Number of Samples
Copy Number
CN0 4 (5.6%)
CN1 1 (1.4%)
CN2 67 (93.1%)
Breeds
Overlaps
View CNVR in NCBI Genome Data Viewer
CNVR (1)
Chr11:6754001-6757000
Genome Features (1)
Repeats
Genes (1)
IL1R2 [ENSBTAG00000006343]
QTLs (0)
No Overlapping QTLs
CNVs (5)
BBRCANM000001095356 [CN0]
LIMFRAM008792011026 [CN0]
LIMUSAM000NPM554619 [CN0]
LIMUSAM000NPM1586531 [CN1]
SIMCANM000000000012 [CN0]
c
CNVR
Genes, NCBI Bos taurus Annotation Release 105, 2016-01-26
Genes, Ensembl release 92
RNA-seq exon coverage, aggregate (filtered), NCBI Bos taurus Annotation Release 105 - log 2 scaled
RNA-seq intron-spanning reads, aggregate (filtered), NCBI Bos taurus Annotation Release 105 - log 2 scaled
RNA-seq intron features, aggregate (filtered), NCBI Bos taurus Annotation Release 105
See next page for panels d and e.

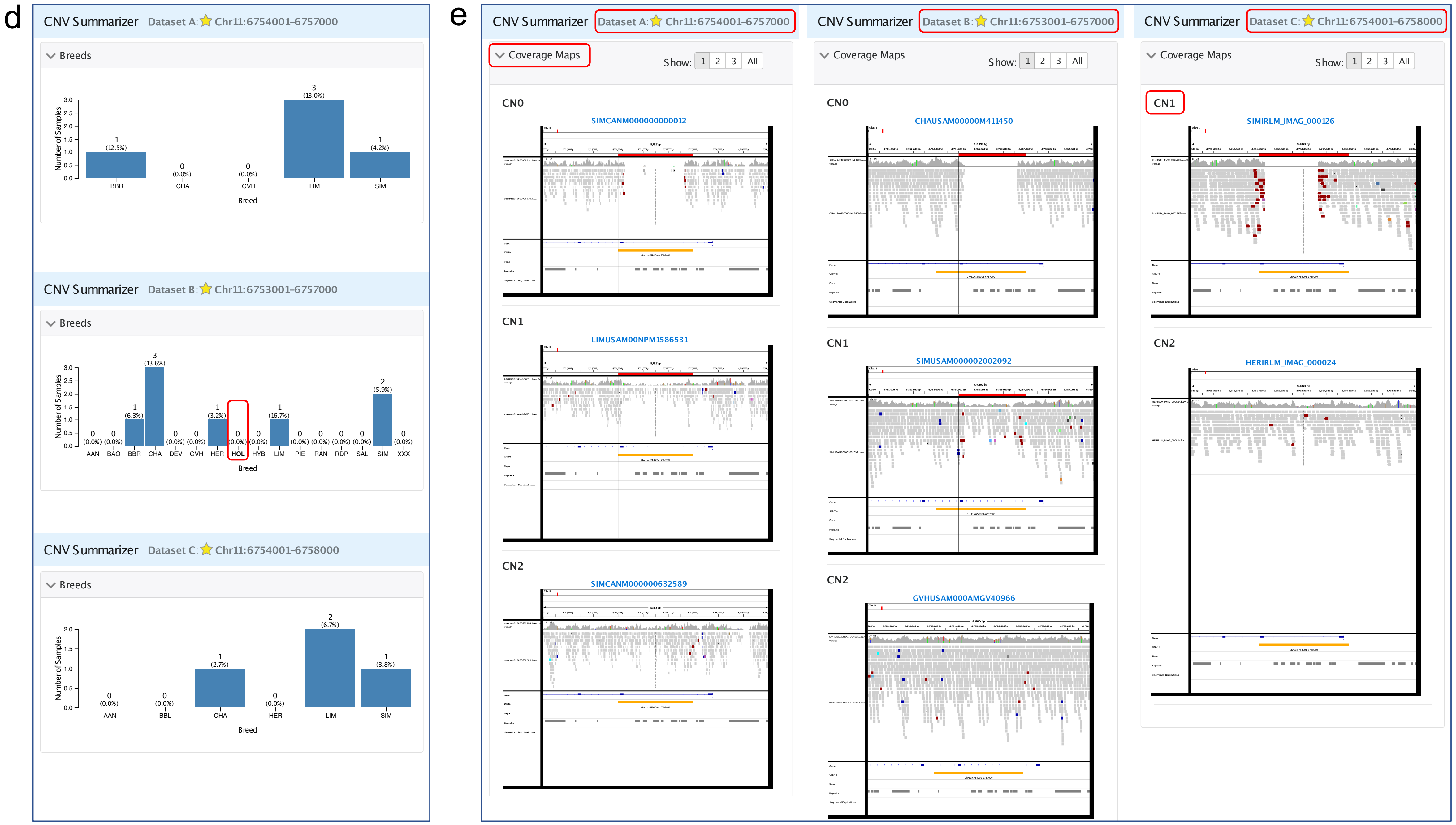

d
CNV Summarizer Dataset A: Chr11:6754001-6757000
Breeds
Number of Samples
3.0 2.5 2.0 1.5 1.0 0.5 0.0
1 (12.5%) BBR
0 (0.0%) CHA
0 (0.0%) GVH
3 (13.0%) LIM
1 (4.2%) SIM
Breed
CNV Summarizer Dataset B: Chr11:6753001-6757000
Breeds
Number of Samples
3.0 2.5 2.0 1.5 1.0 0.5 0.0
0 (0.0%) AAN
0 (0.0%) BAQ
1 (6.3%) BBR
3 (13.6%) CHA
0 (0.0%) DEV
0 (0.0%) GVH
1 (3.2%) HER
0 (0.0%) HOL
0 (0.0%) HYB
1 (16.7%) LIM
0 (0.0%) PIE
0 (0.0%) RAN
0 (0.0%) RDP
0 (0.0%) SAL
2 (5.9%) SIM
0 (0.0%) XXX
Breed
CNV Summarizer Dataset C: Chr11:6754001-6758000
Breeds
Number of Samples
2.0 1.5 1.0 0.5 0.0
0 (0.0%) AAN
0 (0.0%) BBL
1 (2.7%) CHA
0 (0.0%) HER
2 (6.7%) LIM
1 (3.8%) SIM
Breed
e
CNV Summarizer Dataset A: Chr11:6754001-6757000
Coverage Maps
Show: 1 2 3 All
CN0
SIMCANM000000000012
CN1
LIMUSAM00NPM1586531
CN2
SIMCANM000000632589
CNV Summarizer Dataset B: Chr11:6753001-6757000
Coverage Maps
Show: 1 2 3 All
CN0
CHAUSAM00000M411450
CN1
SIMUSAM000002002092
CN2
GVHUSAM000AMGV40966
CNV Summarizer Dataset C: Chr11:6754001-6758000
Coverage Maps
Show: 1 2 3 All
CN1
SIMIRLM_IMAG_000126
CN2
HERIRLM_IMAG_000024
