# Supplementary material for: A large interactive visual database of copy number variants discovered in taurine cattle
Source: Gigascience. 2019 Jun 26;8(6):giz073. doi: 10.1093/gigascience/giz073 (PMC6593363; doi:10.1093/gigascience/giz073)

a

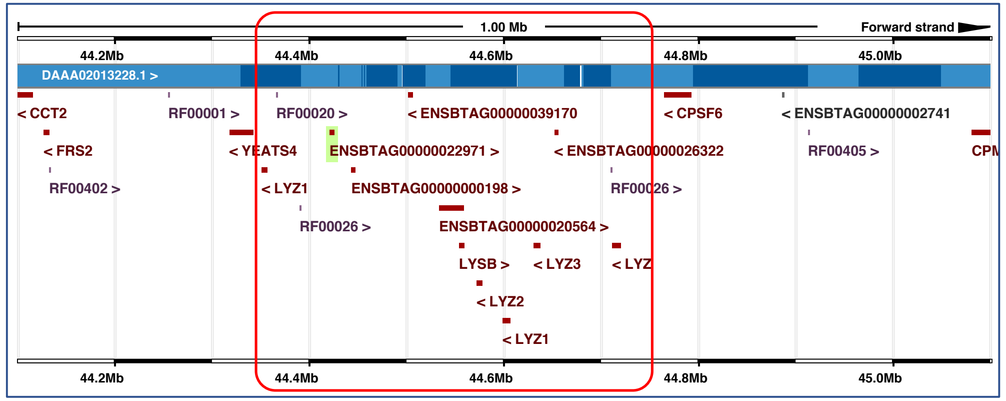

b

CNV Summarizer Dataset B: Index

Settings

Summary | CNVRs | Genes | QTLs | Samples | Run Settings

CNVRs [Filtered] Chr5:44

☆	CNVR	Chr	Start (bp)	End (bp)	Length (bp)	Type	Samples (DEL)	Samples (AMP)	Genes	QTLs
☆	Chr5:44295001-44302000	Chr5	44,295,001	44,302,000	7,000	AMP	0	47	0	1
★	Chr5:44413001-44420000	Chr5	44,413,001	44,420,000	7,000	AMP	0	44	1	1
★	Chr5:44425001-44432000	Chr5	44,425,001	44,432,000	7,000	MIXED	48	39	1	1
★	Chr5:44430001-44439000	Chr5	44,430,001	44,439,000	9,000	AMP	0	7	2	1
★	Chr5:44430001-44459000	Chr5	44,430,001	44,459,000	29,000	AMP	0	3	2	1
★	Chr5:44443001-44453000	Chr5	44,443,001	44,453,000	10,000	AMP	0	4	1	1
★	Chr5:44491001-44506000	Chr5	44,491,001	44,506,000	15,000	MIXED	229	51	1	1
★	Chr5:44520001-44526000	Chr5	44,520,001	44,526,000	6,000	MIXED	33	4	0	1
★	Chr5:44520001-44546000	Chr5	44,520,001	44,546,000	26,000	AMP	0	56	1	1
★	Chr5:44526001-44530000	Chr5	44,526,001	44,530,000	4,000	AMP	0	4	1	1
★	Chr5:44535001-44539000	Chr5	44,535,001	44,539,000	4,000	AMP	0	2	1	1
★	Chr5:44538001-44545000	Chr5	44,538,001	44,545,000	7,000	MIXED	221	1	1	1

Showing 1 to 12 of 12 entries (filtered from 10,928 total entries)

Export Genotypes | Export CSV

Overlapping Genes [Filtered] 5:44

Ensembl ID	Entrez ID	Name	Chr	Start (bp)	End (bp)	CNVR ID	CNVR Type	Overlap Type
ENSBTAG00000000198	781146		Chr5	44,443,587	44,448,198	Chr5:44430001-44439000	AMP	upstream
ENSBTAG00000000198	781146		Chr5	44,443,587	44,448,198	Chr5:44430001-44459000	AMP	upstream; utr; cds; intron; downstream
ENSBTAG00000000198	781146		Chr5	44,443,587	44,448,198	Chr5:44443001-44453000	AMP	upstream; utr; cds; intron; downstream
ENSBTAG00000020564			Chr5	44,533,886	44,559,481	Chr5:44520001-44546000	AMP	upstream; utr; cds; intron
ENSBTAG00000020564			Chr5	44,533,886	44,559,481	Chr5:44526001-44530000	AMP	upstream
ENSBTAG00000020564			Chr5	44,533,886	44,559,481	Chr5:44535001-44539000	AMP	utr; cds; intron
ENSBTAG00000020564			Chr5	44,533,886	44,559,481	Chr5:44538001-44545000	MIXED	utr; cds; intron
ENSBTAG00000022971			Chr5	44,421,169	44,426,117	Chr5:44413001-44420000	AMP	upstream
ENSBTAG00000022971			Chr5	44,421,169	44,426,117	Chr5:44425001-44432000	MIXED	utr; cds; intron; downstream
ENSBTAG00000022971			Chr5	44,421,169	44,426,117	Chr5:44430001-44439000	AMP	downstream
ENSBTAG00000022971			Chr5	44,421,169	44,426,117	Chr5:44430001-44459000	AMP	downstream
ENSBTAG00000039170			Chr5	44,502,011	44,507,108	Chr5:44491001-44506000	MIXED	utr; cds; intron; downstream

Showing 1 to 12 of 12 entries (filtered from 4,491 total entries)

Export CSV

c

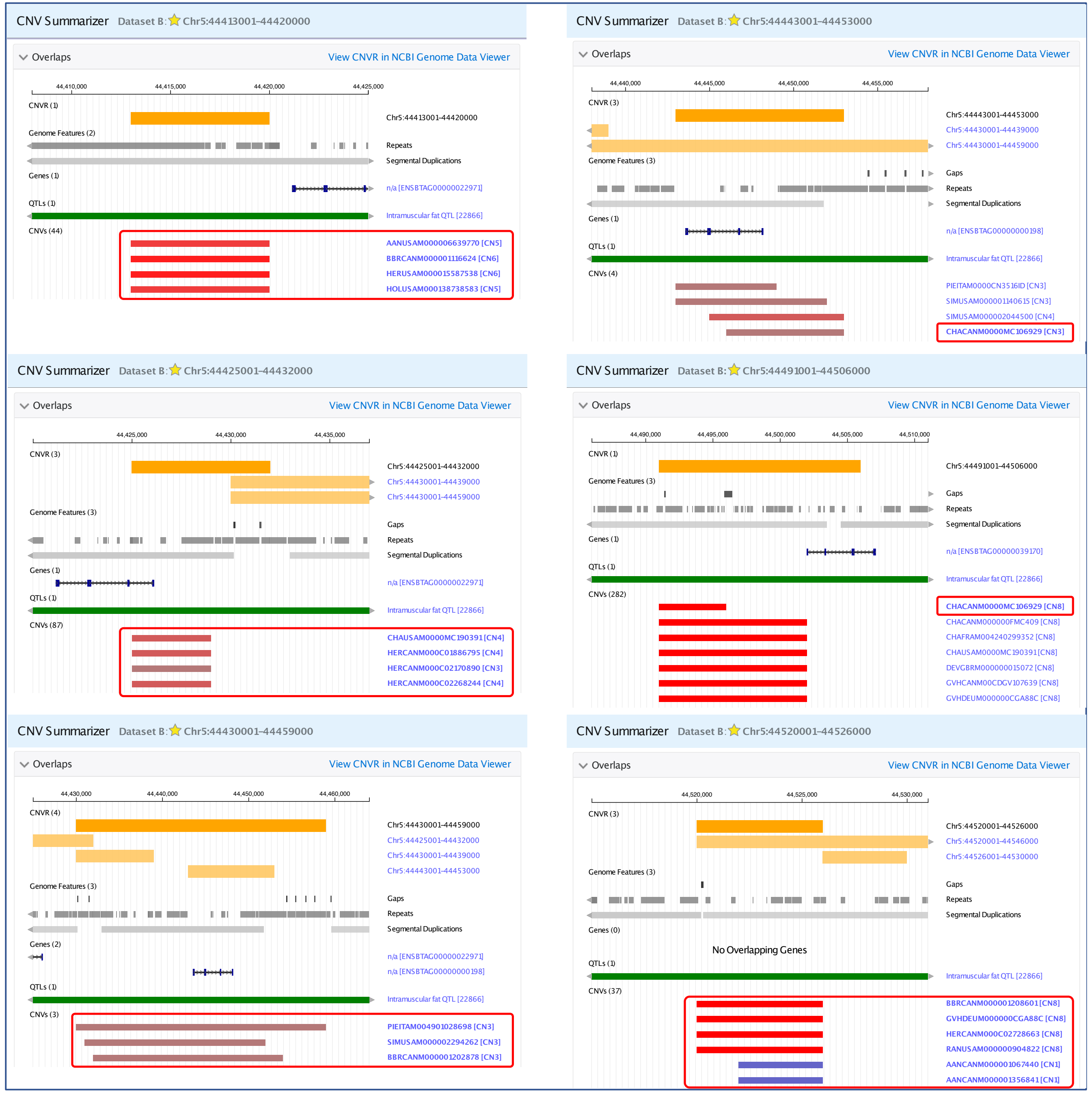

Supplement: giz073_Supplemental_Files [file giz073_supplemental_files.zip › Supplemental_Figure_S14.pdf]
