# Supplementary material for: A large interactive visual database of copy number variants discovered in taurine cattle
Source: Gigascience. 2019 Jun 26;8(6):giz073. doi: 10.1093/gigascience/giz073 (PMC6593363; doi:10.1093/gigascience/giz073)
Supplement: giz073_Supplemental_Files [file giz073_supplemental_files.zip › Supplemental_Figure_S8.pdf]

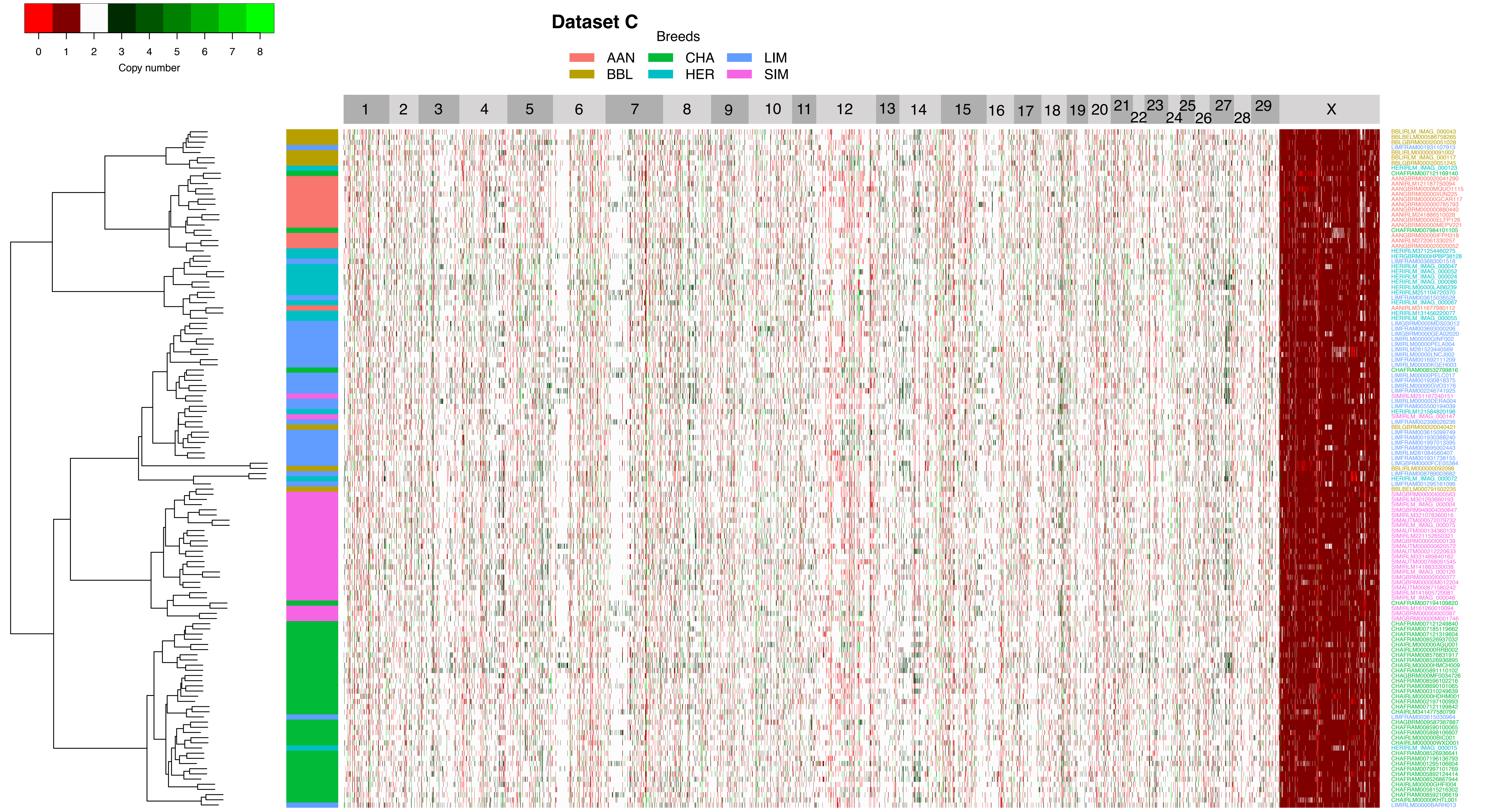

Dataset C
Breeds
AAN
BBL
CHA
HER
LIM
SIM
0 1 2 3 4 5 6 7 8
Copy number
1 2 3 4 5 6 7 8 9 10 11 12 13 14 15 16 17 18 19 20 21 22 23 24 25 26 27 28 29 X
