# Supplementary figures and images for: A large interactive visual database of copy number variants discovered in taurine cattle
Source: Gigascience. 2019 Jun 26;8(6):giz073. doi: 10.1093/gigascience/giz073 (PMC6593363; doi:10.1093/gigascience/giz073)

Sample-wise sequencing coverages

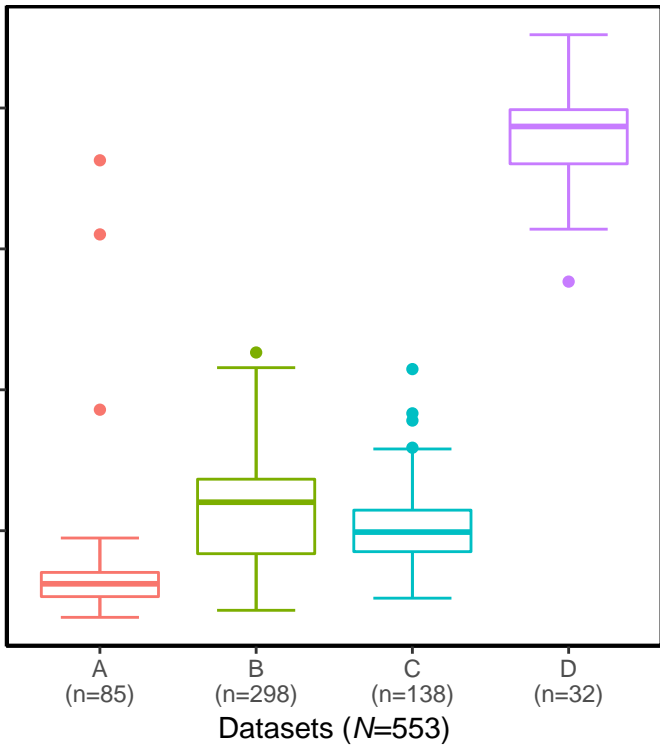

Supplement: giz073_Supplemental_Files [file giz073_supplemental_files.zip › Supplemental_Figure_S1.pdf]

a

Dataset A

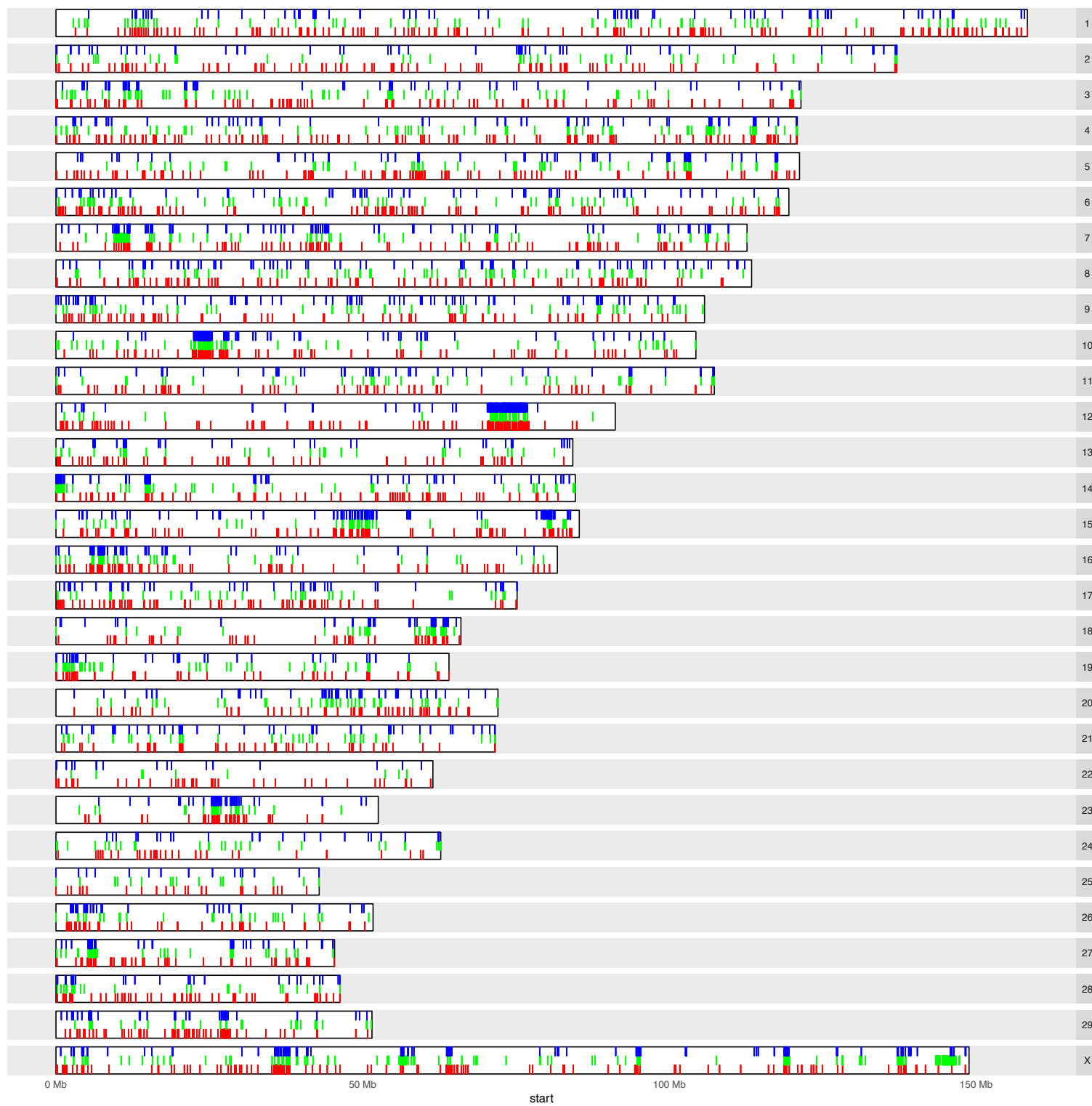

**b**

**Dataset B**

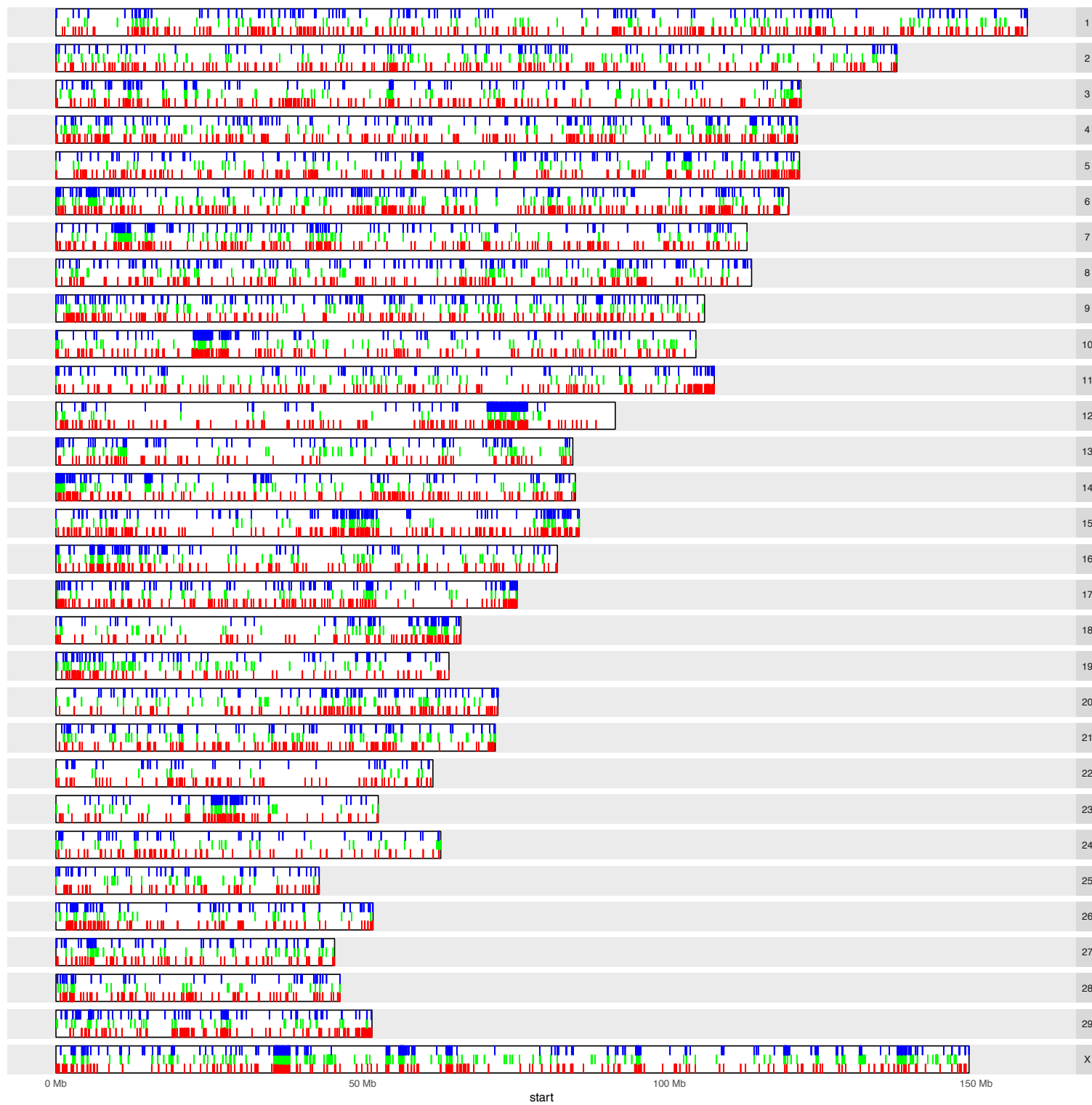

C

Dataset C

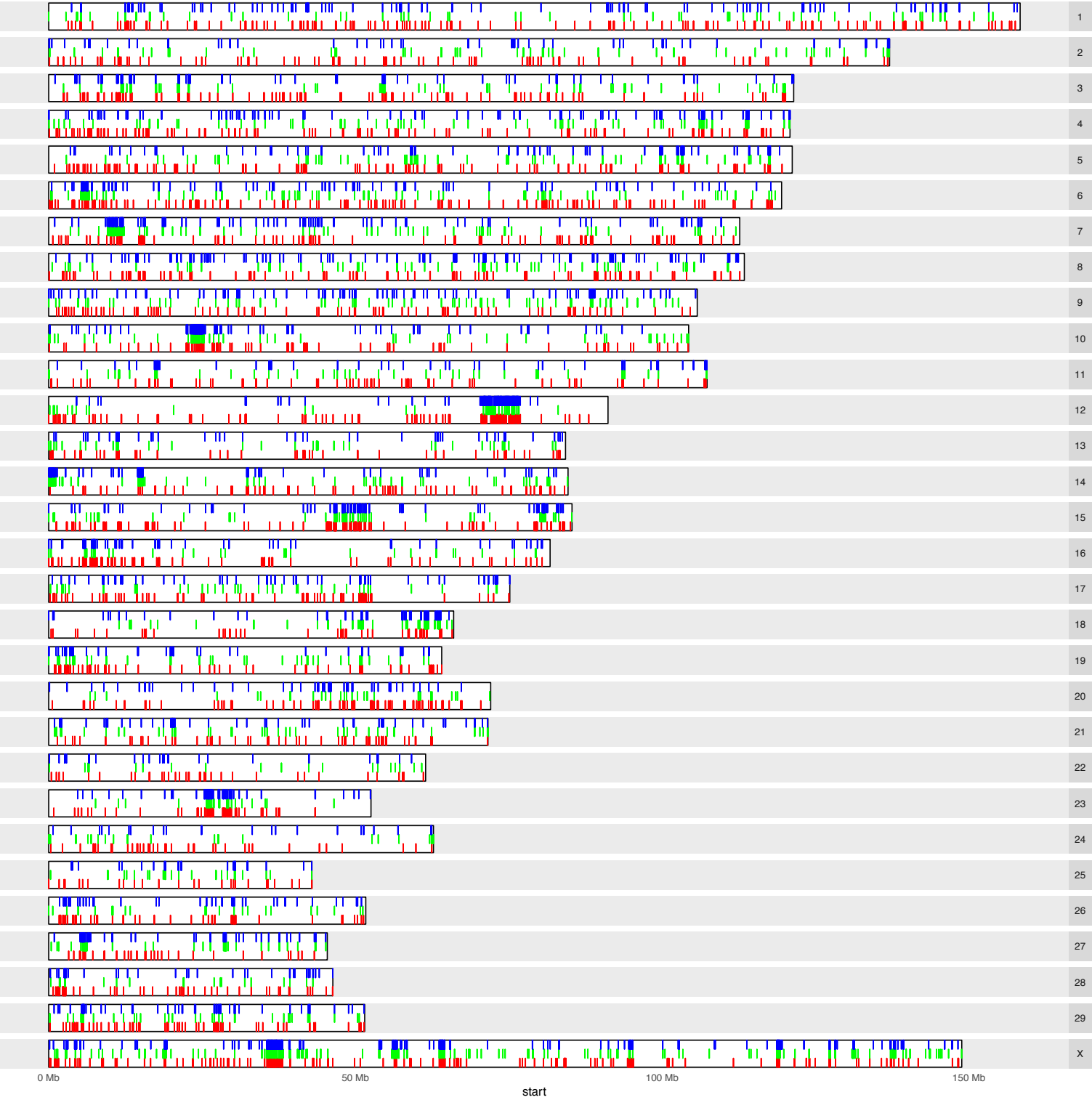

d

Dataset D

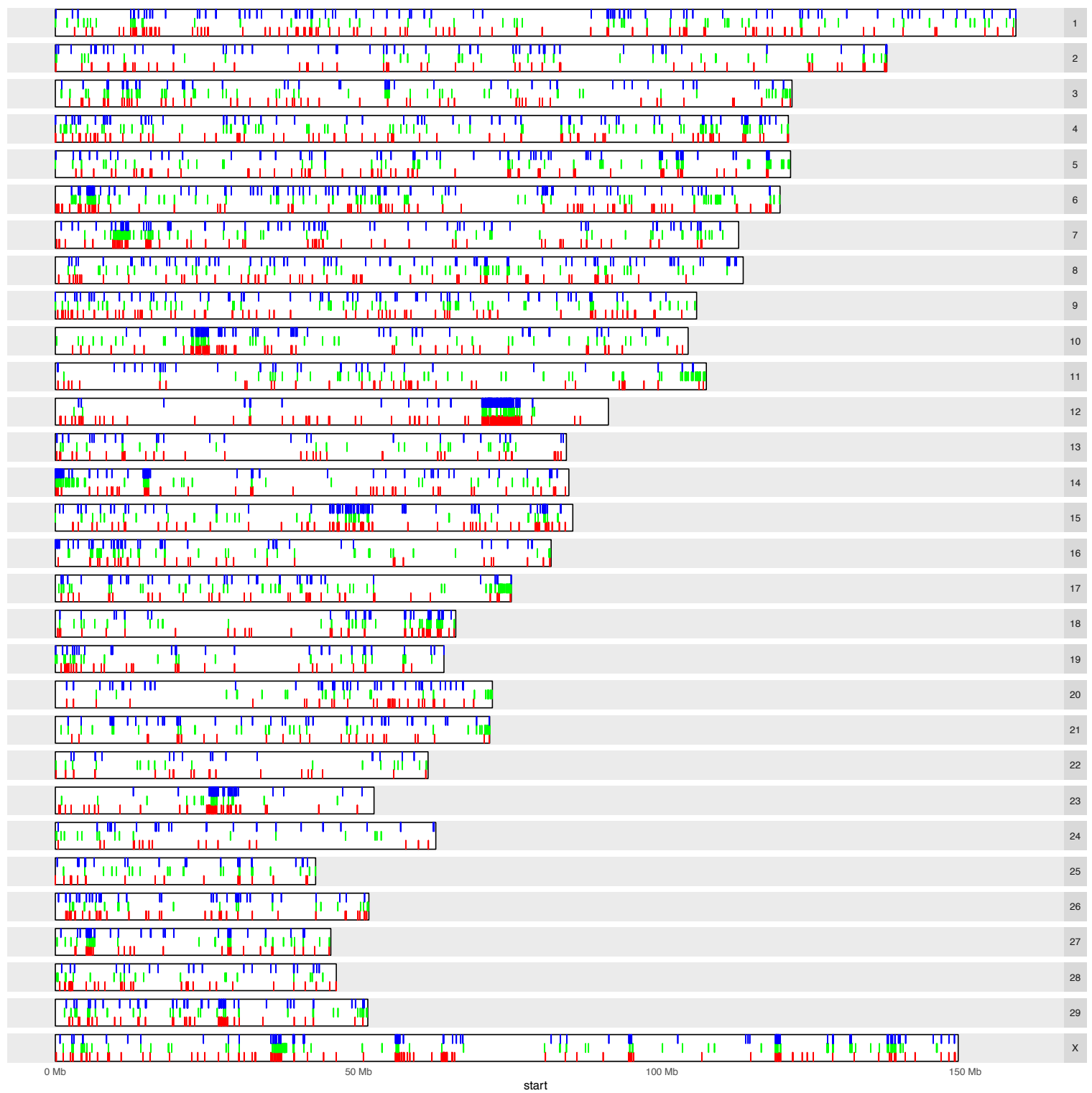

Supplement: giz073_Supplemental_Files [file giz073_supplemental_files.zip › Supplemental_Figure_S11.pdf]

a

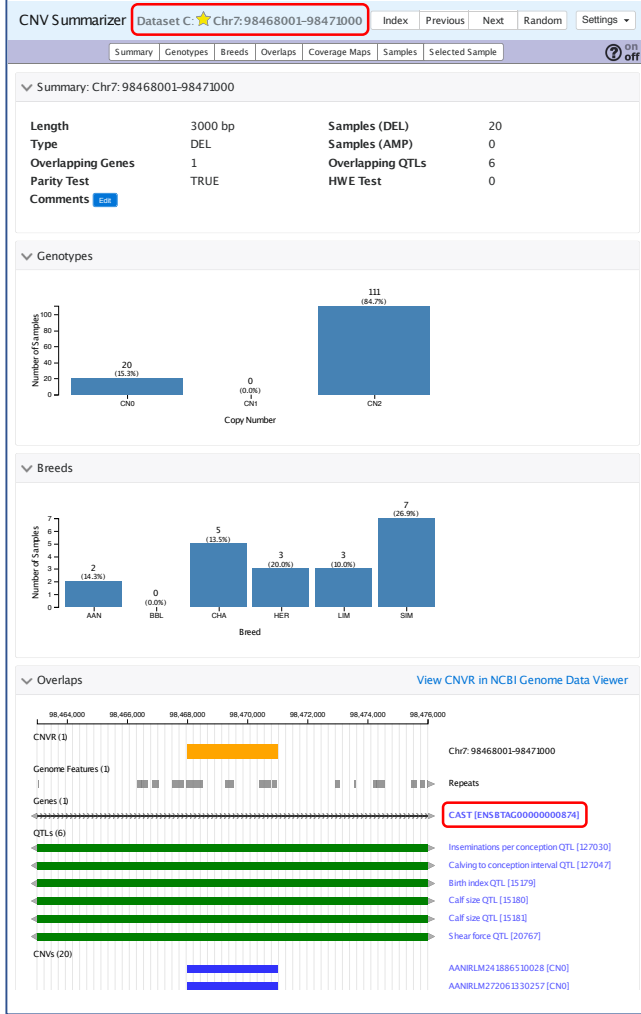

b

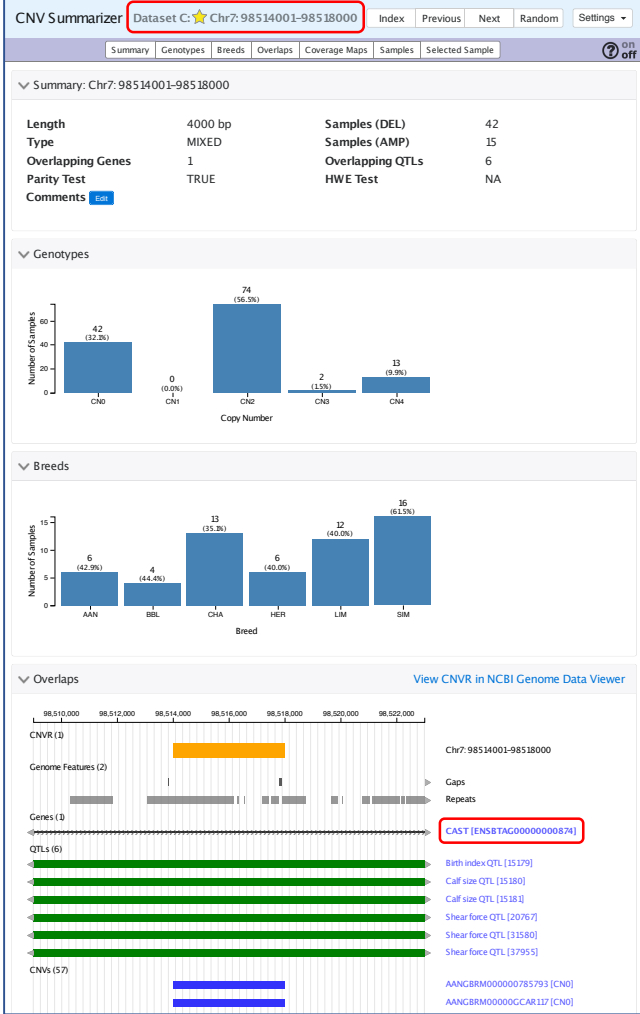

CN0

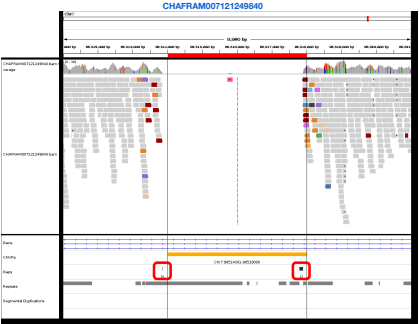

CN2

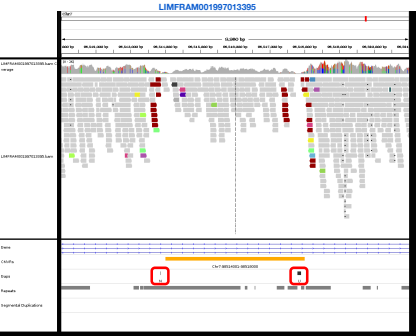

CN3

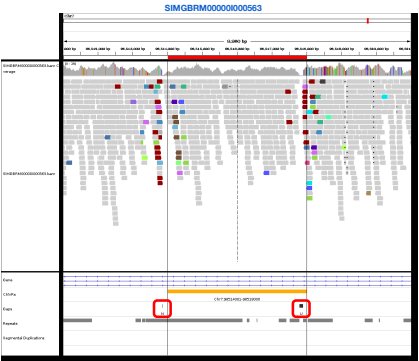

CN4

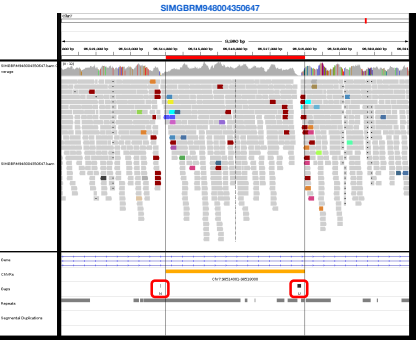

Supplement: giz073_Supplemental_Files [file giz073_supplemental_files.zip › Supplemental_Figure_S13.pdf]

a

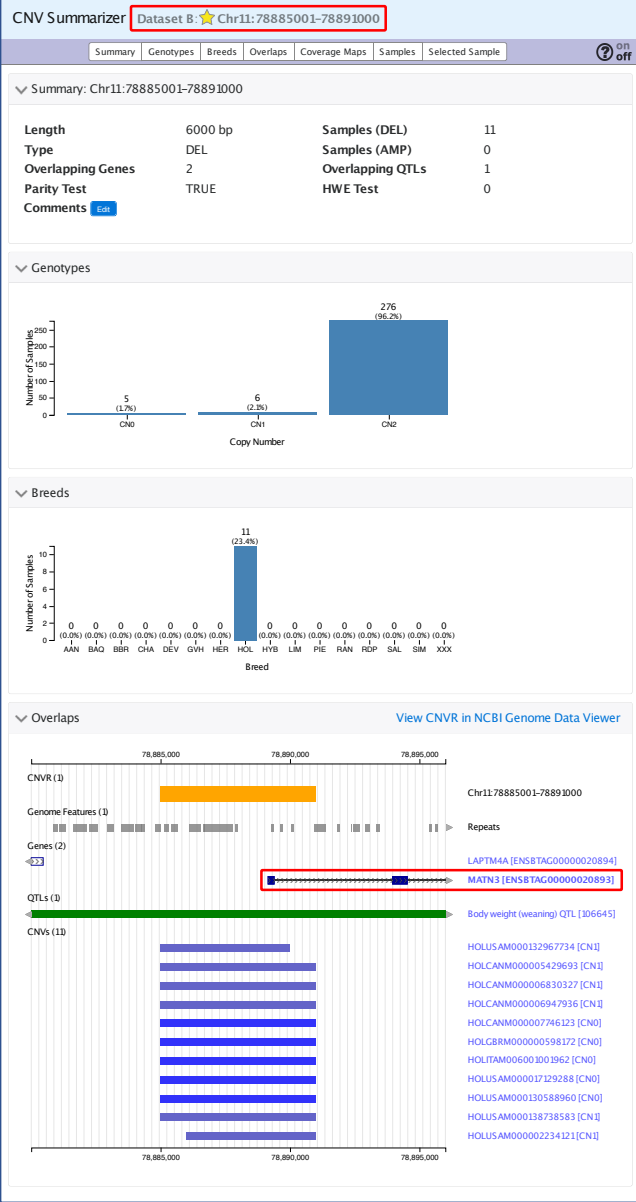

b

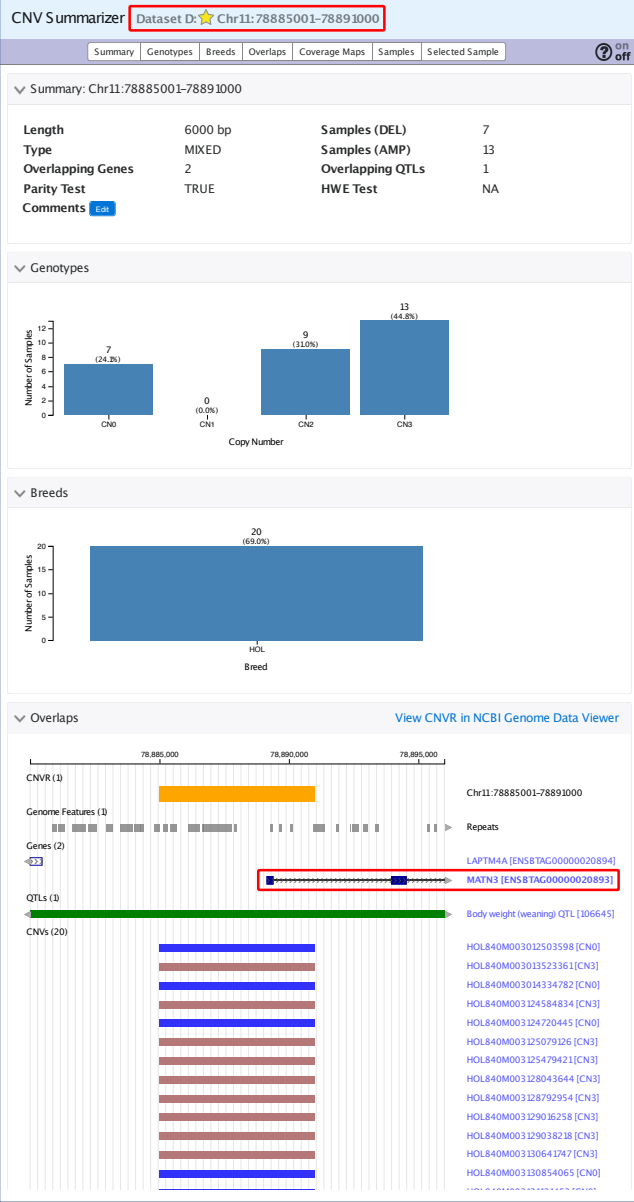

c

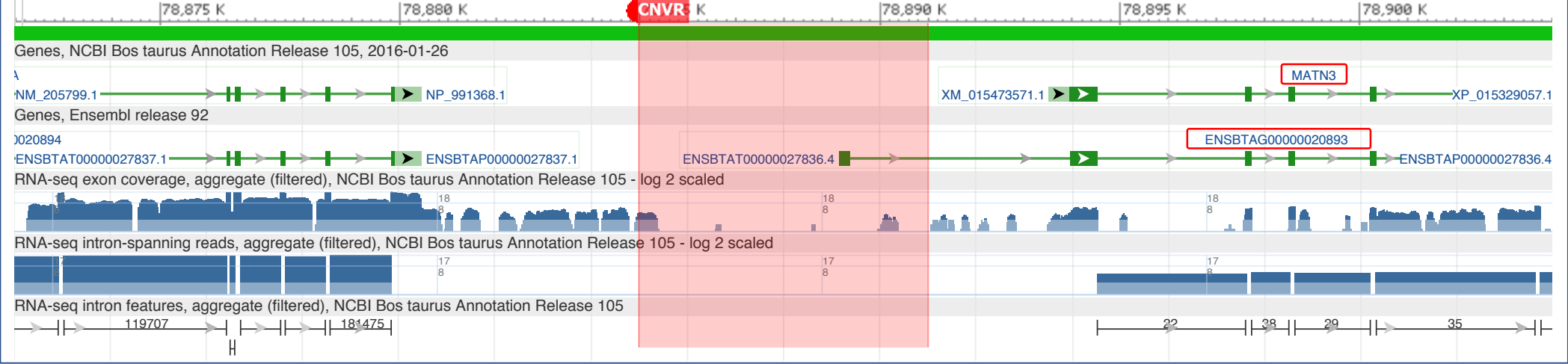

Supplement: giz073_Supplemental_Files [file giz073_supplemental_files.zip › Supplemental_Figure_S15.pdf]

a

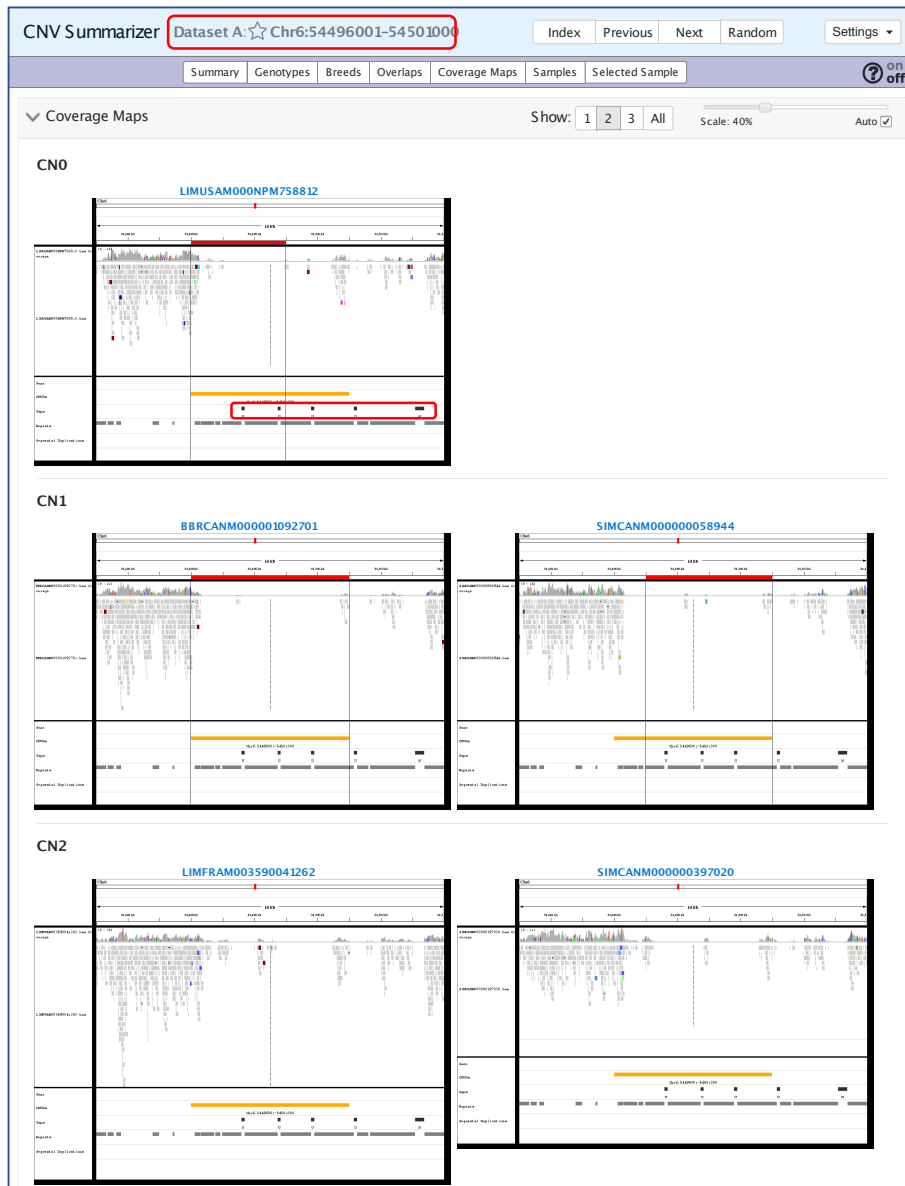

b

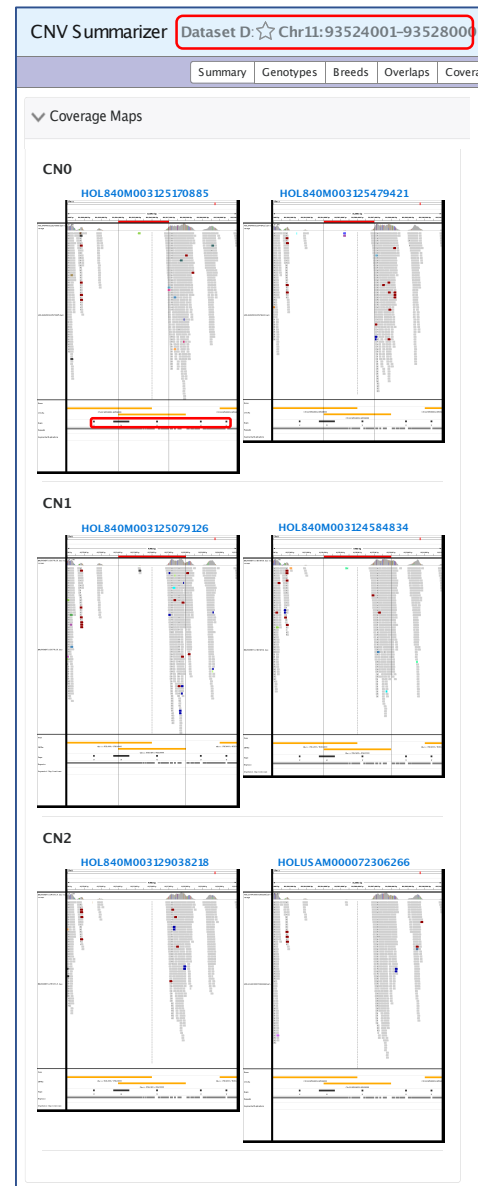

Supplement: giz073_Supplemental_Files [file giz073_supplemental_files.zip › Supplemental_Figure_S16.pdf]

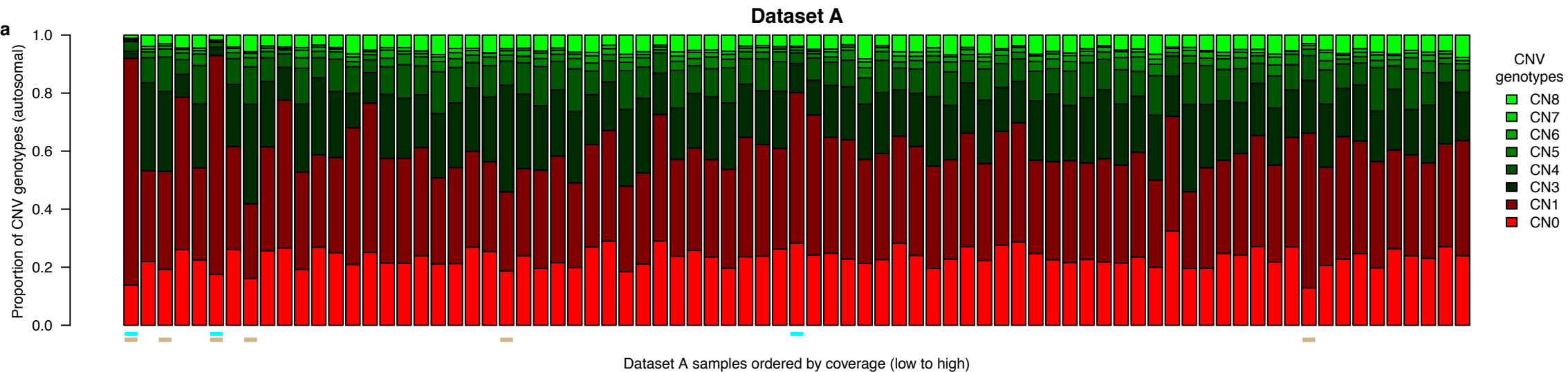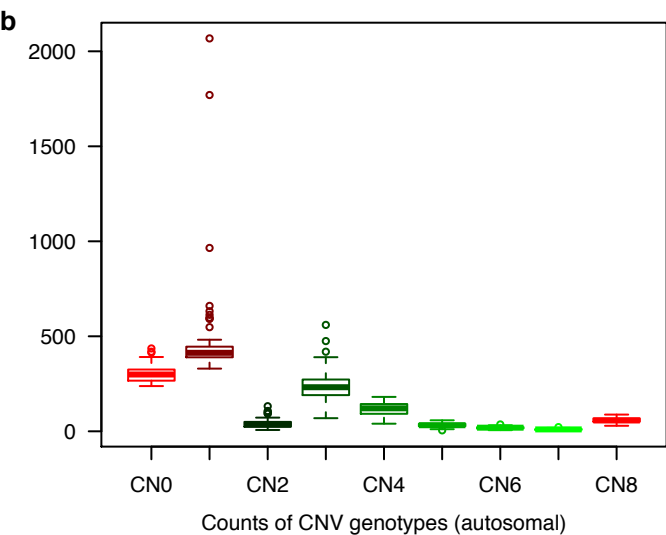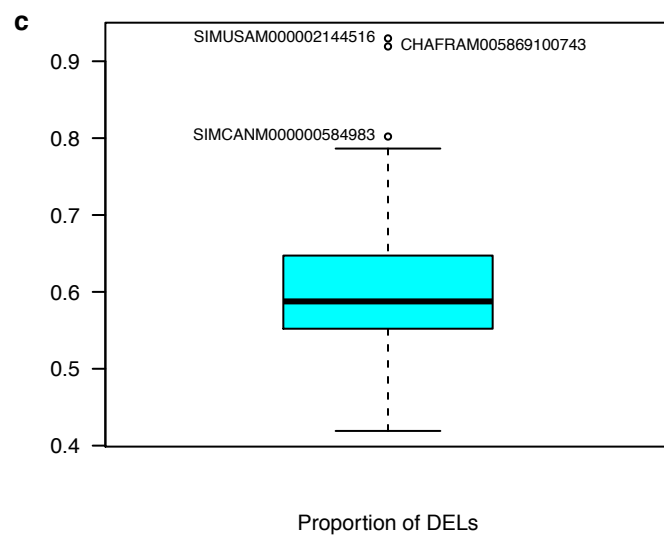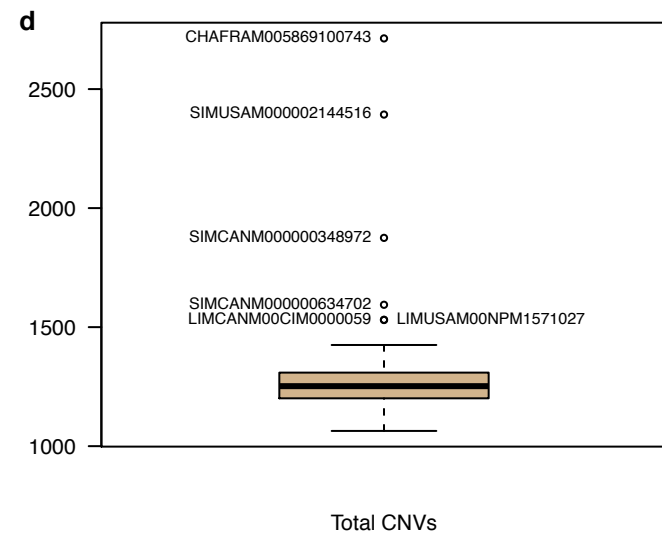

Supplement: giz073_Supplemental_Files [file giz073_supplemental_files.zip › Supplemental_Figure_S2.pdf]

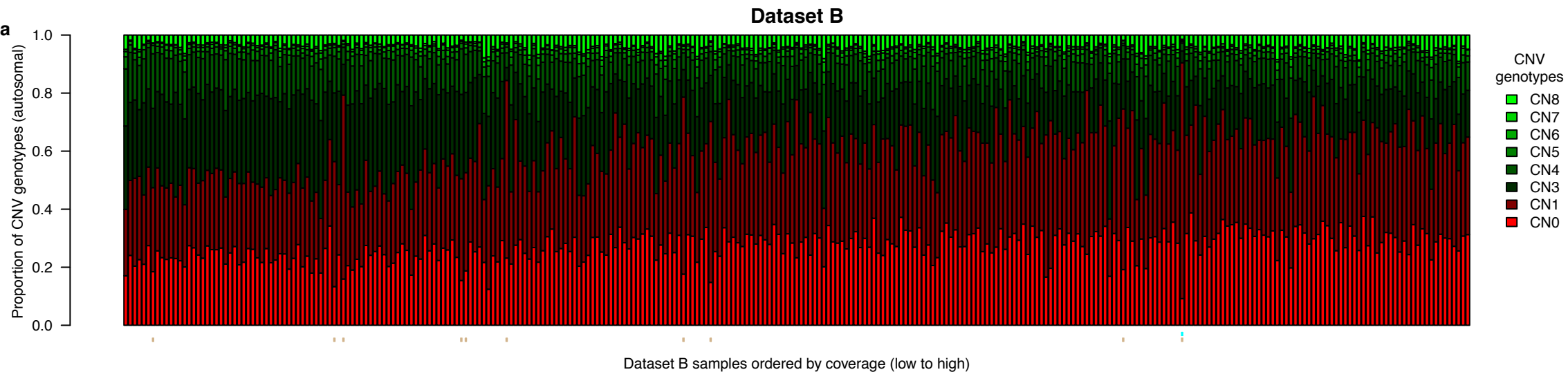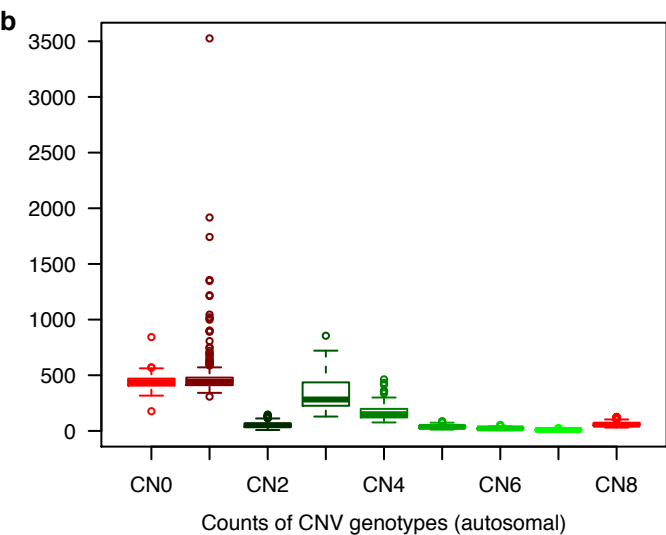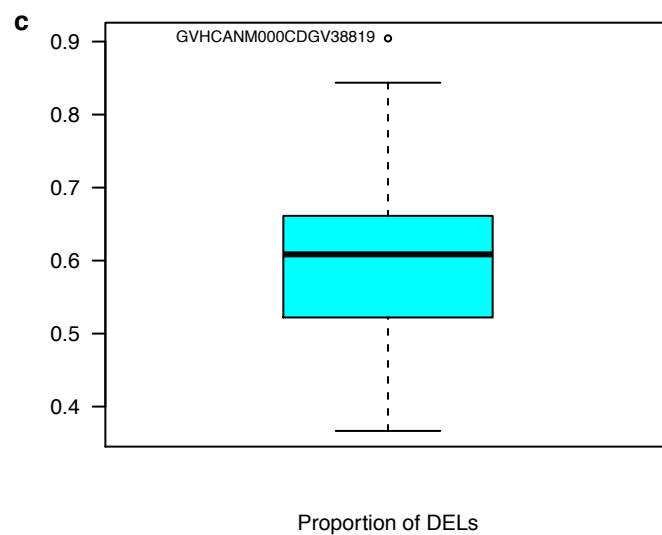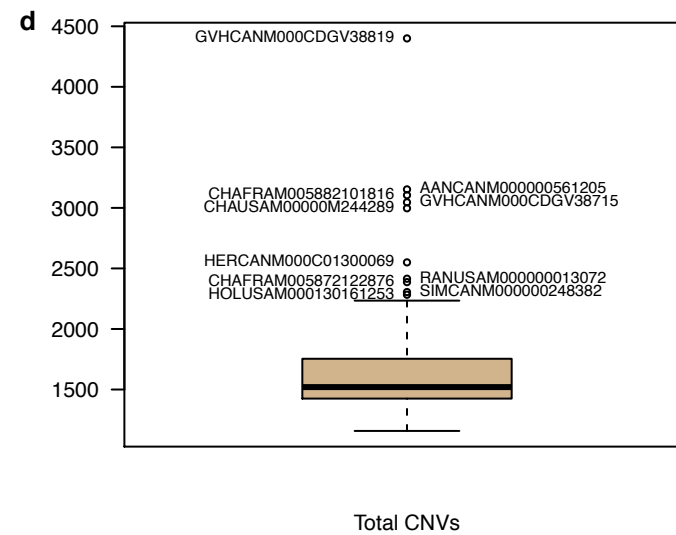

Supplement: giz073_Supplemental_Files [file giz073_supplemental_files.zip › Supplemental_Figure_S3.pdf]

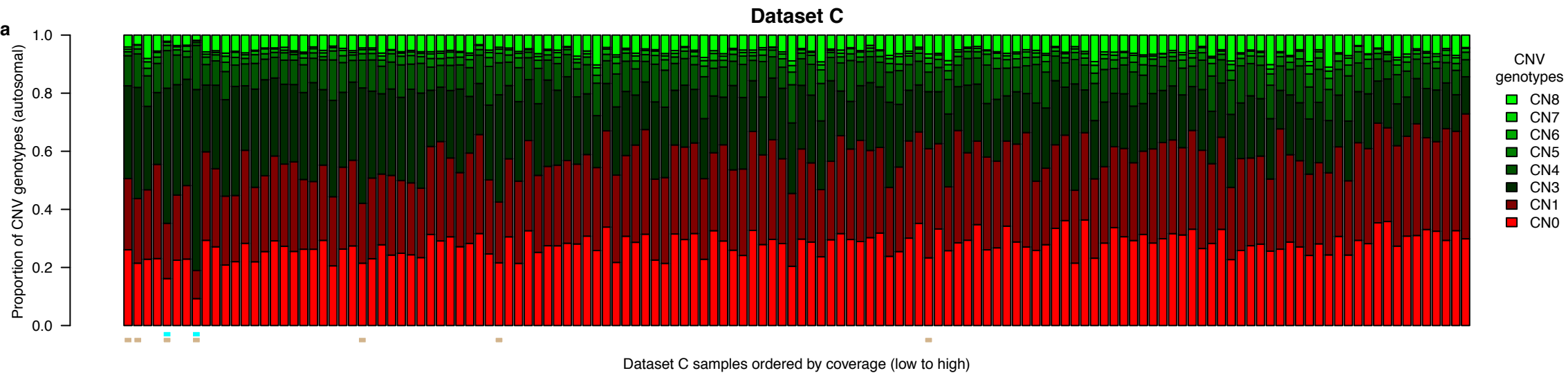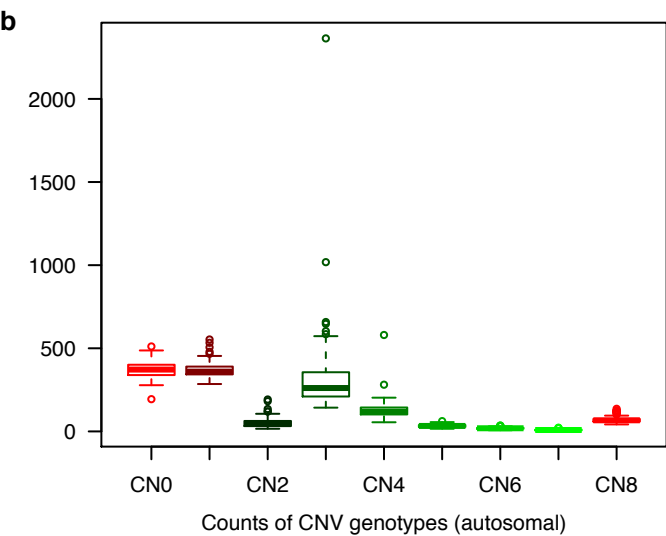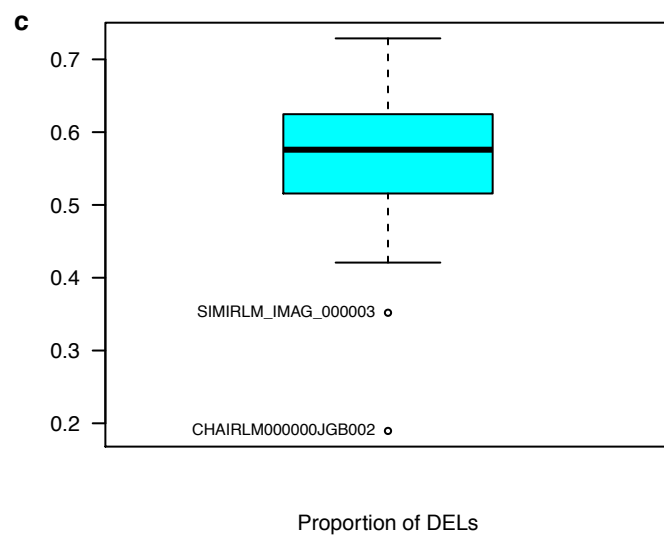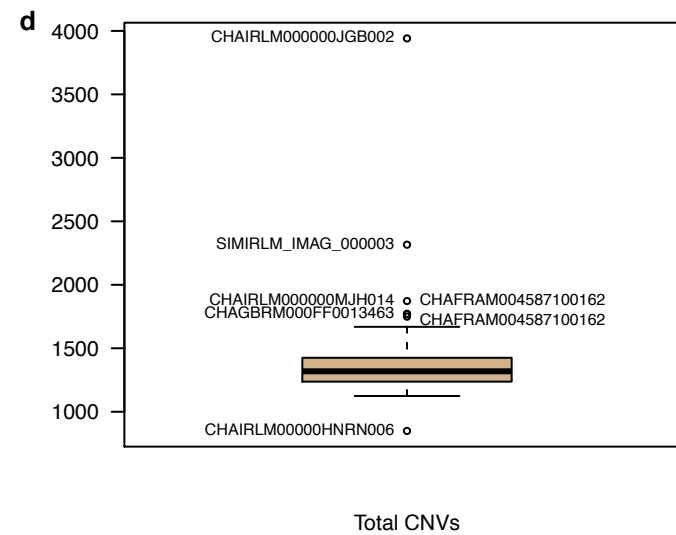

Supplement: giz073_Supplemental_Files [file giz073_supplemental_files.zip › Supplemental_Figure_S4.pdf]

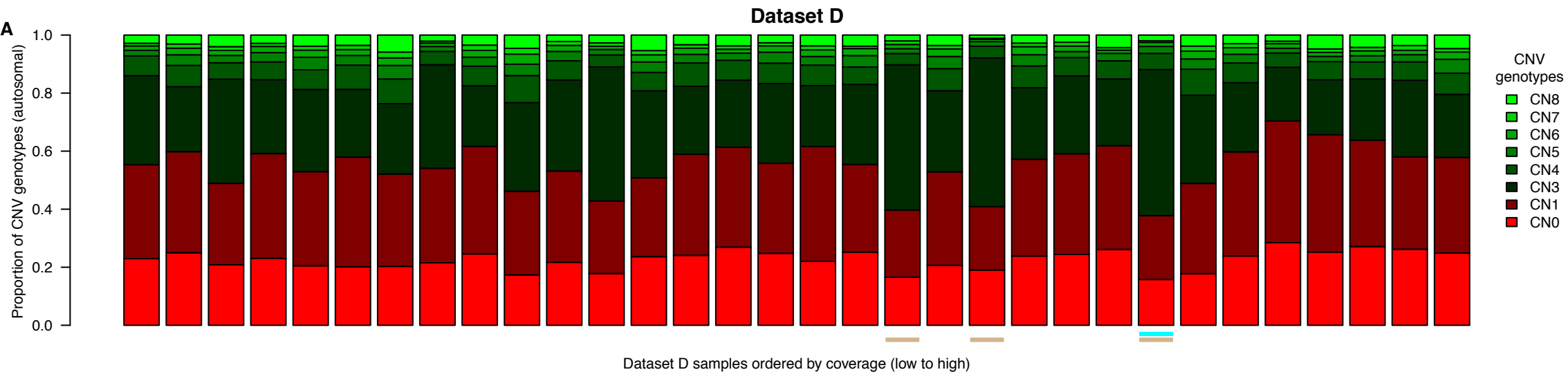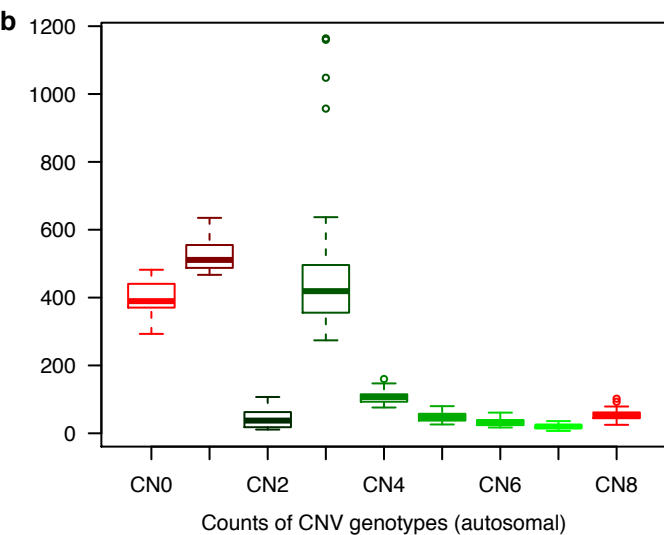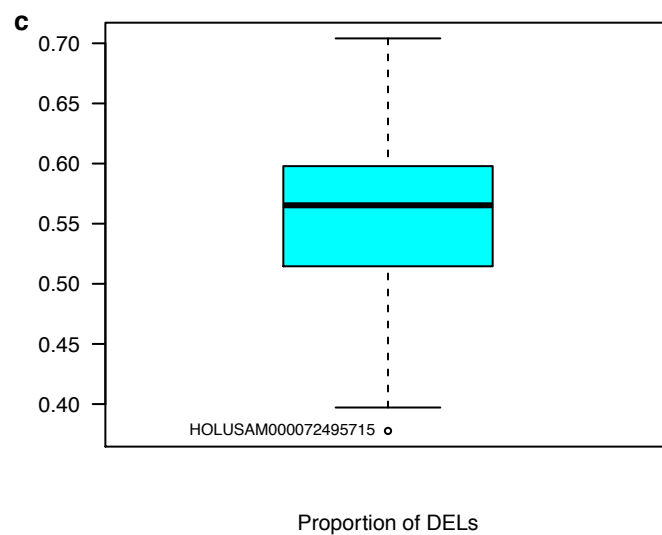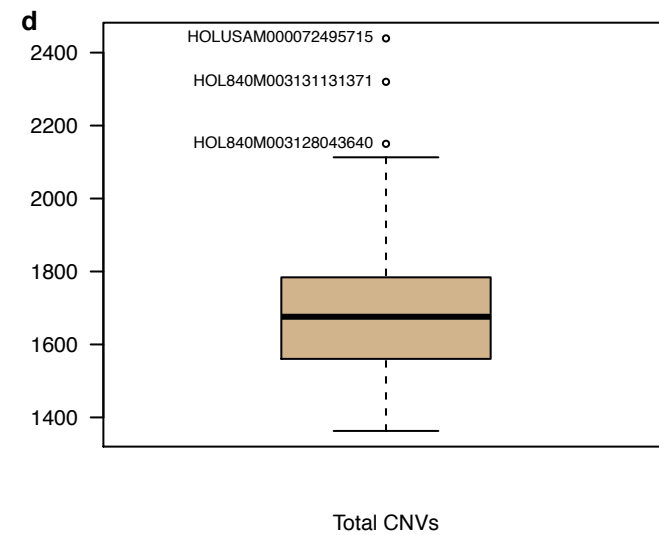

Supplement: giz073_Supplemental_Files [file giz073_supplemental_files.zip › Supplemental_Figure_S5.pdf]

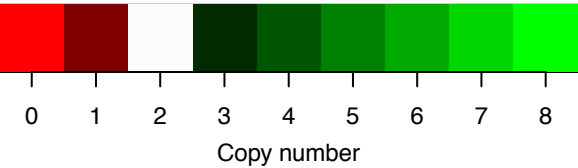

Dataset A

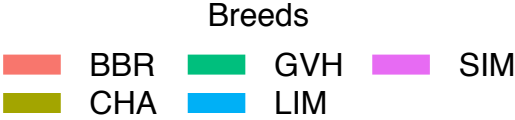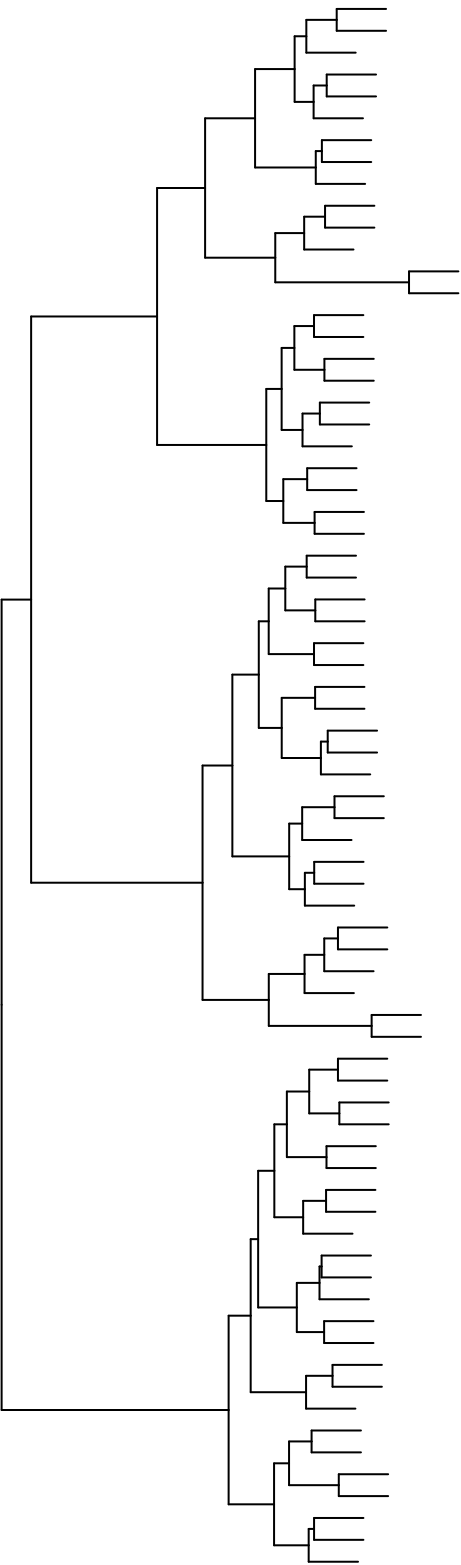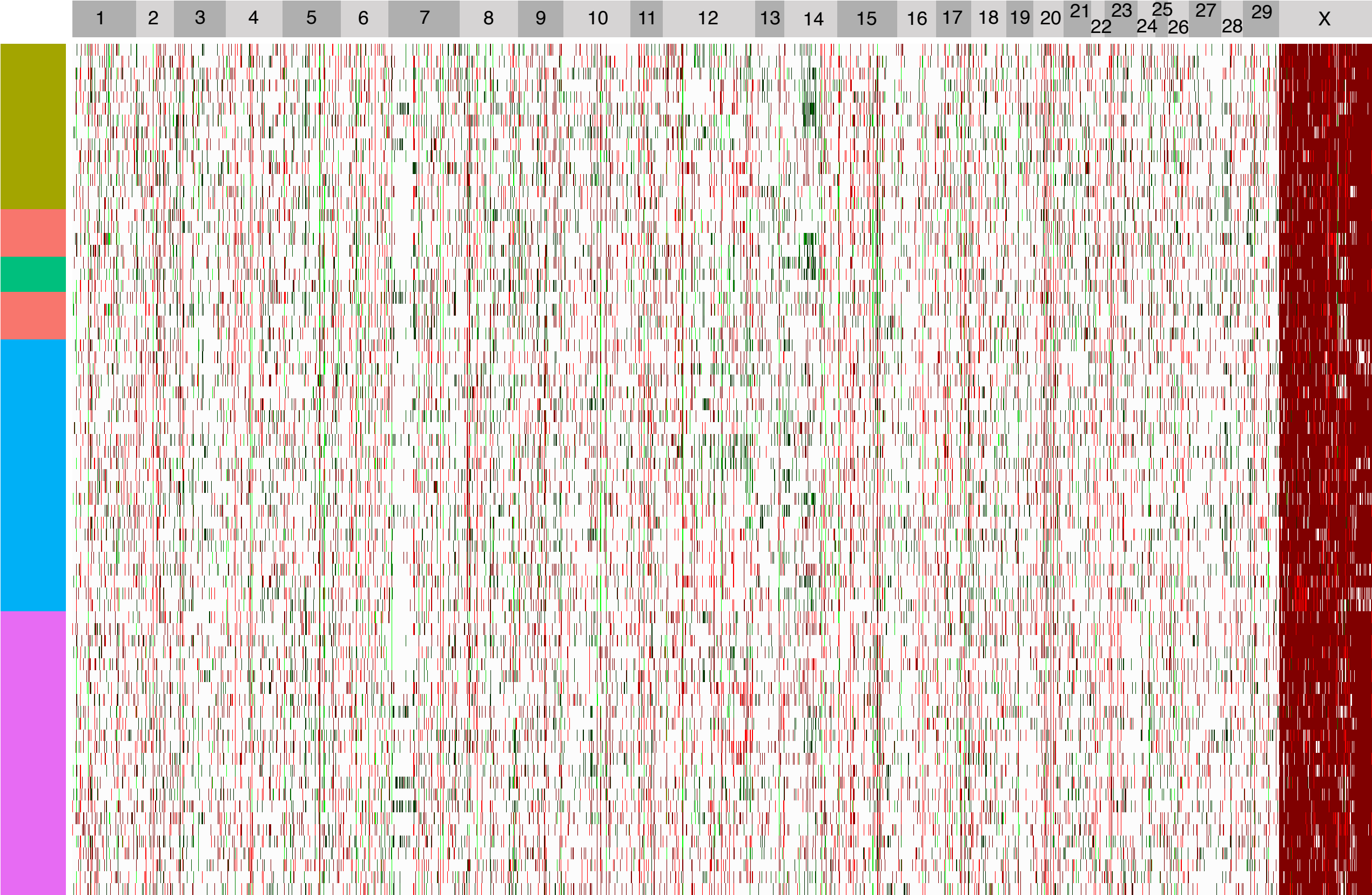

Supplement: giz073_Supplemental_Files [file giz073_supplemental_files.zip › Supplemental_Figure_S6.pdf]

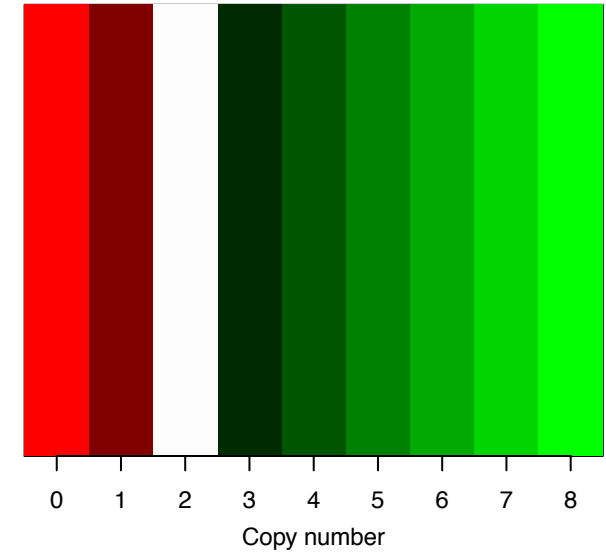

Dataset B

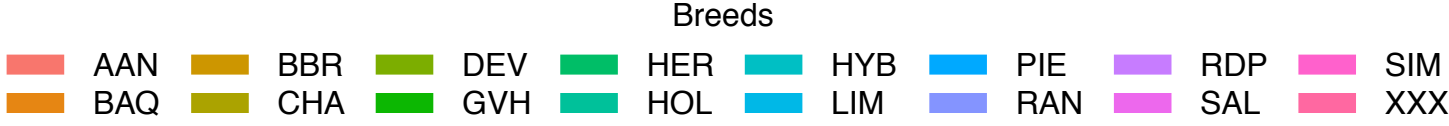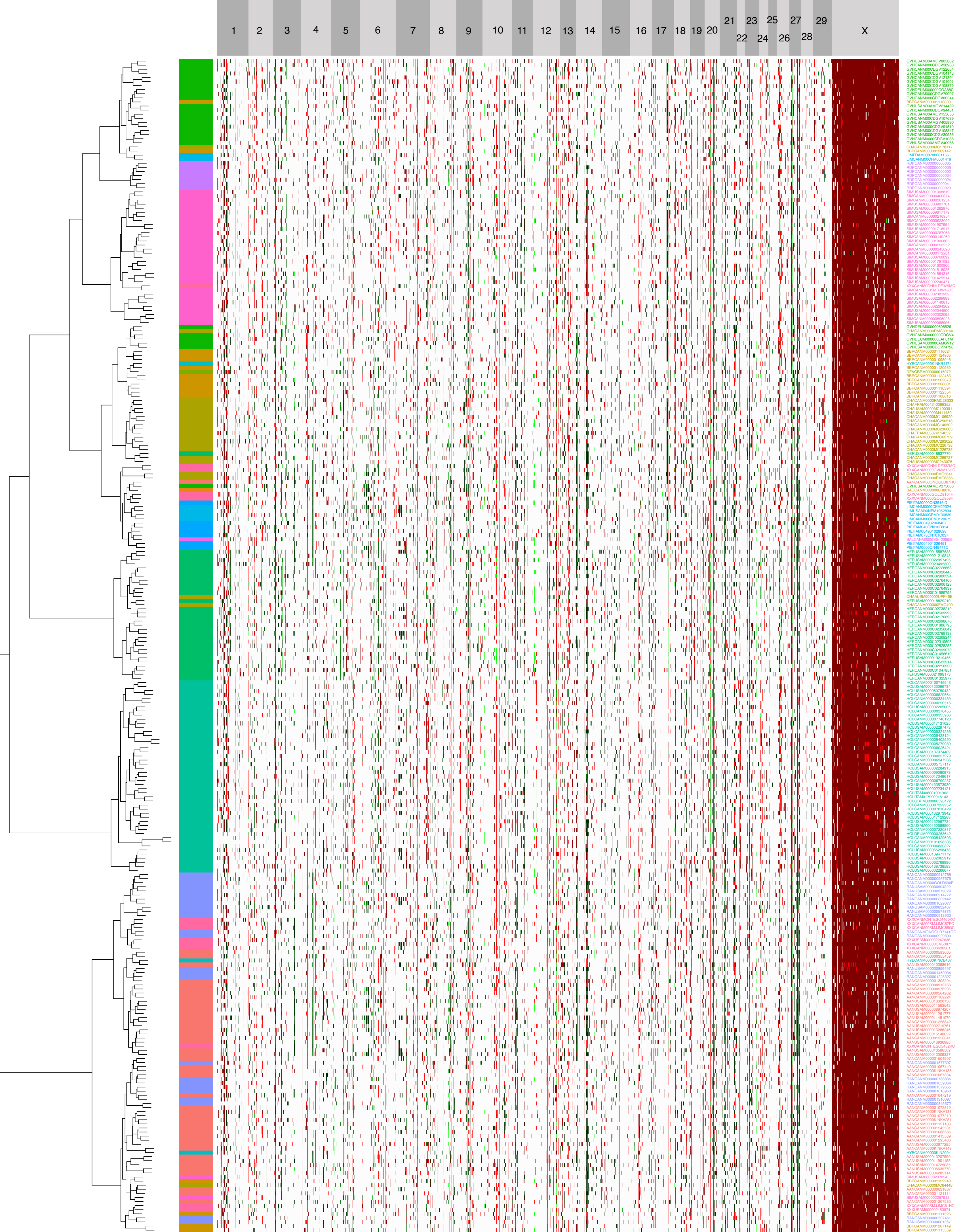

Supplement: giz073_Supplemental_Files [file giz073_supplemental_files.zip › Supplemental_Figure_S7.pdf]

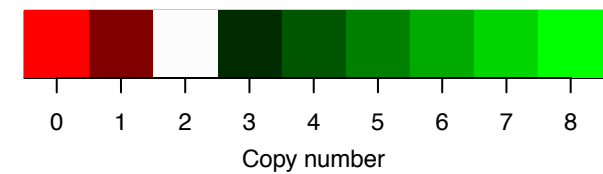

Dataset D

Breeds

HOL

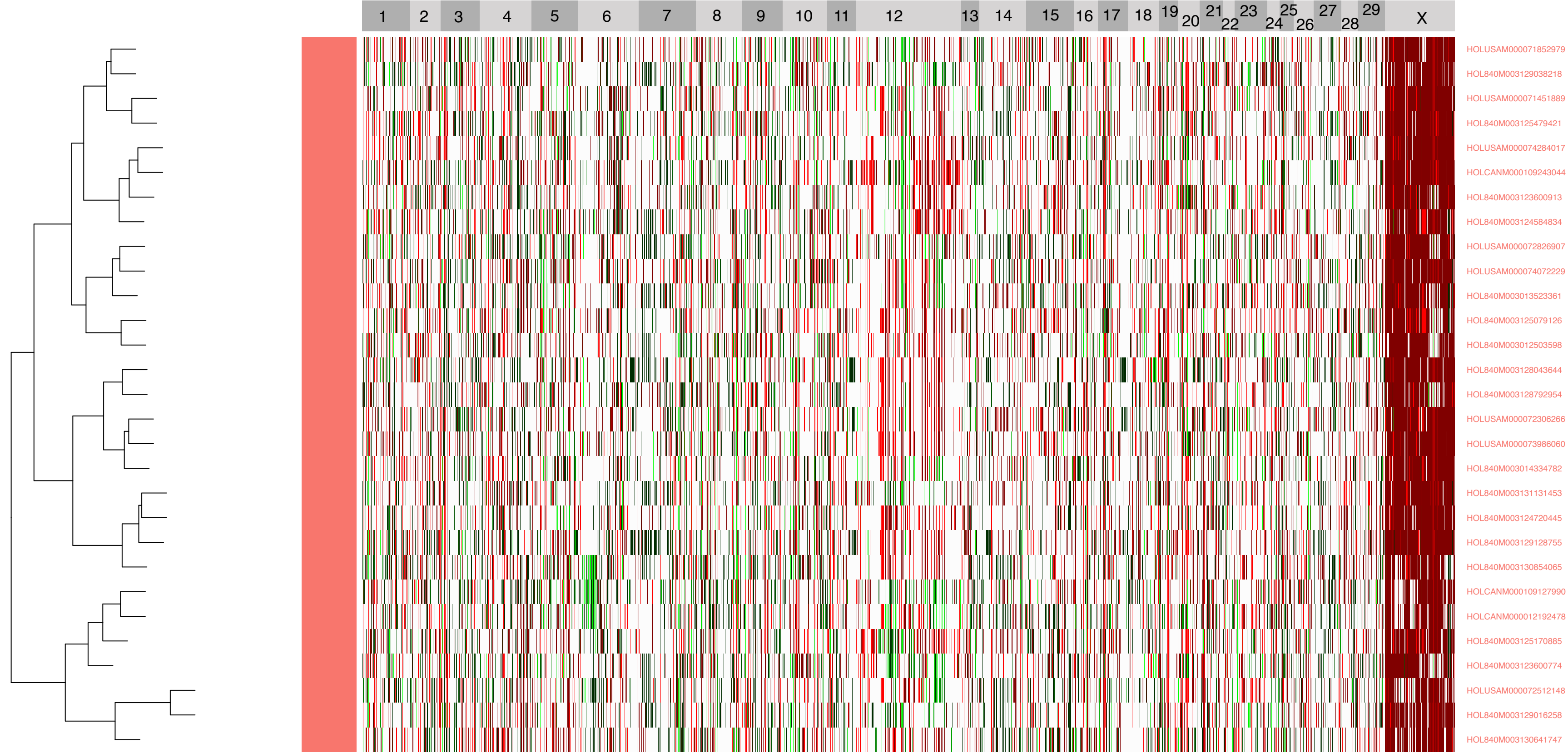

Supplement: giz073_Supplemental_Files [file giz073_supplemental_files.zip › Supplemental_Figure_S9.pdf]
