# Supplementary material for: A large interactive visual database of copy number variants discovered in taurine cattle
Source: Gigascience. 2019 Jun 26;8(6):giz073. doi: 10.1093/gigascience/giz073 (PMC6593363; doi:10.1093/gigascience/giz073)
Supplement: giz073_Response_to_Reviewer_Comments_Original_Submission [file giz073_response_to_reviewer_comments_original_submission.pdf]

Author's Response To Reviewer Comments

Close

February 27, 2019

Dear Editorial Board of GigaScience,

We are pleased to submit a revised version of our manuscript where we addressed all comments from the reviewers. We found the reviews very useful and thank the reviewers for their time and effort.

Please note that the order of authors generated earlier through Gigascience's submission portal was different from how it is in the manuscript. Also, one of our co-authors has requested to add an initial for his middle name. We were able to make these changes ourselves through Gigascience's submission portal.

We thank you for receiving our revised manuscript and look forward to a positive review.

Sincerely,

Paul Stothard
(Corresponding author on behalf of all authors)

==
Response to reviewers' comments:
==

==
Reviewer #1:
==

This manuscript describes the discovery and genotyping of copy number variations (CNVs) in multiple datasets using a single CNV algorithm together with the development of a platform that integrates multiple tools to allow identified CNVs to be easily assessed visually in multiple ways. The development of the platform will be of great use to the field of CNVs as streamlines the otherwise laborious task of characterizing individual CNVs. The manuscript describes selected CNVs as examples of this platform. The manuscript is well written and represents a significant advance to the field. There are some points in the manuscript I feel could be improved upon:

We thank you for your thorough review and valuable comments that helped us improve our manuscript. We have addressed your comments as listed below and highlighted in yellow the corresponding changes in the revised manuscript. Some other changes that we made, mostly grammatical, are highlighted green.

==

Lines 45-48 The authors say they are presenting the "the largest CNV set". However, no genome wide validation of the CNVs appears to have been undertaken (although the database described is set up in a manner to allow researchers to validate). Given that CNV discovery is plagued by false positives having the "largest set of CNVs " does not necessarily mean the best set - producing the largest set of putative CNVs is easy, but determining which are real, is hard. These sentences should be rewritten, particularly given that later in the manuscript the authors do acknowledge gaps affect the detection of CNVs.

Thanks for pointing this out. We do understand that the size of the predicted CNVs does not mean much unless validated. What we wanted to emphasize was the breath of this set of CNVs, which was generated using over 500 bulls from 17 different breeds. We have rephrased the sentence as follows (Page 2; Line 45):
"We present a large comprehensive collection of taurine cattle CNVs in a novel interactive visual database that displays CNV boundaries, read depths and genome features for individual CNVRs, thus providing users with a powerful means to explore and scrutinize CNVRs of interest more thoroughly."

==

Line 75-81 The authors indicate that there has been no genome wide validation of CNVs called by various CNV algorithms, however, Couldrey et al 2017 - this paper did successfully undertake genome wide assessment (Detection and assessment of copy number variation using PacBio long-read and Illumina sequencing in New Zealand dairy cattle). The introduction should be amended to include this information, the manner in which the authors wrote this sentence implies that they have undertaken genome wide validation which they have not done.

Thanks for bringing this omission to our notice. We have referenced that study and added the following to the introduction (Page 4; Lines 79-91):
"Couldrey et al. [31] illustrated the use of long-read sequence information combined with a CNV transmission-based approach to confirm a subset of CNVs that segregate in the New Zealand dairy cattle population. Briefly, the putative CNVs discovered from long-read sequence information in a prominent Holstein-Friesian bull used in New Zealand were first compared with those discovered from short-read sequences in the same bull. Next, a population of 556 cattle representing the wider New Zealand dairy cattle population were short-read sequenced and genotyped at those putative CNV regions, followed by a genome-wide assessment of transmission level of copy number based on pedigree. Visual assessment of highly transmissible CNV regions provided additional evidence to support the presence of CNV across the sequenced animals. Currently, the high cost of long-read sequencing limits adoption of this approach to large numbers of animals representing different breeds, and other studies that provide supportive evidence on a genome-wide scale to help assess the quality of CNVs predicted from short-read sequencing or SNP array data are extremely limited."

==

Please include further discussion about the accuracy of breakpoints for CNVs and CNVRs, are these consistent between samples, if not, how does this affect analysis between samples and accuracy of assigned copy number genotype? Please also comment further on how the same genomic location was defined, if a CNVR in one animal was a subset of a larger one in another (or there was an overlap between animals) were these counted as unique or separate CNVRs

The breakpoints for CNVs at a CNVR are not always consistent across all samples in which they are identified and this does not mean the breakpoints identified are inaccurate as can be verified from the visualisation provided for read alignments at the CNVR. A consequence of having multiple adjacent CNVs at a CNVR that vary in their breakpoints by one or more windows of 1 Kb (the window size parameter we chose for cn.MOPS algorithm) is that a simple merging of those CNVs using the default CNVR construction method in cn.MOPS would result in some CNVRs that are abnormally large. Also, we would then lose information on the CNV diversity across samples at that CNVR and the genotype assigned to that CNVR may not be representative of the underlying CNVs. Our solution to avoid those issues was to use a 50% pairwise reciprocal overlap criterion to construct CNVRs following which genotypes were assigned based on a set of rules as described in the Methods section. Therefore, at the same genomic location, if a CNV in one animal was a subset of a larger one in another, the decision to merge them into a single CNVR or to keep them as separate CNVRs was dependent on the mutual overlap between those CNVs. More details on these points can be found in sections "Constructing CNVRs from CNVs" and "Assigning genotypes to CNVRs" of the Methods section. Also, to emphasize the above points, we have added the following to the Discussion section:
Page 19; Lines 421-427:
"We did not use the built-in function within cn.MOPS to construct CNVRs and assign CNVR genotypes as we found that this approach can produce very large CNVRs which obscure the underlying breakpoint diversity across samples and that have genotype assignments that are not always consistent with the majority genotype observed among the constituent CNVs. We therefore employed a 50% pairwise reciprocal overlap criterion to construct CNVRs, as has been used in other studies [26,29] and then

assigned genotypes based on a set of rules as described in the Methods section."
Page 19; Lines 435-440
"Read coverage and alignments within and adjacent to a CNVR can aid in the determination of the breakpoints of constituent CNVs in individual samples, as the resolution of the breakpoints reported by the cn.MOPS algorithm is limited to the choice of window size used for CNV detection. The visualisation of genome features like assembly gaps and repeats can highlight potential non-CNV related coverage and alignment anomalies, and thus can further be used in the assessment of predicted CNVs and their breakpoints."

===

Line 214 please indicate if the CNVR upstream of KIT is the causative as determined by J. Taylors lab or another CNVR in the same area (also reported by the Taylor lab).

We had looked into that CNVR too and we have added the following lines to the Results section "Identification and genotyping of the well-characterised KIT locus CNV in our datasets" to address this (Page 13: Lines 257-263):
"Another CVNR, approximately 15 Kb in size (Chr6:71810000-71825000) and located within intron 1 of the KIT gene, has been reported to be associated with the piebald coat color [36] In our analysis, the only CNVR that overlaps with this region and that shows amplification in the majority of HER and some SIM animals is an 11 Kb CNVR at Chr6:71808000-71819000, identified only in dataset B. This CNVR was detected in 25 of the 31 HER (24 as CN3 and 1 as CN8) and 7 of the 34 SIM (all as CN3) individuals in dataset B. Thus based on our results, the CNVR at Chr6:71747001-71752000 (upstream of the KIT gene) is more clearly associated with the piebald coat color."

===

Given that historically deletions have been significantly easier to detect from short read sequence using read depth please add some discussion on the low frequency of deletion detection relative to duplication

Following quality control (QC), the frequency of DEL type CNVs was still higher than AMP type in all our datasets with means of the proportions of DELs ranging between 0.57 (SD 0.06) for dataset C and 0.60 (SD 0.07) for dataset B. Samples with outlier proportions of DEL type CNVs, generally seen in low coverage samples (see Figures S2-S5), were removed during QC as indicated in the Results section, "Distributions of CNV genotypes were more consistent across datasets that were analysed individually": "Additional quality control (QC) steps were applied to identify problematic samples, defined as those that showed marked deviations (i.e., 1.5 times the interquartile range away from the first and third quartiles) in the proportion of DELs or total CNVs discovered within each dataset."

===

===
Reviewer #2
===

This paper describes CNV(R) detection in sequenced cattle of four different datasets. It highlights some important issues that may arise during CNV detection in data from different sequencing platforms, but also in CNV detection in general. The authors have provided a valuable database for future research that can serve as a resource to confirm CNVs and visualize them.

We thank you for your thorough review and valuable comments that helped us improve our manuscript. We have addressed all your comments as listed below and highlighted in yellow the corresponding changes in the revised manuscript. Some other changes mostly grammatical that we made are highlighted green.

===

I have not been able to find the actual database on GigaDB. This makes it hard to value the usefulness.

The database was uploaded on GigaDB but the editor had informed us about the delay in making it available to reviewers. It should be accessible now.

===

My main remark is that there are a number of recommendations done, for which the reason and tools to do so are lacking (e.g., HWE) or unrealistic (e.g., confirm genome wide CNV visually), see the line by line comments. There are many methods to predict CNV, the authors seem very confident about their approach, but other than the platform effect, there are limited results shown to back that. Visualizing seems to be the key. However, this is very unpractical when screening the whole genome of hundreds of individuals. On the other hand, when interested in a specific CNV visualisation to confirm the CNV is needed, and then this database would be useful for confirmation.

HWE is provided as an additional annotation, however, we do not recommend its use as a criterion to filter CNVRs. We edited Lines 296-299 (Page 14) to clarify this:
"HWE results are provided as an additional characteristic / annotation of CNVRs but we caution against filtering CNVRs based on HWE as the test is limited to diallelic autosomal CNVRs and deviations from HWE could reflect inaccurate genotypes for an otherwise true CNVR of interest."

The strength of our database, as you indicate and we emphasize, is to provide the user the ability to assess individual CNVRs of interest (for example, CNVRs in the vicinity of a candidate gene or GWAS peak) among CNVRs identified on a genome wide scale. We do not claim to provide genome wide validation of CNVRs.

Our rationale for the choice of the CNV prediction software used in our research is discussed in our response to one of your specific comments on this that follow.

===

L122 You refer to 'batch effect' here however to me it seems a sequencing platform effect. Can different batches sequenced on the same platform with similar coverage be combined? Was that the case here? With different batches I mean a group of animals sequenced in January and another group in June for instance (same platform, same intended coverage).

We call this a “batch effect” and not a “sequencing platform effect” as we found that samples sequenced using the same platform (Illumina) but differing in read sequence length despite similar coverage also clustered separately based on CNV genotypes. The time of sequencing may have had an effect too through differences in sequencing chemistries within the same sequencing platform technology.

===

L122 Please indicate that the CNV discovery referred to here was done in the complete dataset

We have rephrased the sentence to indicate this (Page 6; Line 135):
"Proceeding with CNV discovery and genotype characterization using those read counts from all datasets together (after excluding the four PCA outliers) revealed considerable differences in the distribution of CNV genotypes per dataset (Figure 1b)."

===

L129 Change figure 1b into figure 1a

There is no reference to figures at this line. The references to Figure 1 in the previous lines have been verified to be correct.

===

L152-153 When you remove samples should you not rerun CNV detection?

Reruns of the CNV detection algorithm after removing samples based on outlier status might still detect new samples as outliers. So we perform the sample filtering only once to capture the most significant outliers and remove them. Further, of the CNVRs generated from the filtered set of CNVs, only those that were identified in at least 2 samples were kept. The quality of the resulting CNVRs is evident based on the clear clustering of samples by breed (Figures S6-S9) based on CNVR genotypes.

==

L243-244 Which version number of Chrome/Firefox is that? Or will this be true in the future as well?

Either browser updated to a recent version, not necessarily “latest” should work fine. Future versions of those browsers should be ok too as they would be built to be backward compatible and we have used standard features of html and JavaScript to provide functionality. In L270 (Page 13), the word "latest" has been replaced with "recent":
"(for best results, use a recent version of Google Chrome or Mozilla Firefox)."

==

L254 Does this mean that the CNVs detected with UMD3.1 can now be plotted in the new genome build ARS-UCD 1.2?

It is possible to map the CNVRs we identified on UMD3.1 onto ARS-UCD1.2 using liftOver coordinates now available for this assembly. However, we have not currently implemented that feature in our database. We have edited L281 (Page 13) to clarify that the links we generated to view CNVRs on the NCBI genome data viewer are also based on UMD3.1.
“Additionally, a link to the NCBI Genome Data Viewer (www.ncbi.nlm.nih.gov/genome/gdv/) [38] plots the CNVR region in the context of the latest annotations and genomics data available in NCBI for the UMD3.1.1 bovine reference genome assembly.”

==

L261 Were HWE and parity test part of the QC mentioned in table 2?

No, they were not used in the QC but are provided as an additional resource to users.

==

L264-266 This sentence confused me. If 63-88% of the CNVR that passed HWE had a p-value >=10exp-5 what was then your threshold to qualify HWE? It is also not given in M&M.

As mentioned in section "Hardy–Weinberg equilibrium (HWE) test on CNVR genotypes" of the Methods section, the CNVRs that qualified for HWE testing were diallelic autosomal CNVRs with either a combination of CN0, CN1 and CN2 genotypes (considered as minor allele homozygous, heterozygous, and reference homozygous) or CN2, CN3 and CN4 genotypes (considered as reference homozygous, heterozygous, and minor allele homozygous). Multi-allelic CNVR genotypes were not tested for HWE here because of the inability to determine what combination of alleles were responsible for a particular genotype. We edited Line 291 (Page 14) to include "diallelic autosomal" CNVRs:
"Of the diallelic autosomal CNVRs that qualified for the HWE test per dataset (53-57% of the total for the 4 datasets; see Methods), the majority (63-88%) had genotype proportions that were in HWE (Chi-squared test p-value ≥ 10-5)."

==

L268-270 Why do you recommend this and how should one verify this? It seems that you know something you forgot to inform us about. Why not simply discard them, as we do with SNPs?

We have replaced that line with the following clarification (Lines 296-299; Page 14): "HWE results are provided as an additional characteristic / annotation of CNVRs but we caution against filtering CNVRs based on HWE as the test is limited to diallelic autosomal CNVRs and deviations from HWE could reflect inaccurate genotypes for an otherwise true CNVR of interest."

==

L287-288 I didn't see this discussion in previous section. Yes HWE was mentioned but there is no proper discussion on the reasons of deviation from HWE and how your database can verify them. So please add. See also previous comment.

We have edited Lines 316-319 (Page 15) to address this:
"As discussed in the previous section, deviations from HWE should not be used as a criterion to filter CNVRs; instead visualisation of the read coverage and other supporting information at the CNVR available through the CNVR database will help validate the predicted CNVs."

==

L313 Remove additional '.' at end of sentence

Thanks, this is now corrected.

==

L386-387 Can you back this superiority with a reference? Are methods that combine for instance read depth and paired end reads not better?

There is no consensus on what method is best as different methods have different strengths and weaknesses. Methods combining read depth and paired-end information will have some strengths that cn.MOPS does not and vice-versa. The one obvious strength in a multi-sample test like cn.MOPS is their superior ability to control for false discovery rate. While the issue of FDR can be moderated in approaches that use paired-end read information, this limits their discovery power, especially in regions of low coverage. These points are all discussed in the cn.MOPS paper along with benchmarks on the performance of cn.MOPS against 5 other methods that showed cn.MOPS performed significantly better than those methods based on precision-recall curves; we therefore reference the cn.MOPS paper here in Lines 417-421 (Page 18) along with another paper by Keel et al. (2017) where it was shown through simulation studies that read-depth based methods (which included cn.MOPS) performed better than paired end and split read based methods in the majority of datasets composed of samples at carrying levels of sequencing coverage:
"Software to predict CNVs has also evolved and methods that rely on multi-sample read-depth analyses, like cn.MOPS, have become popular due to their superior ability to control for false discovery rate [32]. Furthermore, a recent study on simulated data has reported read depth based approaches to perform relatively better than those based on paired end and split read analyses when analyzing datasets comprised of samples sequenced at varying levels of coverage [26]."

==

L410-411 Interesting comment about imputation. Like to get your thoughts on this subject. Based on what can you perform imputation for CNV? Pedigree? SNPs? The SNP-CNV LD seems very limited for imputation, but perhaps there is more information in sequence data.

We have looked into this further and choose to remove this statement in light of additional reading which revealed the challenges involved in imputation of CNVs from SNP data and the non-availability of pedigree information for our datasets.

==

L427-429 Do you mean visualizing each CNVR one by one? Noble recommendation, but that is a pain in the ass job, for instance for 26000 CNVR detected in your study. Have you visualized them all? I assume only the ones you report in detail about. Isn't that the reason why we apply methods like cn.MOPS, to automate the job. I know that CNV calling methods have their issues, but on a genome wide scale checking each detected CNV manually seems a bit too much to do, especially as sample sizes are getting bigger and bigger. I agree that CNVR of interest that will be explored further because of association or something, they should be manually checked, but genome wide is a bit much I would say, unless you can automate it.

The strength of our database, as you indicate and we emphasize, is to provide the user the ability to verify individual CNVRs of interest (for example near to a gene or genome region highlighted through some other research activities) among CNVRs identified on a genome wide scale. We do not claim to provide genome wide validation of CNVRs and we have added/edited the sentence (Page 21; Lines 468-470) to avoid implying that:
"We recommend that visualisation of read coverage at predicted CNVRs be a standard protocol in studies

reporting specific CNVRs of interest (for example near to a gene or genome region highlighted through some other research activities) among CNVRs identified on a genome-wide scale."

==

L457 Can you add why you chose cn.MOPS? There are so many methods, and as you indicated the method is relevant.

Please refer to our earlier response to your comment regarding, “Can you back this superiority with a reference”. Our datasets consisted of large number of samples sequenced at moderate coverage, we could leverage the strength of cn.MOPS algorithm to control FDR through its multi-sample approach.

==

Figure 4 more distinctive colours for the bars would be appreciated

The colours used here are kept consistent with the colours we used in several other plots to represent genotypes: gradients of red for DELs and green for AMPs. Hence we keep to the same colour scheme here. However, to assist with interpretation, we have added a genotype label at the base of each bar in the revised figure 4.

==

Figure 5 Hard to see what is in the panels, and the database itself I could not find on GigaDB. Also in Supplementary material figure S12-16.

The editor had informed us of the delay in making the database available for the first review but that it is now available to reviewers.
Figure 5 is meant to be an overall illustration of the key features of the functionality of the CNVR database, and therefore we prefer to keep those panels in one figure. We understand the panels would not be readable in its original size when printed on paper, and hence we had annotated them with labels and described each panel in the accompanying legend. However we did note resolution issues with some figures and have recreated several at higher resolution including figures S12-16, so that users can view them at a larger size / zoom in to see much more detail.

==

Close
