# Supplementary material for: A large interactive visual database of copy number variants discovered in taurine cattle
Source: Gigascience. 2019 Jun 26;8(6):giz073. doi: 10.1093/gigascience/giz073 (PMC6593363; doi:10.1093/gigascience/giz073)
Supplement: giz073_Response_to_Reviewer_Comments_Revision_1 [file giz073_response_to_reviewer_comments_revision_1.pdf]

Author's Response To Reviewer Comments

Close

April 21, 2019

Dear Editorial Board of GigaScience,

We are very pleased to hear that our manuscript is conditionally accepted for publication. We thank you and the reviewers for their time and effort.

We have addressed the points noted by Reviewer #2 in the newly revised version submitted here. Further, we have properly formatted the reference section.

Our data is already deposited in the GigaDB repository. Once we hear back from the journal's curators, we will work with them to get the DOI and cite it in the manuscript.

Sincerely,

Paul Stothard
(Corresponding author on behalf of all authors)

==
Response to Reviewer #2:
==

We have corrected the typos in this revised version. Thank you for your thorough review.
L257: changed CVNR to CNVR.
L258: added '. ' at end of the sentence.

Close
