# Supplementary material for: A large interactive visual database of copy number variants discovered in taurine cattle
Source: Gigascience. 2019 Jun 26;8(6):giz073. doi: 10.1093/gigascience/giz073 (PMC6593363; doi:10.1093/gigascience/giz073)
Supplement: giz073_Reviewer_1_Report_Original_Submission — Christine Couldrey -- 11/2/2018 Reviewed [file giz073_reviewer_1_report_original_submission.pdf]

Reviewer Report

Title: A large interactive visual database of copy number variants discovered in taurine cattle

Version: Original Submission **Date:** 11/2/2018

Reviewer name: Christine Couldrey

Reviewer Comments to Author:

A large interactive visual database of copy number variants discovered in taurine cattle.
This manuscript describes the discovery and genotyping of copy number variations (CNVs) in multiple datasets using a single CNV algorithm together with the development of a platform that integrates multiple tools to allow identified CNVs to be easily assessed visually in multiple ways. The development of the platform will be of great use to the field of CNVs as streamlines the otherwise laborious task of characterising individual CNVs. The manuscript describes selected CNVs as examples of this platform. The manuscript is well written and represents a significant advance to the field.
There are some points in the manuscript I feel could be improved upon:
Lines 45-48 The authors say they are presenting the "the largest CNV set". However, no genome wide validation of the CNVs appears to have been undertaken (although the database described is set up in a manner to allow researchers to validate). Given that CNV discovery is plagued by false positives having the "largest set of CNVs " does not necessarily mean the best set - producing the largest set of putative CNVs is easy, but determining which are real, is hard. These sentences should be rewritten, particularly given that later in the manuscript the authors do acknowledge gaps affect the detection of CNVs.
Line 75-81 The authors indicate that there has been no genome wide validation of CNVs called by various CNV algorithms, however, Couldrey et al 2017 - this paper did successfully undertake genome wide assessment (Detection and assessment of copy number variation using PacBio long-read and Illumina sequencing in New Zealand dairy cattle.). The introduction should be amended to include this information, the manner in which the authors wrote this sentence implies that they have undertaken genome wide validation which they have not done.
Please include further discussion about the accuracy of breakpoints for CNVs and CNVRs, are these consistent between samples, if not, how does this affect analysis between samples and accuracy of assigned copy number genotype? Please also comment further on how the same genomic location was defined, if a CNVR in one animal was a subset of a larger one in another (or there was an overlap between animals) were these counted as unique or separate CNVRs
Line 214 please indicate if the CNVR upstream of KIT is the causative as determined by J. Taylors lab or another CNVR in the same area (also reported by the Taylor lab).
Given that historically deletions have been significantly easier to detect from short read sequence using read depth please add some discussion on the low frequency of deletion detection relative to duplication

Methods

Are the methods appropriate to the aims of the study, are they well described, and are necessary controls included? Choose an item.

Conclusions

Are the conclusions adequately supported by the data shown? Choose an item.

Reporting Standards

Does the manuscript adhere to the journal's guidelines on minimum standards of reporting? Choose an item.

Choose an item.

Statistics

Are you able to assess all statistics in the manuscript, including the appropriateness of statistical tests used? Choose an item.

Quality of Written English

Please indicate the quality of language in the manuscript: Choose an item.

Declaration of Competing Interests

Please complete a declaration of competing interests, considering the following questions:

- Have you in the past five years received reimbursements, fees, funding, or salary from an organisation that may in any way gain or lose financially from the publication of this manuscript, either now or in the future?
- Do you hold any stocks or shares in an organisation that may in any way gain or lose financially from the publication of this manuscript, either now or in the future?
- Do you hold or are you currently applying for any patents relating to the content of the manuscript?
- Have you received reimbursements, fees, funding, or salary from an organization that holds or has applied for patents relating to the content of the manuscript?
- Do you have any other financial competing interests?
- Do you have any non-financial competing interests in relation to this paper?

If you can answer no to all of the above, write 'I declare that I have no competing interests' below. If your reply is yes to any, please give details below.

'I declare that I have no competing interests'

I agree to the open peer review policy of the journal. I understand that my name will be included on my report to the authors and, if the manuscript is accepted for publication, my named report including any attachments I upload will be posted on the website along with the authors' responses. I agree for my

report to be made available under an Open Access Creative Commons CC-BY license (http://creativecommons.org/licenses/by/4.0/). I understand that any comments which I do not wish to be included in my named report can be included as confidential comments to the editors, which will not be published.

I agree to the open peer review policy of the journal

To further support our reviewers, we have joined with Publons, where you can gain additional credit to further highlight your hard work (see: https://publons.com/journal/530/gigascience). On publication of this paper, your review will be automatically added to Publons, you can then choose whether or not to claim your Publons credit. I understand this statement.

Yes Choose an item.
