# Supplementary material for: A large interactive visual database of copy number variants discovered in taurine cattle
Source: Gigascience. 2019 Jun 26;8(6):giz073. doi: 10.1093/gigascience/giz073 (PMC6593363; doi:10.1093/gigascience/giz073)
Supplement: giz073_Reviewer_2_Report_Original_Submission — Aniek Bouwman -- 11/17/2018 Reviewed [file giz073_reviewer_2_report_original_submission.pdf]

Reviewer Report

Title: A large interactive visual database of copy number variants discovered in taurine cattle

Version: Original Submission **Date:** 11/17/2018

Reviewer name: Aniek Bouwman

Reviewer Comments to Author:

This paper describes CNV(R) detection in sequenced cattle of four different datasets. It highlights some important issues that may arise during CNV detection in data from different sequencing platforms, but also in CNV detection in general. The authors have provided a valuable database for future research that can serve as a resource to confirm CNVs and visualize them.
I have not been able to find the actual database on GigaDB. This makes it hard to value the usefulness. My main remark is that there are a number of recommendations done, for which the reason and tools to do so are lacking (e.g., HWE) or unrealistic (e.g., confirm genome wide CNV visually), see the line by line comments. There are many methods to predict CNV, the authors seem very confident about their approach, but other than the platform effect, there are limited results shown to back that. Visualizing seems to be the key. However, this is very unpractical when screening the whole genome of hundreds of individuals. On the other hand, when interested in a specific CNV visualisation to confirm the CNV is needed, and then this database would be useful for confirmation.
Line Comment
L122 You refer to 'batch effect' here however to me it seems a sequencing platform effect. Can different batches sequenced on the same platform with similar coverage be combined? Was that the case here? With different batches I mean a group of animals sequenced in January and another group in June for instance (same platform, same intended coverage).
L122 Please indicate that the CNV discovery referred to here was done in the complete dataset
L129 Change figure 1b into figure 1a
L152-153 When you remove samples should you not rerun CNV detection?
L243-244 Which version number of Chrome/Firefox is that? Or will this be true in the future as well?
L254 Does this mean that the CNVs detected with UMD3.1 can now be plotted in the new genome build ARS-UCD 1.2?
L261 Were HWE and parity test part of the QC mentioned in table 2?
L264-266 This sentence confused me. If 63-88% of the CNVR that passed HWE had a p-value >=10exp-5 what was then your threshold to qualify HWE? It is also not given in M&M.
L268-270 Why do you recommend this and how should one verify this? It seems that you know something you forgot to inform us about. Why not simply discard them, as we do with SNPs?
L287-288 I didn't see this discussion in previous section. Yes HWE was mentioned but there is no proper discussion on the reasons of deviation from HWE and how your database can verify them. So please add. See also previous comment.
L313 Remove additional '.' at end of sentence
L386-387 Can you back this superiority with a reference? Are methods that combine for instance read

depth and paired end reads not better?
L410-411 Interesting comment about imputation. Like to get your thoughts on this subject. Based on what can you perform imputation for CNV? Pedigree? SNPs? The SNP-CNV LD seems very limited for imputation, but perhaps there is more information in sequence data.
L427-429 Do you mean visualizing each CNVR one by one? Noble recommendation, but that is a pain in the ass job, for instance for 26000 CNVR detected in your study. Have you visualized them all? I assume only the ones you report in detail about. Isn't that the reason why we apply methods like cn.MOPS, to automate the job. I know that CNV calling methods have their issues, but on a genome wide scale checking each detected CNV manually seems a bit too much to do, especially as sample sizes are getting bigger and bigger. I agree that CNVR of interest that will be explored further because of association or something, they should be manually checked, but genome wide is a bit much I would say, unless you can automate it.
L457 Can you add why you chose cn.MOPS? There are so many methods, and as you indicated the method is relevant.
Figure 4 more distinctive colours for the bars would be appreciated
Figure 5 Hard to see what is in the panels, and the database itself I could not find on GigaDB. Also in Supplementary material figure S12-16.

Methods

Are the methods appropriate to the aims of the study, are they well described, and are necessary controls included? Choose an item.

Conclusions

Are the conclusions adequately supported by the data shown? Choose an item.

Reporting Standards

Does the manuscript adhere to the journal's guidelines on minimum standards of reporting? Choose an item.

Choose an item.

Statistics

Are you able to assess all statistics in the manuscript, including the appropriateness of statistical tests used? Choose an item.

Quality of Written English

Please indicate the quality of language in the manuscript: Choose an item.

Declaration of Competing Interests

Please complete a declaration of competing interests, considering the following questions:

- Have you in the past five years received reimbursements, fees, funding, or salary from an organisation that may in any way gain or lose financially from the publication of this manuscript, either now or in the future?
- Do you hold any stocks or shares in an organisation that may in any way gain or lose financially from the publication of this manuscript, either now or in the future?
- Do you hold or are you currently applying for any patents relating to the content of the manuscript?
- Have you received reimbursements, fees, funding, or salary from an organization that holds or has applied for patents relating to the content of the manuscript?
- Do you have any other financial competing interests?
- Do you have any non-financial competing interests in relation to this paper?

If you can answer no to all of the above, write 'I declare that I have no competing interests' below. If your reply is yes to any, please give details below.

I declare that I have no competing interests

I agree to the open peer review policy of the journal. I understand that my name will be included on my report to the authors and, if the manuscript is accepted for publication, my named report including any attachments I upload will be posted on the website along with the authors' responses. I agree for my report to be made available under an Open Access Creative Commons CC-BY license (http://creativecommons.org/licenses/by/4.0/). I understand that any comments which I do not wish to be included in my named report can be included as confidential comments to the editors, which will not be published.

I agree to the open peer review policy of the journal

To further support our reviewers, we have joined with Publons, where you can gain additional credit to further highlight your hard work (see: https://publons.com/journal/530/gigascience). On publication of this paper, your review will be automatically added to Publons, you can then choose whether or not to claim your Publons credit. I understand this statement.

Yes Choose an item.
